# Visible-Light-Mediated Deaminative Alkylation of Primary Amines with Silacarboxylic Acids via Isonitrile Formation

**DOI:** 10.1021/acs.orglett.4c04214

**Published:** 2025-01-07

**Authors:** Carla Pérez-Sánchez, Thomas Rigotti, Mariola Tortosa

**Affiliations:** † Organic Chemistry Department, Faculty of Science, 16722 Autonomous University of Madrid, 28049 Madrid, Spain; ‡ Center for Innovation in Advanced Chemistry (ORFEO−CINQA), Autonomous University of Madrid, 28049 Madrid, Spain; § Institute for Advanced Research in Chemical Sciences (IAdChem), Autonomous University of Madrid, 28049 Madrid, Spain

## Abstract

The functionalization of the C–N bond of amines is a straightforward strategy for the construction of complex scaffolds or for the late-stage functionalization of pharmaceuticals. Herein, we describe a photoredox-catalyzed strategy for the deaminative alkylation of primary amine-derived isonitriles that provides unnatural amino acid derivatives under mild conditions. The use of silacarboxylic acids as silyl radical precursors enables the generation of carbon-centered radicals that allow the construction of Csp^^3^^–Csp^^3^^ bonds via a Giese-type addition, avoiding the undesired hydrodeamination product.

## Graphical abstract

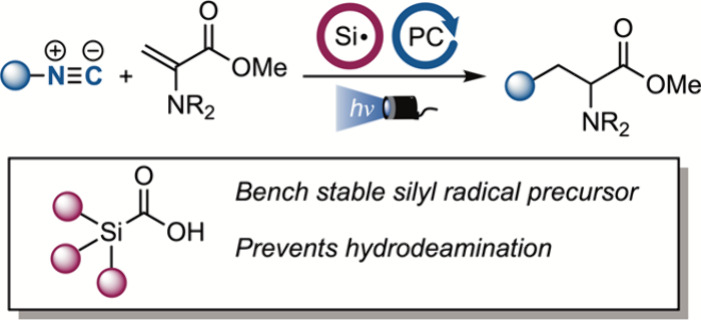

Isonitriles are chameleonic functional groups that display unusual electronic properties and reactivities, making them interesting compounds in organic synthesis.^1–3^ Indeed, although isoelectronic to alkynes, their polar and radical reactivities are drastically different, being characterized by 1,1-additions.^4^ Moreover, the ability of isonitriles to interact with both donor and acceptors in radical reactions accounts for their versatility as building blocks in radical chemistry. Although isonitriles display a growing interest in medicinal chemistry^5^ and can be easily obtained from commercially available and ubiquitous amines,^6,7^ their use in catalytic C–N cleavage/C–C bond formation remains rather underexplored.^8^ Inspired by the classical Barton–Saegusa deamination,^9^ we recently developed a light-mediated strategy for the generation of carbon-centered radicals from primary amine-derived isonitriles (Scheme 1).^10^ (TMS)_3_SiH (supersilane) was used as silyl radical precursor and hydrogen atom donor, establishing a general C–N bond activation mode in photocatalysis.^11,12^ To expand the range of transformations that employ isonitriles in light-driven transformations,^13^ we decided to investigate the possibility to use them for the construction of Csp_^3^_–Csp_^3^_ bonds via Giese-type additions, complementing the deaminative alkylation reported with pyridinium salts and redox-active imines.^14^ In particular, we were interested in using dehydroalanine derivatives as radical acceptors to prepare non-natural amino acids from primary amines via isonitrile formation. Unfortunately, when we used the conditions previously developed for the hydrodeamination reaction in the presence of a Giese acceptor, we only observed low yields of the desired product (Scheme 1). Under these conditions, alternative reaction pathways appeared to be operative, leading to a significant hydrogen abstraction from supersilane before any C–C bond formation could take place and addition of the silyl radical to the acceptor as the major product. During the preparation of this manuscript, Tian and Zhang reported a radical-chain Giese-type reaction with isonitriles and supersilane as the silyl radical precursor.^15^ Although they observed moderate yields, the optimized conditions required 5 equiv of isonitrile, which is far from ideal to be used in the late-stage functionalization of advanced intermediates. Our goal from the outset was to provide a protocol that avoided the undesired hydrodeamination pathway while maintaining the valuable isonitrile as the limiting reagent. To solve this issue, we decided to explore alternative silyl radical precursors that could be engaged in a photoredox catalytic cycle. Specifically, we hypothesized that a suitable silyl radical could be generated from silacarboxylic acids by single-electron oxidation, followed by decarboxylation (Scheme 1).^16,17^ This open-shell species could add to the isonitrile to form an imidoyl radical that would generate a carbon-centered radical upon β-scission.^18^ In the end, this nucleophilic species would add to the Giese acceptor giving a radical intermediate that could be reduced to regenerate the ground-state photocatalyst while delivering the product. In this scenario, the deamination pathway would be suppressed by the absence of labile abstractable hydrogens. Indeed, considering that the bond dissociation energy (BDE) for a carboxylic acid O–H bond should be >110 kcal/mol,^19^ a direct hydrogen atom transfer should be precluded,^20^ in contrast with the facile abstraction from supersilane (Si–H BDE = 84 kcal/mol).^21^

**Scheme 1:**
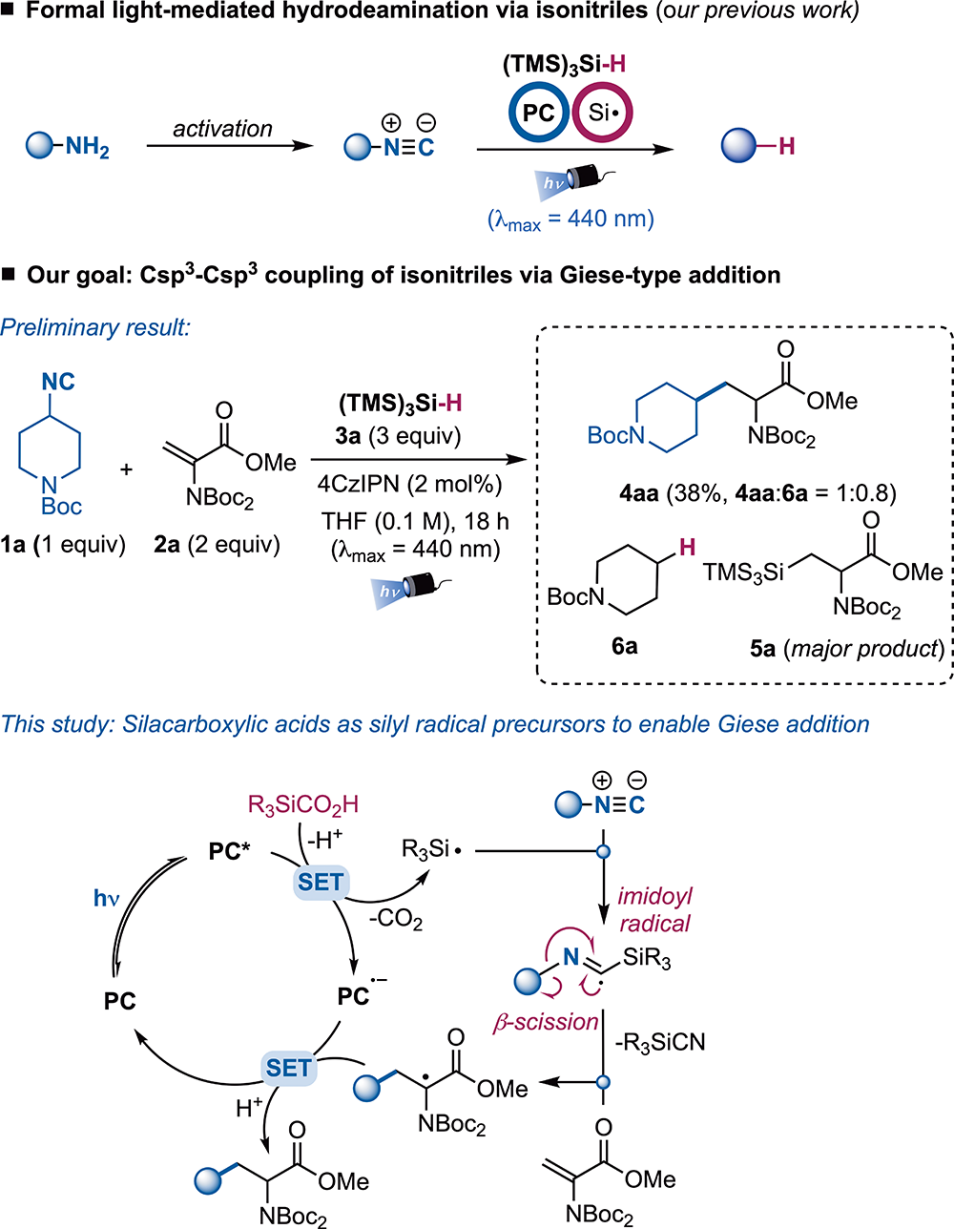
Silacarboxylic acids as Silyl Radical Precursors for the Construction of Csp^3^–Csp^3^ Bonds via Giese-Type Addition

We started our investigations employing *tert*-butyl 4-isocyanopiperidine-1-carboxylate **1a** and *N*-Boc_2_-dehydroalanine-OMe **2a** as model substrates (Table 1).^22^ As mentioned above, employing (TMS)_3_SiH **3a** we only obtained the desired product **4aa** in 38% yield (entry 1 in Table 1), along with a large amount of the undesired hydrodeamination product **6a** and silyl radical addition adduct **5**.^23^ We then tested (TMS)_3_SiNHAd **3b**, recently used in our group to promote de heteroarylation of isonitriles, observing similar poor results (entry 2 in Table 1). Only 27% of conversion was achieved with an unfavorable **4aa**:**6a** ratio (0.7:1) toward the undesired Csp^3^–H bond formation. We reasoned that the presence of a labile Si–H bond in **3a** and the presence of abstractable hydrogens in **3b** (upon SET to the corresponding radical cation), could interfere with the desired reaction pathway. Gratifyingly, when *t*-BuPh_2_SiCO_2_H **3c** was employed (entry 3 in Table 1) full conversion of isonitrile **1a** was achieved, obtaining the desired product **4aa** in 91% yield and avoiding the formation of **6a**.^24^ Other similar silacarboxylic acids were tested (entries 4–6 in Table 1) but provided slightly lower yields or a complex mixture of products. Different solvents such as acetone and acetonitrile were tested but furnished the product with a diminished yield (entries 7 and 8 in Table 1). Additionally, we observed that the reaction was complete after 2 h with the same efficiency (entry 9 in Table 1). Finally, the presence of light and of the silyl radical precursor **3c** was evaluated, indicating that both components were necessary for the reaction to take place, which confirmed the photocatalytic nature of the radical process (entries 10 and 11 in Table 1).

**Table 1:**
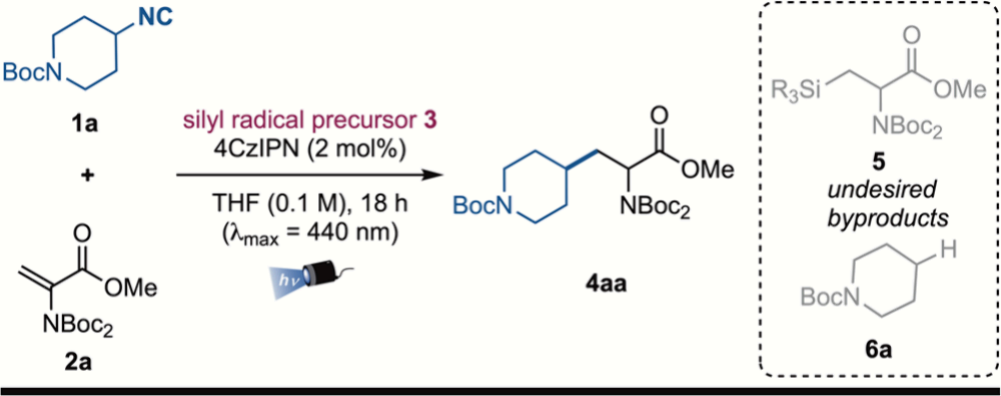
Reaction Optimization^a^

entry	silyl radical precursor (**3**)		conversion^b^	**4aa**:**6a** ratio^b^	yield of **4aa** ^c^
1​	(TMS)_3_SiH	**3a**	>98	1:0.8	38
2​	(TMS)_3_SiNHAd	**3b**	27	0.7:1	nd[Table-fn t1fn100]
3​	*t*-BuPh_2_SiCO_2_H	**3c**	>98	1:0	91
4​	MePh_2_SiCO_2_H	**3d**	>98	1:0.1	85
5​	Me_2_PhSiCO_2_H	**3e**	>98	1:0.4	72
6​	(TMS)_3_SiCO_2_H	**3f**	cm[Table-fn t1fn101]	—	—
7​^d^	*t*-BuPh_2_SiCO_2_H	**3c**	>98	1:0.1	88
8​^e^	*t*-BuPh_2_SiCO_2_H	**3c**	>98	1:0.1	82
9​^f^	*t*-BuPh_2_SiCO_2_H	**3c**	>98	1:0	91
10​^g^	*t*-BuPh_2_SiCO_2_H	**3c**	0	–	–
11​	–		0	–	–

a The reactions were performed employing isonitrile **1a** (0.05 mmol), Giese acceptor **2a** (0.10 mmol), silyl radical precursor **3** (0.15 mmol), and 1,2,3,5-tetrakis­(carbazol-9-yl)-4,6-dicyanobenzene (4CzIPN) as the photoredox catalyst (2 mol %) in THF (0.5 mL, *c* = 0.1 M) under blue LEDs irradiation (λ = 440 nm).

b Determined by ^1^H NMR analysis.

c Isolated yield after purification by flash chromatography.

d MeCN as solvent.

e Acetone as solvent.

f Reaction time: 2 h.

g Reaction performed in darkness.

h nd = not determined.

i cm = complex mixture.

With the optimized conditions in hand, the scope of the reaction was evaluated, providing the desired products **4** in moderate to excellent yields (Scheme 2). Initially, different isonitriles were tested employing *N*-Boc_2_-dehydroalanine-OMe **2a** as the Giese acceptor to obtain a variety of unnatural amino acid derivatives **4aa**–**4oa**. The reaction worked with *N*-Boc-piperidine-, tetrahydropyran-, and sulfone-containing secondary isonitriles, providing products **4aa**–**4ca** in excellent yields. It is worth noting that the model reaction could be easily performed on a 1.0 mmol scale to obtain product **4aa** with the same yield (91%), highlighting the synthetic applicability of the present methodology. Different ring sizes and strained bicyclic structures could be incorporated in the products, as exemplified by the *N*-Boc-azetidine and bicyclo[1.1.1]­pentane derivatives **4da** and **4ea**, which were obtained in yields of 95% and 93%, respectively.

**Scheme 2:**
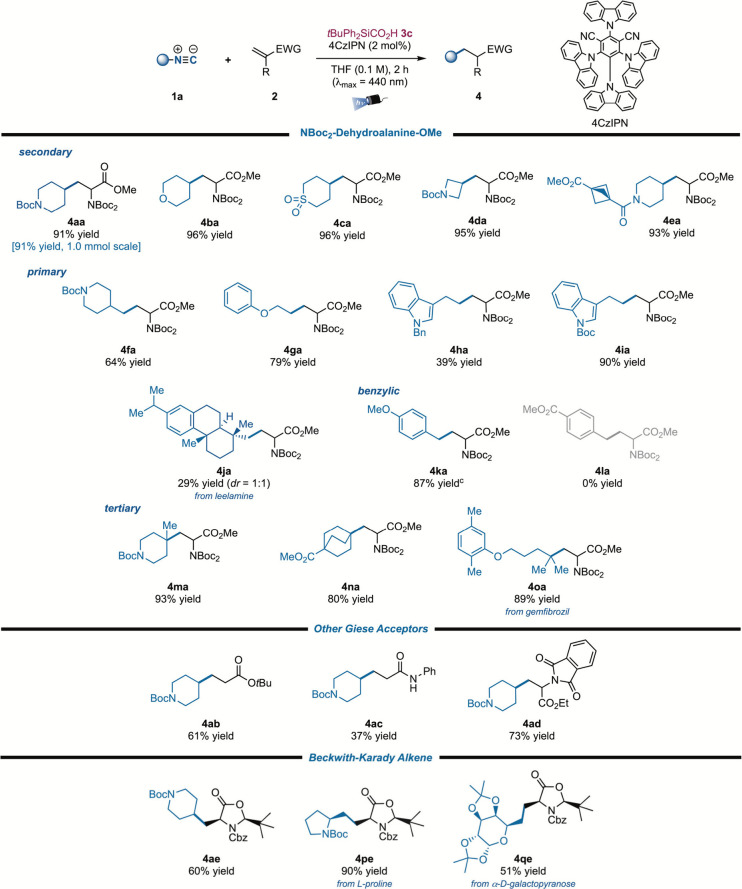
Scope of the Deaminative Alkylation via Isonitriles^a^ ^,^ ^b^

a The reactions were performed employing isonitrile **1** (0.10 mmol), Giese acceptor **2** (0.20 mmol), silyl radical precursor **3c** (0.30 mmol), and 4CzIPN (2 mol %) in THF (1.0 mL, *c* = 0.1 M) under blue LEDs irradiation (λ = 440 nm).
b Isolated yield after purification by flash chromatography or preparative thin layer chromatography.
c Four equiv of **3c** (0.40 mmol) and 4 h of irradiation were employed.

Primary isonitriles which gave rise to less stable primary radicals could be employed as well, delivering products **4fa**–**4ja** in moderate to good yields, including indole and natural product (leelamine) derivatives (**4ha**–**4ja**). Benzylic radicals with electron-donating groups efficiently took part in the Giese-type addition, as showcased by the obtainment of product **4ka** (87% yield). However, the presence of electron-withdrawing diminishes the nucleophilicity of the carbon-centered radical and suppresses the addition to the poor radical acceptor (0% yield for **4la**). More sterically hindered tertiary isonitriles were suitable reaction partners that provide the corresponding adducts **4ma**–**4oa** in excellent yields, including a bicyclo[2.2.2]­octane and a gemfibrozil derivative. On the other hand, different Giese acceptors (**2b**–**2d**) were evaluated. The more electrophilic *t*Bu-acrylate **2b** behaved as an efficient reaction partner, delivering the product **4ab** with a 61% yield, whereas *N*-Ph-acrylamide **2c** led to the corresponding product **4ac** only in moderate yield. Furthermore, the *N*-phthalimide dehydroalanine derivative **2d** performed similarly to the model acceptor to give access to product **4ad** in 73% yield, which represents an *N*-phthalimide protected unnatural amino acid derivative. Additionally, a diastereoselective process that gives access to enantioenriched unnatural amino acid derivatives could be developed. Employing the Beckwith–Karady alkene **2e** the corresponding products **4ae**, **4pe**, and **4qe** were obtained with complete diastereoselectivity (dr > 98:2).^22,25^ Among them, compounds **4pe** and **4qe** were obtained in 90% and 51% yield starting from l-proline- and α-d-galactopyranose-derived isonitriles **1p** and **1q**, showcasing the possibility to apply this methodology to more-complex scaffolds.

Specifically, the radical conjugate addition to the Beckwith–Karady alkene **2e** gives access to a stabilized electrophilic radical which is prone to undergo single-electron transfer reduction to the corresponding enolate (Scheme 3). This species will be subsequently protonated through the less-hindered face due to the presence of the bulky *tert*-butyl group, giving rise to a highly diastereoselective process.

**Scheme 3:**
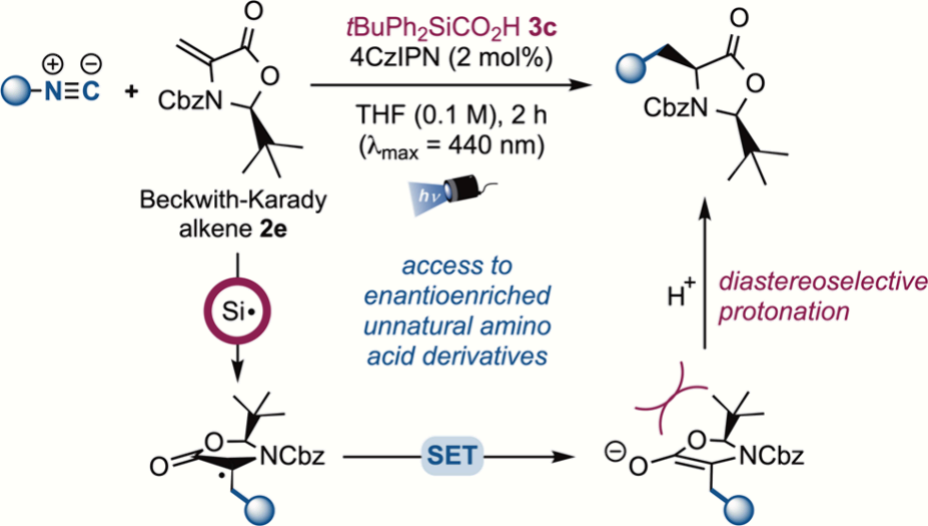
Diastereoselective Protonation upon Radical Conjugate Addition to Beckwith–Karady Alkene

Some mechanistic investigations were carried out to clarify the reaction pathway and the mechanistic proposal for the Giese-type addition via isonitriles, as depicted in Scheme 4. Upon visible-light irradiation, the excited photocatalyst [*E*_red_^*^(4CzIPN*/4CzIPN^• –^) = +1.35 V vs SCE] can engage in a SET with the deprotonated silacarboxylic acid **3c**, giving a silyl-centered radical upon facile decarboxylation [*E* ^1/2^(R_3_SiCO_2_^•^/R_3_SiCO_2_^–^) ≈ +1.3 V vs SCE]. Thus, the *tert*-butyldiphenylsilyl radical adds to the isonitrile to give an imidoyl radical which can undergo a β-scission to obtain a carbon-centered radical on the α-nitrogen carbon of the starting isonitrile. This nucleophilic species will then add to the Giese acceptor in a radical conjugate addition that provides an electrophilic (and stabilized) radical intermediate, which will be reduced by the radical anion of 4CzIPN to provide (upon protonation) the desired product and restore the ground state photocatalyst [*E*_red_(4CzIPN/4CzIPN^• –^) = −1.21 V vs SCE]. This picture was confirmed by Stern–Volmer quenching studies, which identified the carboxylate formed from acid **3c** as the only compound interacting with the excited-state photocatalyst. Indeed, quenching was not observed for isonitrile **1a** and for Giese acceptor **2a**, in accordance with the high redox potentials required to oxidize or reduce these reaction partners.

**Scheme 4:**
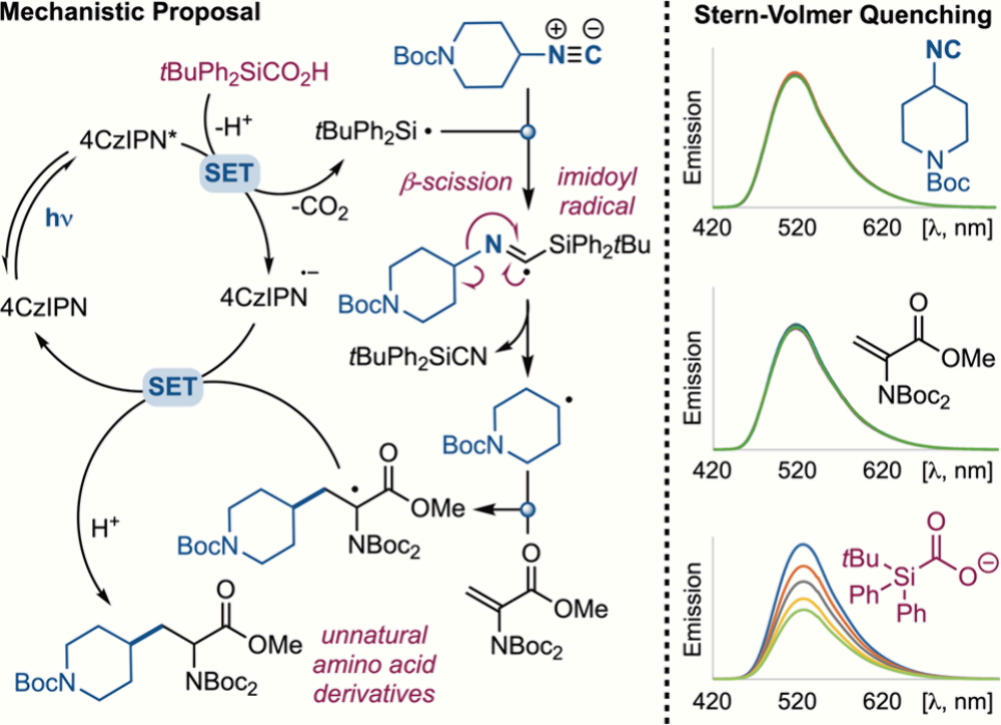
Mechanistic Proposal and Fluorescence Quenching Experiments

In conclusion, a novel photocatalytic Giese-type reaction to prepare unnatural amino acid derivatives has been developed. This strategy relies on the C–N bond activation of isonitriles to give carbon-centered radicals from ubiquitous primary amines via redox-neutral compounds. The use of silacarboxylic acids as suitable silyl radical precursors enables an efficient radical conjugate addition, avoiding undesired reaction pathways that take place with other silyl radical mediators.

## Supplementary Material



## Data Availability

The data underlying this study are available in the published article and its online Supporting Information.

## Notes

Materials and methods, synthetic procedures, optimization of reaction conditions and full characterization of new compounds (PDF)
