# Supplementary material for: Visible-Light-Mediated Deaminative Alkylation of Primary Amines with Silacarboxylic Acids via Isonitrile Formation
Source: Org Lett. 2025 Jan 7;27(2):583–7. doi: 10.1021/acs.orglett.4c04214 (PMC12140405; doi:10.1021/acs.orglett.4c04214)

Supporting Information for

Visible Light-Mediated Deaminative Alkylation of Primary Amines with Silacarboxylic Acids via Isonitrile Formation

Carla Pérez-Sánchez,[a] Thomas Rigotti,*,[a] and Mariola Tortosa*,[a,b,c]

[a] Organic Chemistry Department, Faculty of Science, Autonomous University of Madrid, 28049, Madrid (Spain)

[b] Center for Innovation in Advanced Chemistry (ORFEO-CINQA), Autonomous University of Madrid, 28049, Madrid (Spain).

[c] Institute for Advanced Research in Chemical Sciences (IAdChem), Autonomous University of Madrid, 28049, Madrid (Spain).

1. General Considerations 1
2. General Procedures for the Synthesis of Starting Materials 2
   2.1. Synthesis of methyl methyl 3-(4-aminopiperidine-1-carbonyl)bicyclo[1.1.1]pentane-1-carboxylate (**SI-2**) 3
   2.2. Synthesis of methyl 4-aminobicyclo[2.2.2]octane-1-carboxylate (**SI-4**) 4
   2.3. Synthesis of 5-(2,5-dimethylphenoxy)-2-methylpentan-2-amine (**SI-6**) 5
   2.1. General Procedure A for the Synthesis of Isonitriles **1** from Primary Amines (**GP-A**) 6
   2.2. General Procedure B for the Synthesis of Isonitriles **1** from Primary Amines (**GP-B**) 7
   2.3. Characterization Data for Isonitriles **1** 8
3. Deaminative Alkylation 11
   3.1. Reaction Setup 11
   3.2. Reaction Optimization 12
   3.3. General Procedure for the Deaminative Alkylation (**GP-C**): 14
   3.4. Characterization Data for Alkylation Products 15
   3.5. Scale-Up Synthesis of **4aa** 26
4. Mechanistic Studies 27
   4.1. Mechanistic Proposal for the Deaminative Alkylation 27
   4.2. Additional Experiments 28
   4.3. Quantum Yield 29
   4.4. Stern-Volmer Experiment 33
5. NMR Spectra 36

1. General Considerations

All reactions sensitive to air or moisture were carried out in flame-dried glassware under argon pressure using standard Schlenk techniques. Tetrahydrofuran (THF), toluene and acetonitrile (MeCN) were purified by passing through a Pure Solv™ column drying system from Innovative Technology, Inc. Dry acetone, dimethylformamide (DMF), dimethylsulfoxide (DMSO), and diethyl ether were purchased from Acros Organics and employed without further purification.

Flash column chromatography was performed on silica gel 60 (VWR, 230-400 mesh) or on RediSep® Bronze columns (Teledyne, ISCO, 230-400 mesh) with the indicated eluent mixtures. Thin layer chromatography (TLC) was performed on silica coated aluminum plates (silica 60 F254) with detection by UV-light (λ = 254 nm) and employing potassium permanganate ($KMnO_4$), phosphomolybdic acid, ninhydrin, vanillin or cerium ammonium molybdate (CAM) stain developer solutions followed by heat treatment. Agilent Bond Elut SCX cartridges were employed for the separation of specific amine-containing compounds.

NMR spectra were acquired on a Bruker Avance 300 MHz spectrometer running at 300 and 75 MHz for ^1H and ^{13}C, respectively or a 500 MHz spectrometer running at 500 and 126 MHz for ^1H and ^{13}C, respectively. Chemical shifts (δ) are reported in ppm relative to the respective residual solvent signals of $CDCl_3$ [δ (^1H) = 7.26 ppm, δ (^{13}C) = 77.16 ppm], THF-d8 [δ (^1H) = 3.58 and 1.73 ppm] or tetrachloroethane-d2 [δ (^1H) = 5.91 ppm, δ (^{13}C) = 74.20 ppm]. The temperature for the acquisition of the NMR spectra was indicated in each case, if different from room temperature (298 K). Coupling constants are given in Hz. The following abbreviations are used to indicate the multiplicity: s, singlet; d, doublet; t, triplet; q, quartet; m, multiplet; br, broad signal. ^{13}C NMR and ^{19}F spectra were acquired on a broad band decoupled mode. High Resolution Mass Spectrometry (HRMS) were registered in a GCT Agilent Technologies 6890 N spectrometer using Electronic Impact (EI+) techniques at 70 eV and electrospray (ESI+) or Bruker maXis II™ (APCI+). Melting points were determined in a Stuart™ melting point SMP3 apparatus in open capillary tubes. UV/vis studies and fluorescence measurements were measured in a 1 cm quartz cuvette using a JASCO V-660 UV/vis spectrophotometer and a JASCO FP-8600 spectrofluorometer, respectively. Gas Chromatography (GC) analysis and Low-Resolution Mass Spectrometry (LRMS) was performed on an Agilent 7820A gas chromatograph using an Agilent HP-5MS UI column with a 5977A single quadrupole mass detector.

2. General Procedures for the Synthesis of Starting Materials

Isonitriles, silacarboxylic acids and electron poor olefins (Figure S1) were prepared according to literature procedures, purchased from suppliers, or synthesized as below.

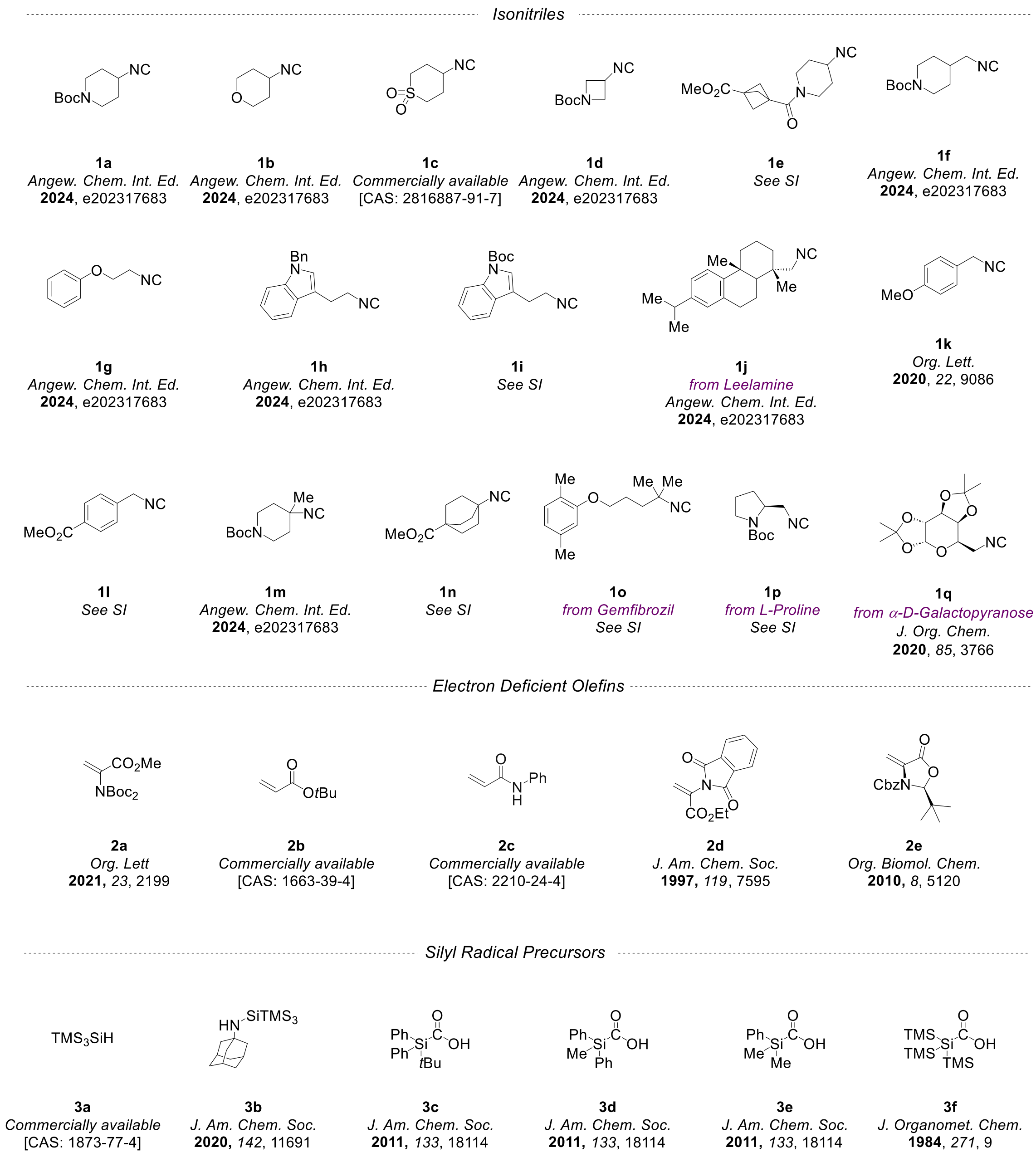

Figure S1. Synthesis of starting materials

2.1. <u>Synthesis of methyl methyl 3-(4-aminopiperidine-1-carbonyl)bicyclo[1.1.1]pentane-1-carboxylate (**SI-2**)</u>

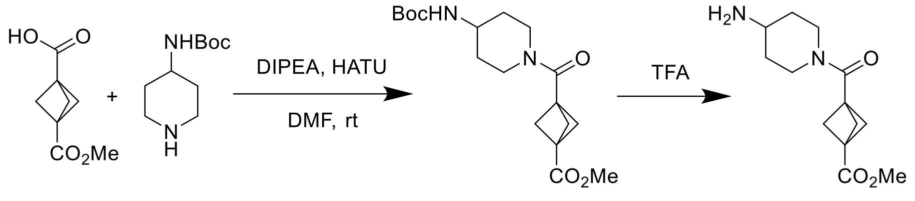

SI-1 **SI-2**

To an oven dried round bottom flask equipped with a magnetic stir bar were added 3-(methoxycarbonyl)bicyclo[1.1.1]pentane-1-carboxylic acid (500.0 mg, 2.94 mmol, 1 equiv.), *tert*-butyl piperidin-4-ylcarbamate (1.18 g, 2 equiv.) and HATU (1.34 g, 1.2 equiv.). Then the flask was evacuated and refilled with argon before dry DMF (36.7 mL, 0.08 M) was added. After stirring for 5 minutes, DIPEA (2.30 mL, 4.5 equiv.) was added by syringe and the mixture was stirred at rt for 12 hours. Then, the reaction mixture was diluted with water and EtOAc. The aqueous phase was extracted 5 times with EtOAc and the combined organic phases were washed with brine 3 times before being dried over sodium sulphate. The solvents were removed under reduced pressure and the obtained residue was used in the next step without further purification.

To a solution of the residue **SI-1** in DCM (0.15 M) was added a solution of trifluoroacetic acid in DCM (2.00 mL, 2.6 M, 10 equiv.) and the reaction mixture was stirred for 6 h before removing the volatiles under reduced pressure. Then, DCM and a saturated solution of Na_2CO_3 were added, and the aqueous phase was extracted with DCM two times. The combined organic phases were washed with brine and dried over Na_2SO_4. After removing the solvents under reduced pressure, the crude product **SI-2** was obtained and used in the next step without further purification.

2.2. Synthesis of methyl 4-aminobicyclo[2.2.2]octane-1-carboxylate (**SI-4**)

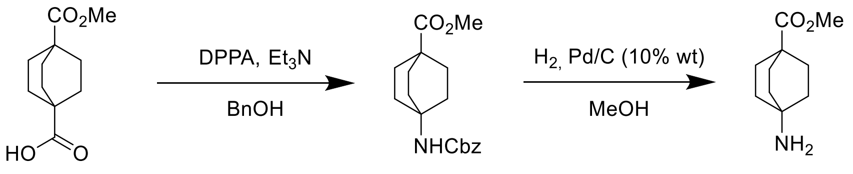

SI-3 **SI-4**

In an oven dried Schlenk tube, 4-(methoxycarbonyl)bicyclo[2.2.2]octane-1-carboxylic acid (2.00 g, 9.43 mmol, 1.00 equiv.) was dissolved in toluene (18.8 mL, 0.5 M) under argon. Then, triethylamine (2.00 mL, 14.1 mmol, 1.5 equiv.), benzyl alcohol (2.00 mL, 18.9 mmol, 2 equiv.) and diphenyl phosphoryl azide (2.40 mL, 11.3 mmol, 1.2 equiv.) were subsequently added at rt under stirring. The reaction mixture was stirred at reflux (110 ºC) for 16 h, observing full conversion of the carboxylic acid as judged by TLC analysis. The solvent was removed under reduced pressure and EtOAc was added to the residue. The solution was washed with brine, dried over anhydrous Na_2SO_4 and concentrated under reduced pressure. The residue **SI-3** was directly used in the next step without further purification.

The residue **SI-3** was dissolved in MeOH (20 mL) and palladium on charcoal was added (1.00 g, 10% wt., 0.94 mmol Pd). The mixture was shaken in a Parr hydrogenation apparatus under a pressure of hydrogen (50 psi) for 16 h (full conversion as judged by TLC analysis). Thus, the mixture was filtered through a pad of Celite©, rinsed with MeOH (5 mL) and concentrated under reduced pressure to give a residue which was purified with an *Agilent SCX Bond Elut* cartridge to obtain product **SI-4** as a white solid in 60% yield (1.01 g). R_F = 0.27 (4% MeOH in DCM).

^{1}H-NMR (300 MHz, $CDCl_3$) δ 3.59 (s, 3H), 2.00 (*br* s, 2H), 1.87-1.68 (m, 6H), 1.60-1.45 (m, 6H). Spectrum

Spectroscopic data are in agreement with those described in the literature.[1]

[1] Yeh, V. S. C.; Kurukulasuriya, R.; Kerdesky, F. A. *Org. Lett.* **2006**, *8*, 3963-3966.

2.3. Synthesis of 5-(2,5-dimethylphenoxy)-2-methylpentan-2-amine (**SI-6**)

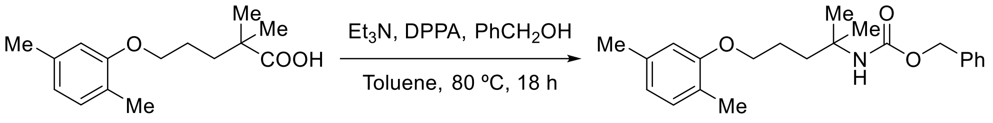

SI-5

According to a literature procedure,[2] gemfibrozil (1.25 g, 5.00 mmol, 1.0 equiv.), triethylamine (0.84 mL, 6.00 mmol, 1.2 equiv.) and diphenyl phosphoryl azide (1.51 g, 5.50 mmol, 1.1 equiv.) were stirred in toluene (50.0 mL) for 1 h at rt. Then, benzyl alcohol (2.6 mL, 25.0 mmol, 5.0 equiv.) was added and the reaction mixture was stirred at 80 ºC for 18 h. The crude reaction was concentrated and purified by flash column chromatography (SiO_2; 0-10% EtOAc in cyclohexane) to afford product **SI-5** as a slightly yellow oil (1.12 g, 63% yield).

^{1}H-NMR (300 MHz, $CDCl_3$): δ 7.42-7.31 (m, 5H), 7.04 (d, J = 7.4 Hz, 1H), 6.70 (d, J = 7.6 Hz, 1H), 6.65 (s, 1H), 5.09 (s, 2H), 4.78 (br s, 1H), 3.96 (t, J = 5.8 Hz, 2H), 2.35 (s, 3H), 2.22 (s, 3H), 1.91-1.78 (m, 4H), 1.37 (s, 6H). Spectrum

^{13}C-NMR (75 MHz, $CDCl_3$): δ 157.0, 154.7, 136.8, 136.5, 130.4, 128.6, 128.13, 128.11, 123.6, 120.8, 112.1, 68.0, 66.1, 52.8, 36.9, 27.2, 24.4, 21.5, 15.9. Spectrum

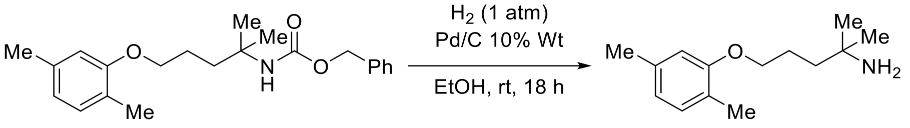

SI-5 **SI-6**

According to the literature procedure,[2] in a round bottom flask palladium on carbon (299.5 mg, 10% wt, 0.28 mmol Pd) was added followed by a solution of **SI-5** (1.00 g, 2.81 mmol, 1.0 equiv.) in EtOH (20.0 mL). The reaction mixture was purged with hydrogen, and it was stirred overnight under hydrogen atmosphere (1 atm). Upon reaction completion, the mixture was filtered through a plug of celite and concentrate to obtain the desired pure amine **SI-6** in 89% yield.

[2] Kulagowki, J. J.; Blair, W.; Bull, R.; Chang, C.; Deshmukh, G.; Dyke, H.; Eigenbrot, C.; Ghilardi, N.; Gibbons, P.; Harrison, T.; Hewitt, P.; Liimatta, M.; Hurley, C.; Johnson, A.; Johnson, T.; Kenny, J.; Kohli, P.; Maxey, R.; Mendonca, R.; Mortara, K.; Murray, J.; Narukulla, R.; Shia, S.; Steffek, M.; Ubhayakar, S.; Ultsch, M.; van Abbema, A.; Ward, S.; Waszkowycz, B.; Zak, M. *J. Med. Chem.* **2012**, *55*, 5901–5921.

^{1}H-NMR (300 MHz, $CDCl_3$): δ 6.99 (d, J = 7.5 Hz, 1H), 6.65 (d, J = 7.5 Hz, 1H), 6.60 (s, 1H), 4.10 (br s, 2H), 3.94 (t, J = 6.2 Hz, 2H), 2.30 (s, 3H), 2.17 (s, 3H), 1.93-1.81 (m, 2H), 1.70-1.60 (m, 2H), 1.24 (s, 6H). Spectrum

^{13}C-NMR (75 MHz, $CDCl_3$): δ 156.9, 136.4, 130.3, 123.6, 120.7, 112.0, 68.0, 50.6, 40.3, 29.1, 24.6, 21.4, 15.8. Spectrum

HRMS (ESI+): calculated for $C_{14}H_{24}NO$ $[M+H]^+$: 222.1852; found: 222.1857.

2.1. General Procedure A for the Synthesis of Isonitriles **1** from Primary Amines **(GP-A)**

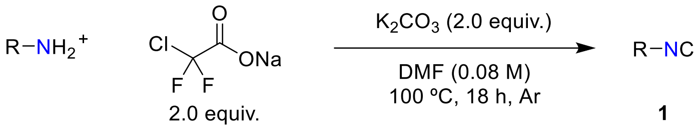

According to a literature procedure,[3] in an oven-dried Schlenk tube equipped with a magnetic stir bar were added the primary amine (1.0 equiv.), sodium chlorodifluoroacetate (2.0 equiv.) and K_2CO_3 (2.0 equiv.). The Schlenk tube was evacuated and refilled with argon before dry DMF (0.08 M) was added via syringe. The reaction mixture was stirred under an argon atmosphere at 100 ºC overnight. Then, the mixture was cooled to rt, diluted with water and extracted with EtOAc. The combined organic layers were washed with brine several times, dried over anhydrous $MgSO_4$ and concentrated under reduced pressure. The crude was purified by flash column chromatography on silica gel to afford isonitrile **1**.

[3] Si, Y.X.; Zhu, P.F.; Zhang, S.L. Synthesis of Isocyanides by Reacting Primary Amines with Difluorocarbene. *Org. Lett.* **2020**, *22*, 9086–9090.

2.2. General Procedure B for the Synthesis of Isonitriles **1** from Primary Amines (**GP-B**)

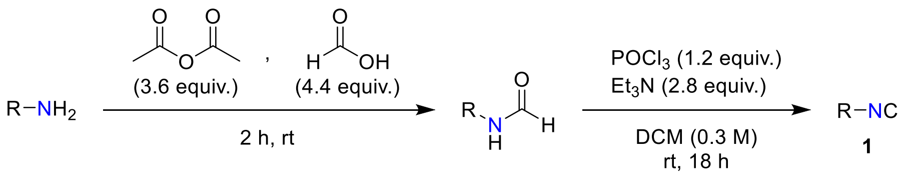

According to a literature procedure,[4] acetic anhydride (3.6 equiv.) and formic acid (4.4 equiv.) were stirred for 2 h at 55 °C to prepare acetic formic anhydride. The corresponding acetic formic anhydride was dropwise added to a stirred solution of the amine at 0 ºC and stirred for 15 min before being warmed to room temperature. Then, the reaction mixture was stirred until full conversion of the primary amine (as judged by TLC analysis) and the volatiles were removed under reduced pressure.

The obtained formamide was dissolved in dry DCM (0.5 M) under argon and cooled to 0 ºC. Triethylamine (4.8 equiv.) and phosphoryl trichloride (1.24 equiv.) were subsequently carefully added and the progress of the reaction was followed by TLC analysis. Thus, the reaction mixture was quenched with a saturated solution of $NaHCO_3$ at 0 ºC and let warm to room temperature for 30 min, before being extracted two times with DCM. The combined organic phases were washed with brine and dried over Na_2SO_4. The solvents were removed under reduced pressure and the crude isonitrile **1** was purified by silica gel chromatography.

[4] Quirós, I.; Martín, M.; Gomez-Mendoza, M.; Cabrera-Afonso, M. J.; Liras, M.; Fernández, I.; Nóvoa, L.; Tortosa, M. *Angew. Chem. Int. Ed.* **2024**, *63*, e202317683.

2.3. Characterization Data for Isonitriles **1**

Methyl 3-(4-isocyanopiperidine-1-carbonyl)bicyclo[1.1.1]pentane-1-carboxylate (1e)

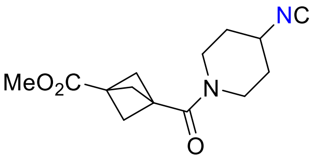

Prepared following **GP-B** from amine **SI-2** (500 mg, 1.98 mmol), the title compound was obtained in 40% yield (207 mg, 0.79 mmol) as a yellow oil after purification by flash column chromatography (SiO_2; 30-60% EtOAc in cyclohexane). $\mathbf{R_f}$ = 0.45 (50% EtOAc in cyclohexane).

^{1}H-NMR (500 MHz, $CDCl_3$) δ 3.93-3.87 (m, 1H), 3.75-3.69 (m, 2H), 3.67 (s, 3H), 3.65-3.52 (m, 2H), 2.35 (s, 6H), 1.89-1.76 (m, 4H). Spectrum

^{13}C-NMR (126 MHz, $CDCl_3$) δ 169.5, 167.0, 156.9 (t, J = 4.3 Hz, -N**C**), 53.8, 51.7, 49.2, 49.1 (t, J = 6.4 Hz, -N**C**), 41.6, 39.5, 38.4, 38.3, 31.8, 30.9. Spectrum

HRMS (ESI+): calculated for $C_{14}H_{18}N_2NaO_3$ $[M+Na]^+$: 285.1210; found: 285.1207

***tert*-Butyl 3-(2-isocyanoethyl)-1*H*-indole-1-carboxylate (1i)**

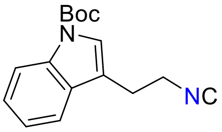

Prepared following **GP-B** from *tert*-butyl 3-(2-aminoethyl)-1*H*-indole-1-carboxylate (1.22 g, 4.72 mmol), the title compound was obtained in 47% (603 mg, 2.23 mmol) yield as a colourless oil after purification by flash column chromatography (SiO_2; 0-15% EtOAc in cyclohexane). $\mathbf{R_f}$ = 0.68 (40% EtOAc in cyclohexane).

^{1}H-NMR (300 MHz, $CDCl_3$) δ 8.11 (d, J = 8.2 Hz, 1H), 7.41 (d, J = 7.8 Hz, 1H), 7.28 (t, J = 7.2 Hz, 1H), 7.19 (t, J = 7.2 Hz, 1H), 3.60 (t, J = 7.1 Hz, 2H), 3.01 (t, J = 7.2 Hz, 2H), 1.61 (s, 9H). Spectrum

Spectroscopic data are in agreement with those described in the literature.[5]

[5] Liu, H.; Dömling, A. *J. Org. Chem.* **2009**, *74*, 6895-6898

Methyl 4-(isocyanomethyl)benzoate (1l)

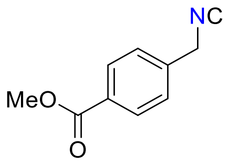

Prepared following **GP-B**, the title compound (531.4 mg, 3.03 mmol) was obtained in 69% yield as a yellow oil, after purification by flash column chromatography (SiO_2; 0 – 20% EtOAc in cyclohexane). R_f = 0.25 (4:1 cyclohexane/EtOAc).

^{1}H NMR (300 MHz, $CDCl_3$): δ 7.97 (d, J = 8.6 Hz, 2H), 7.34 (d, J = 8.7 Hz, 2H), 4.65 (s, 2H), 3.84 (s, 3H). Spectrum

^{13}C NMR (75 MHz, $CDCl_3$): δ 166.2, 158.5 (t, J = 4.9 Hz, -NC), 137.0, 130.04, 129.98, 126.3, 52.1, 45.1 (t, J = 7.8 Hz, C-NC). Spectrum

HRMS (ESI+): calculated for $C_{10}H_9NNaO_2$ [M+Na]: 198.0525; found: 198.0523.

Methyl 4-isocyanobicyclo[2.2.2]octane-1-carboxylate (1n)

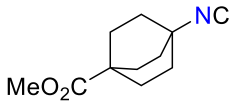

Prepared following **GP-B** from amine **SI-4** (230 mg, 1.26 mmol), the title compound was obtained in 28% yield (68.2 mg, 0.35 mmol) as a yellow oil after purification by flash column chromatography (SiO_2; 0-30% EtOAc in cyclohexane). $\mathbf{R_f}$ = 0.36 (20% EtOAc in cyclohexane).

^{1}H-NMR (500 MHz, $CDCl_3$) δ 3.64 (s, 3H), 1.99-1.93 (m, 6H), 1.89 (dd, J = 10.6, 5.0 Hz, 6H). Spectrum

^{13}C-NMR (126 MHz, $CDCl_3$) δ 176.7, 154.7 (t, J = 5.7 Hz, -N**C**), 52.8 (t, J = 7.0 Hz, -N**C**), 52.1, 37.8, 32.3, 28.1. Spectrum

HRMS (ESI+): calculated for $C_{11}H_{15}NNaO_2$ $[M+Na]^+$: 216.0995; found: 216.0993.

2-((4-Isocyano-4-methylpentyl)oxy)-1,4-dimethylbenzene (1o)

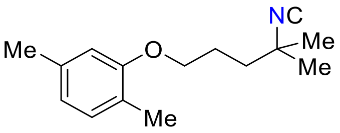

Prepared following **GP-A** from amine **SI-6** (266 mg, 1.20 mmol), the title compound was obtained in 56% yield (154.7 mg, 0.67 mmol) as a yellow solid, after purification by flash column chromatography (SiO_2; 0-5% EtOAc in cyclohexane). $\mathbf{R_f}$ = 0.59 (10% EtOAc in cyclohexane).

^{1}H-NMR (300 MHz, $CDCl_3$): δ 7.01 (d, *J* = 7.4 Hz, 1H), 6.67 (d, *J* = 7.5 Hz, 1H), 6.62 (s, 1H), 3.99 (t, *J* = 5.9 Hz, 2H), 2.31 (s, 3H), 2.18 (s, 3H), 2.05-1.92 (m, 2H), 1.86-1.75 (m, 2H), 1.48-1.43 (m, 6H). Spectrum

^{13}C-NMR (75 MHz, $CDCl_3$): δ 156.8, 153.7 (t, *J* = 4.4 Hz, -N**C**), 136.5, 130.4, 123.5, 120.9, 112.0, 67.2, 57.2 (t, *J* = 5.0 Hz, **C**-NC), 39.2, 29.0, 24.6, 21.4, 15.8. Spectrum

HRMS (ESI+): calculated for $C_{15}H_{21}NO$ $[M]^+$: 231.1618; found: 231.1619.

m.p.: 39 – 40 °C.

***tert*-Butyl (*S*)-2-(isocyanomethyl)pyrrolidine-1-carboxylate (1p)**

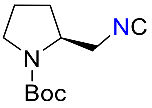

Prepared following **GP-B**, the title compound (86.9 mg, 0.41 mmol) was obtained in 55% yield as a yellow oil after purification by flash column chromatography (SiO_2; 0-10% EtOAc in cyclohexane). $\mathbf{R_f}$ = 0.30 (20% EtOAc in cyclohexane).

^{1}H-NMR (300 MHz, $CDCl_3$): δ 3.88 (br s, 1H), 3.66-3.31 (m, 4H), 2.08-1.75 (m, 4H), 1.41 (s, 9H). Spectrum

Spectroscopic data are in agreement with those described in the literature.[6]

[6] Chennakrishnareddy, G.; Nagendra, G.; Hemantha, H. P.; Das, U.; Guru Row, T. N.; Sureshbabu, V. V. *Tetrahedron* **2010**, *66*, 6718 – 6724.

3. Deaminative Alkylation

3.1. Reaction Setup

All photoredox reactions were performed with a Kessil PR160L-blue LED lamp (max 45 W High Luminous DEX 2100 LED, λ_{max} = 440 nm). The lamp was placed 4.0 cm away from the reaction vials. A typical reaction setup is shown below (Figure S2). Photoredox-catalyzed reactions were performed using 4 mL Screw Neck Vial (clear glass, 45 x 14.7 mm) with screw cap 13 mm black Sil/PTFE septa.

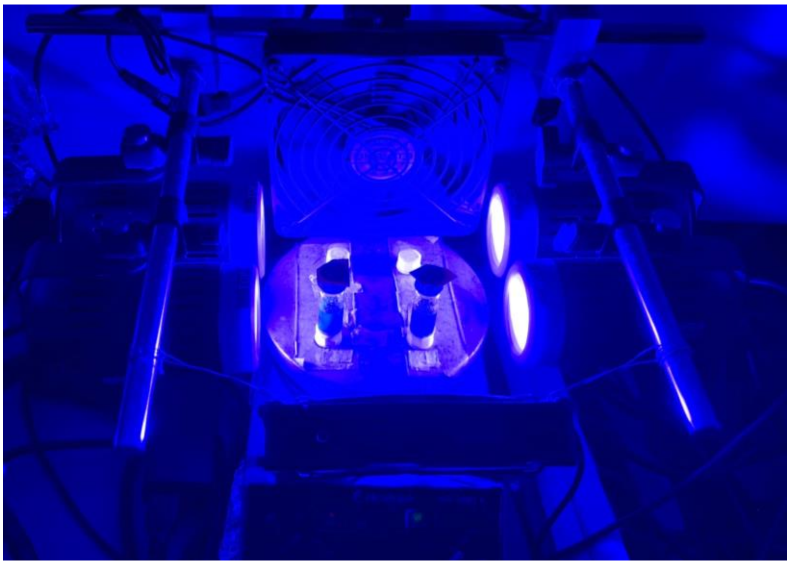

Figure S2.Reaction setup.

3.2. Reaction Optimization

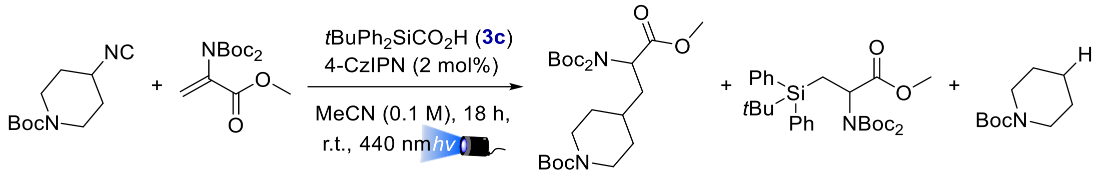

1a **2a** **4aa** **5c** **6a**

Table 1. Reagent Equivalents Screening[a]

Entry	1 (equiv.)	2a (equiv.)	3c (equiv.)	conv.[b] /yield[c] (%)	4aa:6a[b]
**1**	1	1.5	1.2	61/nd	1:0
**2**	1	1	2	66/nd	1:0.1
**3**	1	2	2	64/nd	1:0
**4**	1	2	3	~100/88	1:0.1

[a]Optimization reactions were performed using isonitrile ***1*** *(0.05 mmol), Giese acceptor* ***2a****, tert-butyldiphenylsilacarboxylic acid* ***3c****, and 4-CzIPN (1.00 µmol) in dry degassed acetonitrile (0.5 mL, c = 0.1 M) under blue LEDs irradiation (λ_{max} = 440 nm) for 18 h at rt; [b]Determined by ^{1}H-NMR; [c]Determined after isolation by silica gel flash column chromatography; nd = not determined.*

For the reaction evaluation and subsequent optimization process, we decided to employ isonitrile **1a** as the limiting reagent because it represents the valuable reaction component of the developed reaction. We believe that the use of an excess of isonitrile would limit the synthetic utility of the method, especially when starting from complex primary amines as isonitrile precursors or in late-stage functionalization.

We observed that to achieve full conversion while minimizing hydrodeamination product **6a**, it was necessary to employ an excess (3 equiv.) of silacarboxylic acid **3c** and an excess of Giese acceptor **2a**. The excess of Giese acceptor reacts with silacarboxylic acid **3c** to form product **5c** that was easily separated by flash chromatography on silica gel during the purification process.

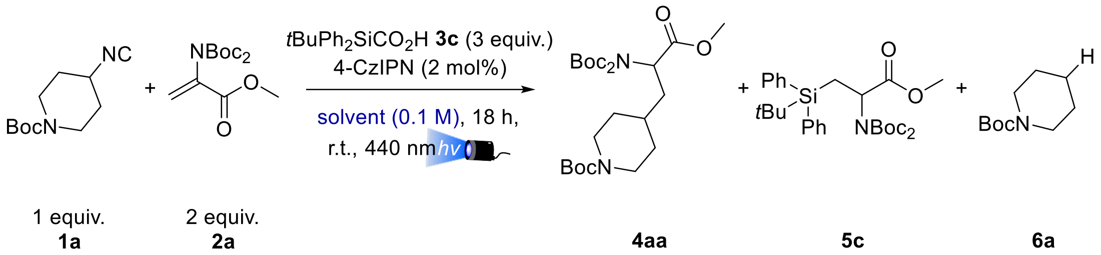

Table 2. Solvent Screening

Entry	Solvent	conv. [a] /yield [b] (%)	4aa:6a [a]
**1**	MeCN	~100/88	1:0.1
**2**	THF	~100/91	1:0
**3**	Acetone	~100/82	1:0.1
**4**	DCM	byproducts	-

*[a]Optimization reactions were performed using isonitrile **1** (0.05 mmol), Giese acceptor **2a** (0.10 mmol), tert-butyldiphenylsilacarboxylic acid **3c** (0.15 mmol), and 4-CzIPN (1.00 μmol) in dry degassed solvent (0.5 mL, c = 0.1 M) under blue LEDs irradiation (λ_{max} = 440 nm) for 18 h at rt; [b]Determined by ^{1}H-NMR; [c]Determined after isolation by silica gel flash column chromatography.*

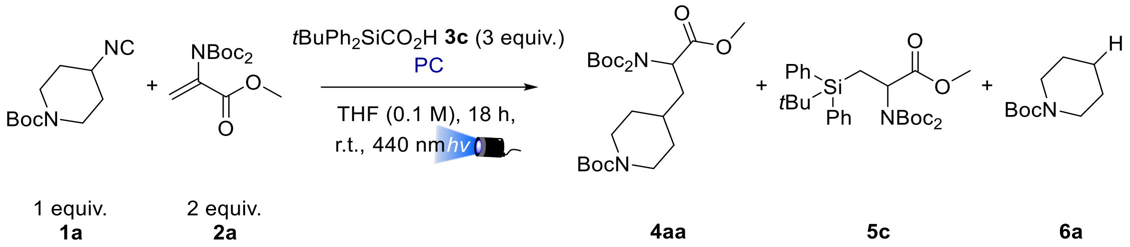

Table 3. Photocatalyst Screening[a]

Entry	PC (mol%)	conv.[b]/yield[c] (%)	4aa:6a[b]
**1**	4-CzIPN (2 mol%)	~100/91	1:0
**2**	4-CzIPN (1 mol%)	~100/79	1:0
**3**	[Ir{dFCF$_3$ppy}$_2$(bpy)]PF$_6$ (2 mol%)	~100/45	1:0.6

*[a]Optimization reactions were performed using isonitrile **1** (0.05 mmol), Giese acceptor **2a** (0.10 mmol), and tert-butyldiphenylsilacarboxylic acid **3c** (0.15 mmol) and the corresponding photoredox catalyst in dry degassed THF (0.5 ml, c = 0.1 M) under blue LEDs irradiation (λ_{max} = 440 nm) for 18 h at rt; [b]Determined by ^{1}H-NMR; [c]Determined after isolation by silica gel flash column chromatography.*

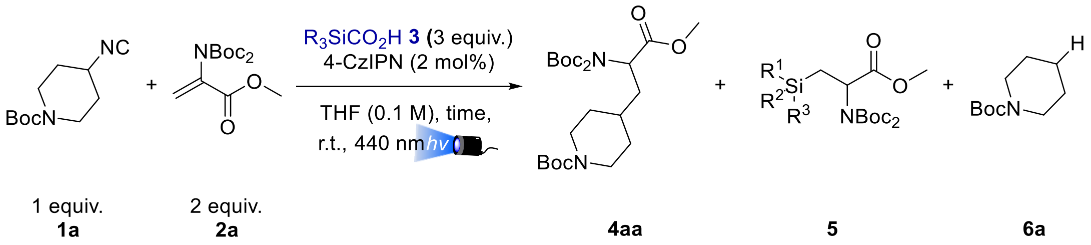

Table 4. Silacarboxylic Acid Screening and reaction time[a]

Entry	3	R^1	R^2	R^3	time	conv.[b] /yield[c] (%)	4aa:6a[c]
**1**	c	*t*Bu	Ph	Ph	18 h	~100/91	1:0
**2**	d	Me	Ph	Ph	18 h	~100/85	1:0.1
**3**	e	Me	Me	Ph	18 h	~100/72	1:0.4
**4**	f	TMS	TMS	TMS	18 h	byproducts	-
**5**	c	*t*Bu	Ph	Ph	2 h	~100/91	1:0

*[a]Optimization reactions were performed using isonitrile **1** (0.05 mmol), Giese acceptor **2a** (0.10 mmol), the corresponding silacarboxylic acid **3** (0.15 mmol) and 4-CzIPN (1.00 µmol) in dry degassed THF (0.5 ml, c = 0.1 M) under blue LEDs irradiation (λ_{max} = 440 nm) for 18 h at rt; [b]Determined by ^{1}H-NMR; [c]Determined after isolation by silica gel flash column chromatography.*

3.3. General Procedure for the Deaminative Alkylation (**GP-C**):

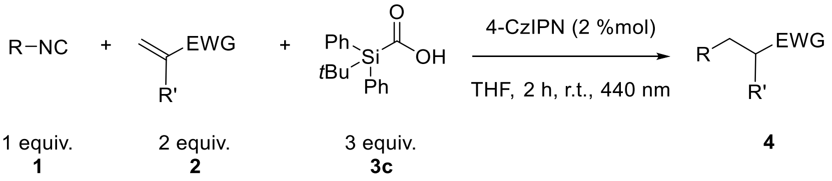

An oven-dried vial equipped with a magnetic stirring bar was charged with the corresponding isonitrile (**1**, 0.10 mmol, 1 equiv.), the electron-deficient olefin (**2**, 0.200 mmol, 2 equiv.), *tert*-butyldiphenylsilacarboxylic acid (**3a**, 85.3 mg, 0.300 mmol, 3 equiv.) and 4-CzIPN (1.6 mg, 2.00 µmol, 2 mol%). The vial was sealed with a septum cap, before being evacuated *in vacuum* and refilled with argon (3x). Degassed anhydrous THF was added (1 ml, 0.1 M) by syringe and the reaction mixture was irradiated with blue LEDs lamp (λ_{max} = 440 nm) at room temperature for 2 hours. Afterwards, solvent was removed *in vacuo* and the residue was purified by silica gel chromatography.

3.4. Characterization Data for Alkylation Products

tert-Butyl 4-(2-(bis(_tert_-butoxycarbonyl)amino)-3-methoxy-3-oxopropyl)piperidine-1-carboxylate (4aa)

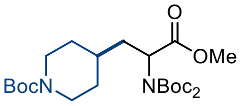

Synthetised by **GP-C** using *tert*-butyl 4-(isocyanomethyl) piperidine-1-carboxylate (**1a**, 21.0 mg, 0.100 mmol, 1 equiv.) and methyl 2-(bis(*tert*-butoxycarbonyl)amino)acrylate (**2a**, 60.3 mg, 0.200 mmol, 2 equiv.). The crude product was purified by flash column chromatography (SiO_2; 5-20 % diethyl ether in pentane) affording 44.3 mg of product in 91% yield as a colourless oil. $\mathbf{R_f}$ = 0.40 (20% EtOAc in cyclohexane).

^{1}H-NMR (300 MHz, $CDCl_3$) δ 4.94 (dd, *J* = 9.4, 5.1 Hz, 1H), 4.05 (br d, *J* = 13.2 Hz, 2H), 3.69 (s, 3H), 2.64 (br q, *J* = 12.3 Hz, 2H), 2.05-1.96 (m, 1H), 1.86-1.74 (m, 2H), 1.61-1.56 (m, 1H), 1.48 (s, 18H), 1.43 (s, 9H), 1.26-0.98 (m, 3H). Spectrum

Spectroscopical data are in agreement with those reported in the literature.[7]

Methyl 2-(bis(_tert_-butoxycarbonyl)amino)-3-(tetrahydro-2H-pyran-4-yl)propanoate (4ba)

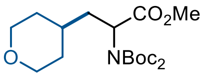

Synthetised by **GP-C** using 4-isocyanotetrahydro-2*H*-pyran (**1b**, 11.1 mg, 0.100 mmol, 1 equiv.) and methyl 2-(bis(*tert*-butoxycarbonyl)amino)acrylate (**2a**, 60.3 mg, 0.200 mmol, 2 equiv.). The crude product was purified by flash column chromatography (SiO_2; 10-30% diethyl ether in pentane), affording 32.7 mg of product in 96% yield as a yellow oil. $\mathbf{R_f}$ = 0.30 (20% EtOAc in cyclohexane).

^{1}H-NMR (300 MHz, $CDCl_3$) δ 4.95 (dd, *J*= 9.4, 5.0 Hz, 1H), 4.02-3.87 (m, 2H), 3.70 (s, 3H), 3.34 (qd, *J* = 11.5, 2.2 Hz, 2H), 2.03 (ddd, *J* = 14.3, 8.3, 5.1 Hz, 1H), 1.83 (ddd, *J* = 14.3, 9.4, 4.8 Hz, 1H), 1.77-1.63 (m, 2H), 1.49 (m, 19H), 1.41-1.18 (m, 2H). Spectrum

Spectroscopical data are in agreement with those reported in the literature.[8]

[7] Delgado, J. A. C.; Correia, J. T. M.; Pissinati, E. F.; Paixão, M. W. *Org. Lett.* **2021**, *23*, 5251-5255.

[8] Ranjan, P.; Pillitteri, S.; Coppola, G.; Oliva, M.; Van der Eycken, E. V.; Sharma, U. K.; *ACS Catal.* **2021**, *11*, 10862-10870.

Methyl 2-(bis(*tert*-butoxycarbonyl)amino)-3-(1,1-dioxidotetrahydro-2H-thiopyran-4-yl)propanoate (4ca)

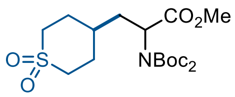

Synthetised by **GP-C** using 4-isocyanotetrahydro-2*H*-thiopyran 1,1-dioxide (**1c**, 15.9 mg, 0.100 mmol, 1 equiv.) and methyl 2-(bis(*tert*-butoxycarbonyl)amino)acrylate (**2a**, 60.3 mg, 0.200 mmol, 2 equiv.). The crude product was purified by flash column chromatography (SiO_2; 40-70% diethyl ether in pentane), affording 41.7 mg of product in 96% yield as ra yellow oil. **R_f**= 0.30 (40% EtOAc in cyclohexane).

^{1}H-NMR (500 MHz, $CDCl_3$) δ 4.89 (dd, *J* = 8.7, 5.4 Hz, 1H), 3.71 (s, 3H), 3.07-2.99 (m, 2H), 2.99-2.84 (m, 2H), 2.27-2.19 (m, 1H), 2.19-2.12 (m, 1H), 2.10-2.02 (m, 1H), 1.97-1.87 (m, 1H), 1.87-1.80 (m, 2H), 1.66-1.53 (m, 1H), 1.49 (s, 18H). Spectrum

^{13}C-NMR (126 MHz, $CDCl_3$) δ 171.1, 152.3, 83.7, 55.9, 52.5, 51.0, 50.9, 35.7, 32.7, 30.5, 29.5, 28.1. Spectrum

HRMS (ESI+): calculated for $C_{19}H_{33}NNaO_8S$ $[M+Na]^+$: 458.1819; found: 458.1809.

***tert*-Butyl 3-(2-(bis(*tert*-butoxycarbonyl)amino)-3-methoxy-3-oxopropyl)azetidine-1-carboxylate (4da)**

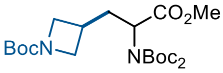

Synthetised by **GP-C** using *tert*-butyl 3-isocyanoazetidine-1-carboxylate (**1d**, 18.2 mg, 0.100 mmol, 1 equiv.) and methyl 2-(bis(*tert*-butoxycarbonyl)amino)acrylate (**2a**, 60.3 mg, 0.200 mmol, 2 equiv.). The crude product was purified by flash column chromatography (SiO_2; 5-20 % diethyl ether in pentane) affording 43.4 mg of product in 95% yield as a yellow oil. **R_f** = 0.41 (20% EtOAc in cyclohexane).

^{1}H-NMR (300 MHz, $CDCl_3$) δ 4.81 (dd, *J* = 9.5, 9.3 Hz, 1H), 4.03-3.91 (m, 2H), 3.70 (s, 3H), 3.62-3.51 (m, 2H), 2.63-2.47 (m, 1H), 2.44-2.29 (m, 1H), 2.29-2.10 (m, 1H), 1.49 (s, 18H), 1.44 (s, 9H). Spectrum

Spectroscopical data are in agreement with those reported in the literature.[9]

---

[9] Constantin, T.; Zanini, M.; Regni, A.; Sheikh, N. S.; Juliá, F.; Leonori, D. *Science* **2020**, *367*, 1021-1026.

Methyl 3-(4-(2-(bis(*tert*-butoxycarbonyl)amino)-3-methoxy-3-oxopropyl)piperidine-1-carbonyl)bicyclo[1.1.1]pentane-1-carboxylate (4ea)

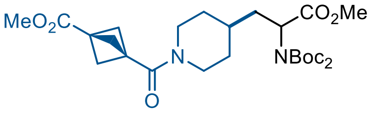

Synthetised by **GP-C** using methyl 3-(4-isocyanopiperidine-1-carbonyl)bicyclo[1.1.1]pentane-1-carboxylate (**1e**, 26.2 mg, 0.100 mmol, 1 equiv.) and methyl 2-(bis(*tert*-butoxycarbonyl)amino)acrylate (**2a**, 60.3 mg, 0.200 mmol, 2 equiv.). The crude product was purified by two flash column chromatography (SiO_2; 30-60% diethyl ether in pentane and then 0-20% EtOAc in DCM), affording 53.9 mg of product in 93% yield as a yellow oil. $\mathbf{R_f}$ = 0.50 (40% EtOAc in cyclohexane).

^{1}H-NMR (500 MHz, $CDCl_3$) δ 4.93 (dd, *J* = 9.2, 5.1 Hz, 1H), 4.51 (br d, *J* = 13.3 Hz, 1H), 4.05 (br d, *J* = 13.4 Hz, 1H), 3.70 (s, 3H), 3.68 (s, 3H), 3.03-2.90 (m, 1H), 2.59-2.46 (m, 1H), 2.40-2.32 (m, 6H), 2.08-1.99 (m, 1H), 1.92-1.77 (m, 2H), 1.77-1.63 (m, 2H), 1.49 (s, 18H), 1.21-1.02 (m, 2H). Spectrum

^{13}C-NMR (126 MHz, $CDCl_3$) δ 171.5, 170.0, 167.1, 152.3, 152.2, 83.5, 55.7, 54.1, 52.4, 51.9, 45.8 and 45.6 (same C: rotamers), 42.6 and 42.5 (same C, rotamers), 40.0, 38.8, 36.8, 33.2, 32.5 and 32.3 (same C: rotamers), 31.4, 28.1. Spectrum

HRMS (ESI+): calculated for $C_{27}H_{42}N_2NaO_9$ $[M+Na]^+$: 561.2783; found: 561.2773.

***tert*-Butyl 4-(3-(bis(*tert*-butoxycarbonyl)amino)-4-methoxy-4-oxobutyl)piperidine-1-carboxylate (4fa)**

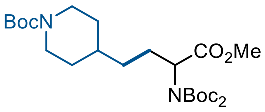

Synthetised by **GP-C** using *tert*-butyl 4-(isocyanomethyl)piperidine-1-carboxylate (**1f**, 22.4 mg, 0.100 mmol, 1 equiv.) and methyl 2-(bis(*tert*-butoxycarbonyl)amino)acrylate (**2a**, 60.3 mg, 0.200 mmol, 2 equiv.). The crude product was purified by flash column chromatography (SiO_2; 5-20 % diethyl ether in pentane) affording 32.2 mg of product in 64% yield as a colourless oil. $\mathbf{R_f}$ = 0.45 (20% EtOAc in cyclohexane).

^{1}H-NMR (500 MHz, $CDCl_3$) δ 4.81 (dd, *J* = 9.6, 5.1 Hz, 1H), 4.05 (br d, *J* = 13.2 Hz, 2H), 3.69 (s, 3H), 2.65 (br t, *J* = 12.7 Hz, 2H), 2.17-2.05 (m, 1H), 1.95-1.82 (m, 1H), 1.69-1.62 (m, 2H), 1.48 (s, 18H), 1.43 (s, 9H), 1.40-1.27 (m, 3H), 1.15-0.97 (m, 2H). Spectrum

^{13}C-NMR (75 MHz, $CDCl_3$) δ 171.5, 155.0, 152.3, 83.2, 79.3, 58.3, 52.3, 35.8, 34.9, 33.1, 32.0, 28.6, 28.1, 27.1. Spectrum

HRMS (ESI+): calculated for $C_{25}H_{44}N_2NaO_8$ $[M+Na]^+$: 523.2990; found: 523.2979.

Methyl 2-(bis(*tert*-butoxycarbonyl)amino)-5-phenoxypentanoate (4ga)

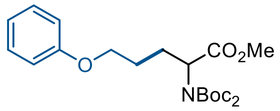

Synthetised by **GP-C** using (2-isocyanoethoxy)benzene (**1g**, 14.7 mg, 0.100 mmol, 1 equiv.) and methyl 2-(bis(*tert*-butoxycarbonyl)amino)acrylate (**2a**, 60.3 mg, 0.200 mmol, 2 equiv.). The crude product was purified by flash column chromatography (SiO_2; 5-20 % diethyl ether in pentane) affording 44.3 mg of product in 79% yield as a colourless oil. $\mathbf{R_f}$ = 0.50 (20% EtOAc in cyclohexane).

^{1}H-NMR (300 MHz, $CDCl_3$) δ 7.32-7.18 (m, 2H), 6.96-6.87 (m, 3H), 4.93 (dd, *J* = 9.6, 5.2 Hz, 1H), 3.98 (t, *J* = 6.2 Hz, 2H), 3.72 (s, 3H), 2.42-2.27 (m, 1H), 2.15-2.00 (m, 1H), 1.93-1.78 (m, 2H), 1.50 (s, 18H). Spectrum

^{13}C-NMR (75 MHz, $CDCl_3$) δ 171.4, 159.1, 152.3, 129.6, 120.8, 114.7, 83.3, 67.3, 58.0, 52.3, 28.2, 26.8, 26.3. Spectrum

HRMS (ESI+): calculated for $C_{22}H_{33}NNaO_7$ $[M+Na]^+$: 446.2149; found: 446.2137.

Methyl 5-(1-benzyl-1H-indol-3-yl)-2-(bis(*tert*-butoxycarbonyl)amino)pentanoate (4ha)

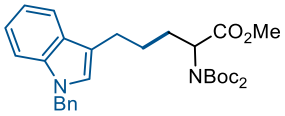

Synthetised by **GP-C** using 1-benzyl-3-(2-isocyanoethyl)-1*H*-indole (**1h**, 26.0 mg, 0.100 mmol, 1 equiv.) and methyl 2-(bis(*tert*-butoxycarbonyl)amino)acrylate (**2a**, 60.3 mg, 0.200 mmol, 2 equiv.). The crude product was purified by flash column chromatography (SiO_2; 5-20% diethyl ether in pentane) affording 20.8 mg of product in 39% yield as a colourless oil. $\mathbf{R_f}$ = 0.45 (20% EtOAc in cyclohexane).

^{1}H-NMR (300 MHz, $CDCl_3$) δ 7.64 (d, *J* = 7.8 Hz, 1H), 7.35-7.26 (m, 4H), 7.21-7.09 (m, 4H), 6.95 (s, 1H), 5.30 (s, 2H), 4.97 (dd, *J* = 9.5, 5.2 Hz, 1H), 3.74 (s, 3H), 2.91-2.75 (m, 2H), 2.32-2.20 (m, 1H), 2.09-1.97 (m, 1H), 1.87-1.76 (m, 2H), 1.49 (s, 18H). Spectrum

^{13}C-NMR (75 MHz, $CDCl_3$) δ 171.6, 152.3, 137.9, 136.9, 128.8, 128.3, 127.6, 126.9, 125.6, 121.7, 119.3, 118.9, 115.6, 109.7, 83.1, 58.1, 52.2, 50.0, 29.9, 28.1, 26.9, 24.9. Spectrum

HRMS (ESI+): calculated for $C_{31}H_{40}N_2NaO_6$ $[M+Na]^+$: 559.2779; found: 559.2763.

Methyl 5-(1-benzyl-1*H*-indol-3-yl)-2-(bis(*tert*-butoxycarbonyl)amino)pentanoate (4ia)

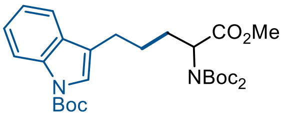

Synthetised by **GP-C** using tert-butyl 3-(2-isocyanoethyl)-1H-indole-1-carboxylate (**1i**, 27.0 mg, 0.100 mmol, 1 equiv.) and methyl 2-(bis(*tert*-butoxycarbonyl)amino)acrylate (**2a**, 60.3 mg, 0.200 mmol, 2 equiv.). The crude product was purified by flash column chromatography (SiO_2; 5-20 % diethyl ether in pentane) affording 20.8 mg of product in 90% yield as a colourless oil. $\mathbf{R_f}$ = 0.4 (20% AcOEt in cyclohexane).

^{1}**H-NMR** (300 MHz, $CDCl_3$) δ 8.09 (d, *J* = 8.2 Hz, 1H), 7.51-7.44 (m, 1H), 7.40-7.13 (m, 3H), 4.90 (dd, *J* = 9.5, 5.2 Hz, 1H), 3.68 (s, 3H), 2.83-2.59 (m, 2H), 2.26-2.13 (m, 1H), 2.04-1.88 (m, 1H), 1.82-1.68 (m, 2H), 1.63 (s, 9H), 1.43 (s, 18H). Spectrum

^{13}**C-NMR** (75 MHz, $CDCl_3$) δ 171.5, 152.3, 149.9, 135.8, 130.8, 124.3, 122.5, 122.4, 120.8, 119.1, 115.4, 83.2, 58.0, 52.3, 29.8, 28.4, 28.1, 26.0, 24.7. Spectrum

HRMS (ESI+): calculated for $C_{29}H_{42}N_2NaO_8$ $[M+Na]^+$: 569.2833; found: 569.2814.

Methyl 2-(bis(*tert*-butoxycarbonyl)amino)-5-((1R,4aR)-7-isopropyl-1,4a-dimethyl-1,2,3,4,4a,9,10,10a-octahydrophenanthren-1-yl)pentanoate (4ja)

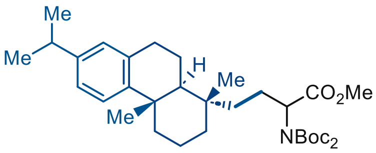

Synthetised by **GP-C** using (1*R*,4a*S*)-1-(isocyanomethyl)-7-isopropyl-1,4a-dimethyl-1,2,3,4,4a,9,10,10a-octahydrophenanthrene (**1j**, 26.0 mg, 0.100 mmol, 1 equiv.) and methyl 2-(bis(*tert*-butoxycarbonyl)amino)acrylate (**2a**, 60.3 mg, 0.200 mmol, 2 equiv.). The crude product was purified by two flash column chromatography (SiO_2; 5-20 % diethyl ether in pentane and then 50-70 % DCM in cyclohexane) affording 16.4 mg in 29% yield as a mixture of diastereoisomers (*dr* = 1:1) as a yellow oil. $\mathbf{R_f}$ = 0.59 (20% EtOAc in cyclohexane).

^{1}**H-NMR** (500 MHz, $CDCl_3$) δ 7.17 (d, *J* = 8.3 Hz, 1H), 6.99 (d, *J* = 8.3 Hz, 1H), 6.89 (d, *J* = 1.5, 0.5H), 6.87 (d, *J* = 1.5, 0.5H), 4.79 (m, 1H), 3.70 (s, 3H), 2.84 (m, 3H), 2.27 (d, *J* = 12.9 Hz, 1H), 2.09-1.94 (m, 2H), 1.88-1.78 (m, 2H,) 1.78-1.57 (m, 4H), 1.50 (s, 9H), 1.49 (s, 9H), 1.47-1.29 (m, 6H), 1.26 (br s, 3H), 1.23 (d, *J* = 6.9 Hz, 3H), 1.22 (d, *J* = 6.9 Hz, 3H), 0.94 (s, 1.5H), 0.91 (s, 1.5H). Spectrum

^{13}**C-NMR** (75 MHz, $CDCl_3$) δ 171.5, 152.2.3, 152.1, 147.8, 145.6, 135.0, 134.9, 126.95, 126.91, 124.5, 124.0, 83.11, 83.08, 59.1, 59.0, 52.2, 47.7, 47.5, 40.4, 38.7, 37.72, 37.68, 37.2, 35.7, 33.6, 30.5, 30.4, 29.8, 28.1, 25.46, 25.42, 24.1, 24.0, 23.9, 21.1, 20.9, 19.1, 18.9, 18.8. Spectrum

HRMS (ESI+): calculated for $C_{34}H_{53}NNaO_6$ $[M+Na]^+$: 594.3765; found: 594.3750.

Methyl 2-(bis(*tert*-butoxycarbonyl)amino)-4-(4-methoxyphenyl)butanoate (4ka)

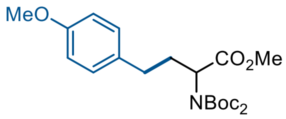

Synthetised by a slighlty modified **GP-C** using 1-(isocyanomethyl)-4-methoxybenzene (**1k**, 14.7 mg, 0.100 mmol, 1 equiv,), methyl 2-(bis(*tert*-butoxycarbonyl)amino)acrylate (**2a**, 60.3 mg, 0.200 mmol, 2 equiv.) and *tert*-butyldiphenylsilacarboxylic acid (114 mg, 0.400 mmol, 4 equiv,). The reaction mixture was irradiated for 4 h. The crude product was purified by flash column chromatography (SiO_2; 5-20 % diethyl ether in pentane) affording 36.9 mg of product in 87% yield as a pale yellow solid. $\mathbf{R_f}$ = 0.58 (20% EtOAc in cyclohexane).

^{1}**H-NMR** (300 MHz, $CDCl_3$) δ 7.17-7.04 (m, 2H), 6.89-6.77 (m, 2H), 4.89 (dd, J = 9.3, 5.2 Hz, 1H), 3.78 (s, 3H), 3.71 (s, 3H), 2.62 (t, J = 8.1 Hz, 2H), 2.50-2.35 (m, 1H), 2.24-2.07 (m, 1H), 1.49 (s, 18H). Spectrum

^{13}**C-NMR** (75 MHz, $CDCl_3$) δ 171.4, 158.1, 152.3, 133.4, 129.4, 114.0, 83.2, 57.9, 55.4, 52.3, 32.1, 31.8, 28.1. Spectrum

HRMS (ESI+): calculated for $C_{22}H_{33}NNaO_7$ $[M+Na]^+$: 446.2149; found: 446.2142.

m.p.: 100-101 ºC

***tert*-Butyl 4-(2-(bis(*tert*-butoxycarbonyl)amino)-3-methoxy-3-oxopropyl)-4-methylpiperidine-1-carboxylate (4ma)**

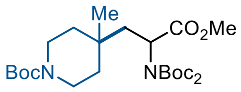

Synthetised by **GP-C** using *tert*-butyl 4-isocyano-4-methylpiperidine-1-carboxylate (**1m**, 22.4 mg, 0.100 mmol, 1 equiv.) and methyl 2-(bis(*tert*-butoxycarbonyl)amino)acrylate (**2a**, 60.3 mg, 0.200 mmol, 2 equiv.). The crude product was purified by flash column chromatography (SiO_2; 5-20 % diethyl ether in pentane) affording 46.5 mg of product in 93% yield as a colourless oil. $\mathbf{R_f}$= 0.48 (20% EtOAc in cyclohexane).

^{1}H-NMR (300 MHz, $CDCl_3$) δ 4.94 (dd, *J* = 7.5, 3.2 Hz, 1H), 3.69 (s, 3H), 3.61-3.46 (m, 2H), 3.26-3.11 (m, 2H), 2.30 (dd, *J* = 15.3, 3.3 Hz, 1H), 1.74 (dd, *J* = 15.3, 7.5 Hz, 1H), 1.48 (s, 18H), 1.43 (s, 9H), 1.38-1.27 (m, 4H), 0.96 (s, 3H). Spectrum

Spectroscopical data are in agreement with those reported in the literature.[10]

Methyl 4-(2-(bis(*tert*-butoxycarbonyl)amino)-3-methoxy-3-oxopropyl)bicyclo[2.2.2]octane-1-carboxylate (4na)

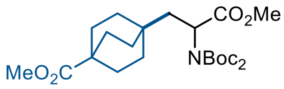

Synthetised by **GP-C** using methyl 4-isocyanobicyclo[2.2.2]octane-1-carboxylate (**1n**, 19.3 mg, 0.100 mmol, 1 equiv.) and methyl 2-(bis(*tert*-butoxycarbonyl)amino)acrylate (**2a**, 60.3 mg, 0.200 mmol, 2 equiv.). The crude product was purified by flash column chromatography (SiO_2; 10-25% diethyl ether in pentane) affording 37.7 mg of product in 80% yield as a colourless oil. $\mathbf{R_f}$ = 0.50 (20% EtOAc in cyclohexane).

^{1}H-NMR (300 MHz, $CDCl_3$) δ 4.91 (dd, *J* = 7.7, 3.5 Hz, 1H), 3.68 (s, 3H), 3.62 (s, 3H), 2.15 (dd, *J* = 15.3, 3.5 Hz, 1H), 1.75 (t, *J* = 7.8 Hz, 6H), 1.61 (dd, *J* = 15.3, 7.8 Hz, 1H), 1.49 (s, 18H), 1.47-1.38 (m, 6H). Spectrum

Spectroscopical data are in agreement with those reported in the literature.[13]

Methyl 2-(bis(*tert*-butoxycarbonyl)amino)-7-(2,5-dimethylphenoxy)-4,4-dimethylheptanoate (4oa)

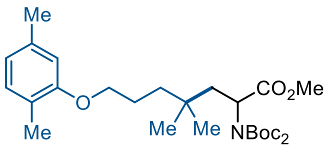

Synthetised by **GP-C** using methyl 2-((4-isocyano-4-methylpentyl)oxy)-1,4-dimethylbenzene (**1o**, 23.1 mg, 0.100 mmol, 1 equiv.) and methyl 2-(bis(*tert*-butoxycarbonyl)amino)acrylate (**2a**, 60.3 mg, 0.200 mmol, 2 equiv.). The crude product was purified by two flash column chromatography (SiO_2; 0-10% EtOAc in cyclohexane and then 5-10% diethyl ether in pentane) affording 45.2 mg of product in 89% yield as a colourless oil. $\mathbf{R_f}$ = 0.60 (20% diethyl ether in pentane).

---

[10]Ashley, M. A.; Rovis, T. *J. Am. Chem. Soc.* **2020,** *142*, 18310-18316.

^{1}H-NMR (300 MHz, $CDCl_3$) δ 6.99 (d, *J* = 7.4 Hz, 1H), 6.64 (d, *J* = 7.7 Hz, 1H), 6.60 (s, 1H), 4.96 (dd, *J* = 7.6, 3.1 Hz, 1H), 3.89 (t, *J* = 6.6 Hz, 2H), 3.70 (s, 3H), 2.34-2.24 (m, 4H), 2.17 (s, 3H), 1.81-1.69 (m, 3H), 1.49 (s, 18H), 1.44-1.35 (m, 2H), 0.94 (s, 6H). Spectrum

Spectroscopical data are in agreement with those reported in the literature.[7]

***tert*-Butyl 4-(3-(*tert*-butoxy)-3-oxopropyl)piperidine-1-carboxylate (4ab)**

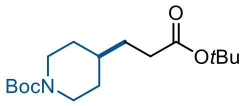

Synthetised by **GP-C** using *tert*-butyl 4-(isocyanomethyl) piperidine-1-carboxylate (**1a**, 21.0 mg, 0.100 mmol, 1 equiv.) and *tert*-butyl acrylate (**2b**, 29.2 µl, 0.200 mmol, 2 equiv.). The crude product was purified by flash column chromatography (SiO_2; 2-15 % AcOEt in cyclohexane), affording 19.2 mg of product in 61% yield as a colourless oil. $\mathbf{R_f}$ = 0.26 (10% diethyl ether in pentane).

^{1}H-NMR (300 MHz, $CDCl_3$) δ 4.07 (br d, *J* = 12.9 Hz, 2H), 2.66 (br t, *J* = 12.9 Hz, 2H), 2.23 (t, *J* = 7.6 Hz, 2H), 1.69-1.60 (m, 2H) 1.60-1.50 (m, 3H), 1.45 (s, 9H), 1.44 (s, 9H), 1.08 (qd, *J* = 12.2, 4.3 Hz, 2H). Spectrum

Spectroscopical data are in agreement with those reported in the literature.[11]

***tert*-Butyl 4-(3-oxo-3-(phenylamino)propyl)piperidine-1-carboxylate (4ac)**

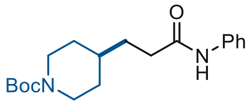

Synthetised by **GP-C** using *tert*-butyl 4-(isocyanomethyl) piperidine-1-carboxylate (**1a**, 21.0 mg, 0.100 mmol, 1 equiv.) and *N*-phenylacrylamide (**2c**, 29.4 mg, 0.200 mmol, 2 equiv.). The crude product was purified by flash column chromatography (SiO_2; 2-15 % AcOEt in cyclohexane) affording 12.2 mg of product in 37% yield as a white solid. $\mathbf{R_f}$ = 0.33 (20% EtOAc in cyclohexane).

^{1}H-NMR (300 MHz, $CDCl_3$) δ 7.51 (d, *J* = 7.9 Hz, 2H), 7.31 (t, *J* = 7.9 Hz, 2H), 7.10 (t, *J* = 6.9 Hz, 1H), 4.08 (br d, *J* = 13.0 Hz, 2H), 2.67 (br t, *J* = 13.0 Hz, 2H), 2.38 (t, *J* = 7.7 Hz, 2H), 1.75-1.60 (m, 4H), 1.50-1.41 (m, 10H), 1.21-1.03 (m, 2H). Spectrum

[11] Liu, Z.; Wei, S.; Liang, A.; Li, J.; Zou, D.; Wu, Y.; Wu, Y.; *Tetrahedron Letters* **2019,** *60* (6), 485-488.

Spectroscopical data are in agreement with those reported in the literature.[12]

***tert*-Butyl 4-(2-(1,3-dioxoisoindolin-2-yl)-3-ethoxy-3-oxopropyl)piperidine-1-carboxylate. (4ad)**

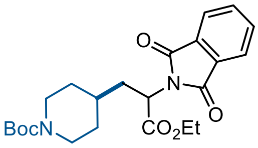

Synthetised by **GP-C** using *tert*-butyl 4-(isocyanomethyl) piperidine-1-carboxylate (**1a**, 21.0 mg, 0.100 mmol, 1 equiv.) and ethyl 2-(1,3-dioxoisoindolin-2-yl)acrylate (**2d**, 49 mg, 0.200 mmol, 2 equiv.). The crude product was purified by flash column chromatography (SiO_2; 5-15 % AcOEt in cyclohexane) affording 31.4 mg of product in 73% yield as a white solid. $\mathbf{R_f}$ = 0.24 (20% EtOAc in cyclohexane).

^{1}H-NMR (500 MHz, Tetrachloroethane-d2, 408 K) δ 7.90-7.79 (m, 2H), 7.76-7.63 (m, 2H), 4.87 (dd, J = 9.8, 5.2 Hz, 1H), 4.23-4.11 (m, 2H), 4.01-3.89 (m, 2H), 2.66 (dddd, J = 13.4, 11.8, 3.2, 1.7 Hz, 2H), 2.22 (ddd, J = 15.0, 9.8, 5.3 Hz, 1H), 2.14 (ddd, J = 14.3, 8.4, 5.2 Hz, 1H)., 1.80-1.73 (m, 1H), 1.64-1.57 (m, 1H), 1.41 (m, 10H), 1.24-1.11 (m, 5H). Spectrum

^{13}C-NMR (126 MHz, Tetrachloroethane-d2, 408 K) δ 169.5, 167.7, 155.0, 134.2, 132.4, 123.6, 79.3, 62.0, 50.8, 44.2, 44.1, 35.6, 33.6, 32.4, 31.5, 28.7, 14.1. Spectrum

HRMS (ESI+): calculated for $C_{23}H_{30}N_2NaO_6$ $[M+Na]^+$: 453.1996; found: 453.1989.

m.p.: 125-126 ºC

Benzyl (2*S*,4*S*)-4-((1-(*tert*-butoxycarbonyl)piperidin-4-yl)methyl)-2-(*tert*-butyl)-5-oxooxazolidine-3-carboxylate (4ae)

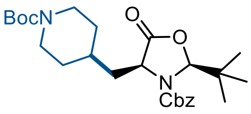

Synthetised by **GP-C** using tert-butyl 4-isocyanopiperidine-1-carboxylate (**1a**, 21.0 mg, 0.10 mmol, 1 equiv.) and benzyl (*S*)-2-(*tert*-butyl)-4-methylene-5-oxooxazolidine-3-carboxylate (**2e**, 57.9 mg, 0.200 mmol, 2 equiv.). The crude product was purified firstly by flash column chromatography (SiO_2; from 10 to 40 % EtOAc in cyclohexane) and then by preparative TLC (30 % diethyl ether in pentane), affording 28.5 mg of product in 60% yield as a yellow oil. The product corresponds to the *cis*-diastereoisomer (*dr* ≥ 95:5). $\mathbf{R_f}$ = 0.41 (20% EtOAc in cyclohexane).

[12] Wu, J.; Grant, P. S. ; Li, X.; Noble, A.; Aggarwal, V. K.; *Angew. Chem. Int. Ed.* **2019**, 58, 5697.

[α]$^{20}_{D}$ = +32.2 (*c* = 1.00, $CHCl_3$).

^{1}H-NMR (300 MHz, $CDCl_3$) δ 7.47-7.29 (m, 5H), 5.56 (s, 1H), 5.18 (AB system, *J* = 12.1, 1H) 5.12 (AB system, *J* = 12.1, 1H), 4.36-4.31 (m, 1H), 3.98 (br d, *J* = 8.8 Hz, 2H), 2.58 (br q, *J* = 13.8 Hz, 2H), 1.86-1.77 (m, 1H), 1.72-1.63 (m, 2H), 1.45 (s, 9H), 1.12-1.00 (m, 2H), 0.96 (s, 9H). Spectrum

Spectroscopical data are in agreement with those reported in the literature.[13]

Benzyl (2*S*,4*S*)-4-(2-((*S*)-1-(*tert*-butoxycarbonyl)pyrrolidin-2-yl)ethyl)-2-(*tert*-butyl)-5-oxooxazolidine-3-carboxylate (4pe)

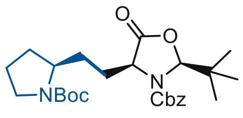

Synthetised by **GP-C** using benzyl *tert*-butyl (*S*)-2-(isocyanomethyl)pyrrolidine-1-carboxylate (**1p**, 21.0 mg, 0.10 mmol, 1 equiv.) and benzyl (*S*)-2-(*tert*-butyl)-4-methylene-5-oxooxazolidine-3-carboxylate (**2e**, 57.9 mg, 0.200 mmol, 2 equiv.). The crude product was purified by flash column chromatography (SiO_2; 20-40% ethyl acetate in pentane) affording 42.9 mg of product in 90% yield as a yellow oil. The product corresponds to the *cis*-diastereoisomer (*dr* ≥ 95:5). **R_f** = 0.46 (20% EtOAc in cyclohexane).

[α]$^{20}_{D}$ = +8.5 (*c* = 0.70, $CHCl_3$).

^{1}H-NMR (500 MHz, Tetrachloroethane-d2, 363 K) δ 7.36-7.27 (m, 5H), 5.50 (s, 1H), 5.16 (AB system, *J* = 12.1 Hz, 1H), 5.11 (AB system, *J* = 12.1 Hz, 1H), 4.16 (t, *J* = 7.2 Hz, 1H), 3.78-3.67 (m, 1H), 3.34 (dt, *J* = 10.9, 7.6 Hz, 1H), 3.20 (ddd, *J* = 10.8, 7.0, 5.6 Hz, 1H), 1.97 (tt, *J* = 12.7, 4.9 Hz, 1H), 1.93-1.82 (m, 2H), 1.81-1.67 (m, 3H), 1.53-1.43 (m, 2H), 1.40 (s, 9H), 0.93 (s, 9H). Spectrum

^{13}C-NMR (126 MHz, Tetrachloroethane-d2, 363 K) δ 172.5, 156.1, 154.7, 135.8, 128.9, 128.8, 128.5, 96.6, 79.2, 68.5, 57.7, 57.1, 46.6, 37.1, 32.1, 31.0, 30.7, 28.8, 25.2, 23.7. Spectrum

HRMS (ESI+): calculated for $C_{26}H_{38}N_2NaO_6$ $[M+Na]^+$: 497.2622; found: 497.2617.

---

[13] Merkens, K.; Aguilar Troyano, F. J.; Djossou, J.; Gómez-Suárez, A. *Ad. Synth. Cat.* **2020,** *362*, 2354-2359.

Benzyl (2*S*,4*S*)-2-(*tert*-butyl)-5-oxo-4-(2-((3a*R*,5*R*,5a*S*,8a*S*,8b*R*)-2,2,7,7-tetramethyltetrahydro-5*H*-bis([1,3]dioxolo)[4,5-b:4',5'-d]pyran-5-yl)ethyl)oxazolidine-3-carboxylate (4qe)

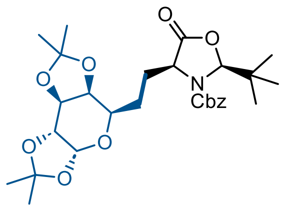

Synthetised by **GP-C** using (*3aR,5R,5aS,8aS,8bR*)-5 (isocyanomethyl)-2,2,7,7-tetramethyltetrahydro-5*H*-bis([1,3]dioxolo)[4,5-b:4',5'-d]pyran (**1q**, 26.9 mg, 0.10 mmol, 1 equiv.) and benzyl (*S*)-2-(tert-butyl)-4-methylene-5-oxooxazolidine-3-carboxylate (**2e**, 57.9 mg, 0.200 mmol, 2 equiv.). The crude product was purified firstly by flash column chromatography (SiO_2; 0-10% diethyl ether in DCM) followedby preparative TLC (30 % diethyl ether in pentane), affording 27.4 mg of product in 51% yield as a colourless oil. The product corresponds to the *cis*-diastereoisomer (*dr* ≥ 95:5). $\mathbf{R_f}$ = 0.40 (20% diethyl eter in pentane).

$[\alpha]^{20}_D$ = -6.4 (*c* = 0.70, $CHCl_3$).

^{1}H-NMR (500 MHz, $CDCl_3$) δ 7.40-7.32 (m, 5H), 5.55 (s, 1H), 5.50 (d, *J* = 5.1 Hz, 1H), 5.18 (s, 2H), 4.57 (dd, *J* = 7.9, 2.4 Hz, 1H), 4.35 (t, *J* = 7.6 Hz, 1H), 4.29 (dd, *J* = 5.1, 2.4 Hz, 1H), 4.10 (dd, *J* = 7.9, 2.0 Hz, 1H), 3.75 (ddd, *J* = 7.4, 5.3, 2.0 Hz, 1H), 2.17-2.00 (m, 2H), 1.94-1.84 (m, 1H), 1.81-1.71 (m, 1H), 1.48 (s, 3H), 1.44 (s, 3H), 1.33 (s, 3H), 1.32 (s, 3H), 0.96 (s, 9H). Spectrum

^{13}C-NMR (126 MHz, $CDCl_3$) δ 172.6, 156.3, 135.6, 128.8, 128.7, 128.6, 109.0, 108.4, 96.7, 72.6, 71.1, 70.6, 68.4, 67.0, 57.1, 37.0, 29.9, 29.3, 26.9, 26.18, 26.17, 25.08, 25.06, 24.6. Spectrum

HRMS (ESI+): calculated for $C_{28}H_{39}NNaO_9$ $[M+Na]^+$: 556.2517; found: 556.2509.

3.5. Scale-Up Synthesis of **4aa**

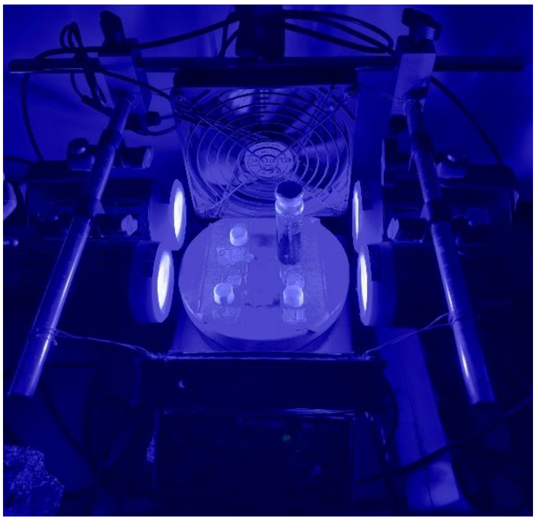

Figure S3. Scale-up synthesis reaction setup.

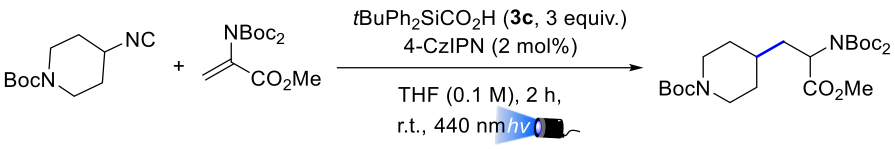

1a
1 equiv.

2a
2 equiv.

4aa

To a 15 ml oven dried vial equipped with a magnetic stir bar was added isonitrile **1aa** (210.3 mg, 1.00 mmol, 1 equiv.), Giese acceptor **2a** (602.7 mg, 2.00 mmol, 2 equiv.) silacarboxylic acid **3c** (853.3 mg, 3.00 mmol, 3 equiv.) and 4-CzIPN (15.8 mg, 2 mol%). The vial was sealed and then evacuated and refilled with argon (3x) before dry degassed THF was added (0.1 M, 10.0 mL). The reaction mixture was irradiated 2 hours with two Kessil PR160L-blue LED lamp (max 45 W High Luminous DEX 2100 LED, λ_{max} = 440 nm) as illustrated in the reaction setup (Figure S3). Solvent and volatiles were removed under reduced pressure, and the crude mixture was subjected to flash column chromatography (SiO_2; 20-40% diethyl ether in pentane). The title compound **4aa** was obtained as a colorless oil (442.0 mg, 0.91 mmol, 91%). $\mathbf{R_f}$ = 0.40 (20% EtOAc in cyclohexane).

^{1}H-NMR (300 MHz, $CDCl_3$) δ 4.94 (dd, J = 9.4, 5.1 Hz, 1H), 4.05 (br d, J = 13.2 Hz, 2H), 3.69 (s, 3H), 2.64 (br q, J = 12.3 Hz, 2H), 2.05-1.96 (m, 1H), 1.86-1.74 (m, 2H), 1.61-1.56 (m, 1H), 1.48 (s, 18H), 1.43 (s, 9H), 1.26-0.98 (m, 3H). Spectrum

4. Mechanistic Studies

4.1. Mechanistic Proposal for the Deaminative Alkylation

We envisioned a suitable photoredox catalytic cycle with the next mechanistic hypothesis (Figure S4). In this scenario, the excited photocatalyst [E^*_{red}(4CzIPN*/4CzIPN$^{\bullet-}$) = +1.35 V][14] can oxidate the silacarboxylate **A** as a species in equilibrium with the acid precursor [$E_{1/2}$($R_3SiCO2^{\bullet}$/R_3SiCO2^{-}) ≈ +1.3 V] to give the reduced 4CzIPN$^{\bullet-}$ species and radical intermediate **B** which upon decarboxylation, gives the *t*BuPh$_2$silyl radical.[15] The latter adds to the isonitrile forming the imidoyl radical, with the subsequent β-fragmentation originating the carbon-centered radical. This nucleophilic radical is then added to the electron-poor olefin **2** affording an alpha-carbonyl radical **C**, which is amenable to reduction by the reduced 4CzIPN$^{\bullet-}$ species [E_{red}(4CzIPN/4CzIPN$^{\bullet-}$) = -1.21 V][16]. In this way, the photoredox catalyst is regenerated and the catalytic cycle is closed. Finally, the alkyl anion **D** (stabilized as an enolate by the electron-withdrawing group, EWG) is protonated to form the product **4**.

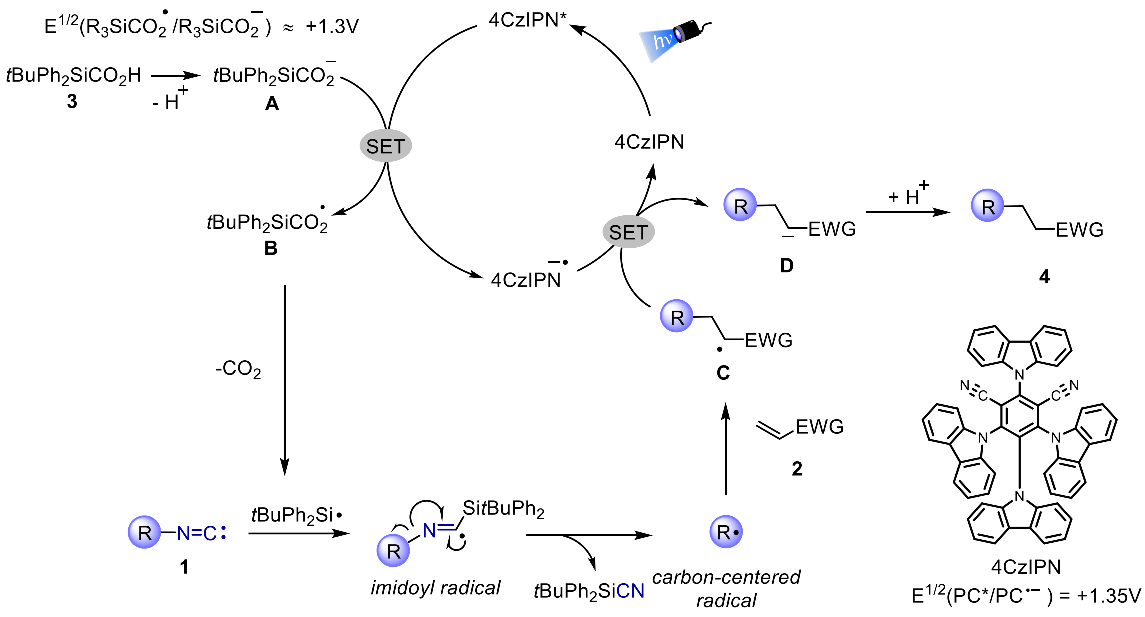

Figure S4. Plausible mechanism for the deaminative alkylation.

---

[14] Luo,J.; Zhang, J.; *ACS Catal.* **2016**, *6*, 873–877.

[15] Xu, N. X.; Li, B. X.; Wang, C.; Uchiyama, M. *Angew. Chem. Int. Ed.* **2020,** *59*, 10639-10644.

[16] Shang, T. Y.; Lu, L. H.; Cao, Z.; Liu, Y.; He, W. M. & Yu, B. *Chem. Commun.*, **2019**, *55*, 5408-5419.

4.2. Additional Experiments

Silacarboxylic Acid 3c in presence of tert-butyl acrylate

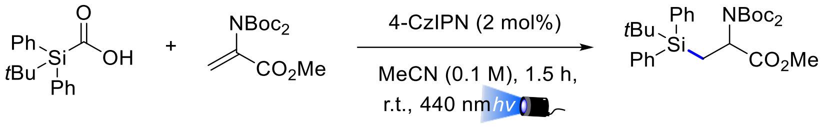

3c 1 equiv. **2a** 2 equiv. **5c**

An oven-dried 1 mL vial equipped with a magnetic stir bar, was charged with *tert*-butyldiphenylsilacarboxylic acid **3c** (28.4 mg, 0.100 mmol, 1 equiv.), electron-deficient olefin **2a** (60.3 mg, 0.200 mmol, 2 equiv.), 4-CzIPN (1.58 mg, 2.00 µmol, 2 mol%) and sealed with a septum cap. The vial was evacuated and refilled with argon (3x). Degassed THF was added (1 mL, 0.1 M) by syringe and the reaction mixture was irradiated with blue LEDs lamp (45 W, λ_{max} = 440 nm) at room temperature for 1.5 hours. Afterwards, solvent was removed *in vacuo* and the product was purified by flash column chromatography (SiO_2; 5-20% EtOAc in cyclohexane). The title compound **5c** was obtained as a colorless oil (197.7 mg, 0.20 mmol, 99%). $\mathbf{R_f}$ = 0.20 (10% EtOAc in cyclohexane).

^{1}H-NMR (300 MHz, $CDCl_3$) δ 7.68 – 7.61 (m, 4H), 7.42 – 7.30 (m, 6H), 4.97 (dd, *J* = 8.9, 5.1 Hz, 1H), 3.54 (s, 3H), 2.24 (dd, *J* = 15.6, 5.1 Hz, 1H), 1.84 (dd, *J* = 15.6, 8.9 Hz, 1H), 1.35 (s, 18H), 1.01 (s, 9H). Spectrum

Spectroscopical data are in agreement with those reported in the literature.[17]

[17] Wan, Y.; Zhu, J.; Yuan, Q.; Wang, W. & Zhang, Y. *Org. Lett.* **2021**, *23*, 1406-1410.

4.3. Quantum Yield

The quantum yield of the reaction was determined using previously reported procedures.[18] The quantum yield of a reaction is defined as:

$$\phi = \frac{mol\ of\ product\ formed}{F \cdot t \cdot [1 - 10^{-A(\lambda)}]} \qquad (eq. 1)$$

where ϕ is the quantum yield of the reaction, t is the time of the reaction(s), $[1\text{-}10^{-A(\lambda)}]$ is the ratio of absorbed photons by the solution and F is the photon flux calculated by standard ferrioxalate actinometry.

A ferrioxalate actinometry solution was prepared following the Hammond variation of the Hatchard and Parker procedure outlined in Handbook of Photochemistry.[19] This method is based on the decomposition of ferric ions to ferrous ions, which are complexed by 1,10-phenanthroline and monitored by UV/Vis absorbance at 510 nm. The moles of iron-phenanthroline complex formed are related to moles of photons absorbed.

Experimental: The following solutions were prepared in the dark (flasks were wrapped in aluminum foil) and stored in the dark at room temperature:

- Potassium ferrioxalate solution: 294.8 mg of potassium ferrioxalate and 139 μL of sulfuric acid (96 %) were added to a 50 mL volumetric flask and filled with water (MilliQ grade).
- Phenanthroline solution: 0.2 % by weight of 1,10-phenanthroline in water was prepared in a 50 mL volumetric flask.
- Buffer solution: 2.47 g of NaOAc and 0.5 mL of sulfuric acid (96 %) were added to a 50 mL volumetric flask and filled with water (MilliQ grade).
- Reaction solution: Following **GP-C**, *tert*-butyl 4-(isocyanomethyl) piperidine-1-carboxylate (**1a**, 21.0 mg, 0.100 mmol, 1 equiv.) methyl 2-(bis(*tert*-butoxycarbonyl)amino)acrylate (**2a**, 60.3 mg, 0.200 mmol, 2 equiv.) *tert*-

---

[18] a) Cuadros, S.; Rosso, C.; Barison, G.; Costa, P.; Kurbasi, M.; Bonchio, M.; Prato, M.; Filippini, G.; Dell'Amico, L. *Org. Lett.* **2022**, *16*, 2961 – 2966. b) Georgiou, E.; Spinnato, D.; Chen, K.; Melchiorre, P.; Muñiz, K. *Chem. Sci.* **2022**, *13*, 8060 – 8064. c) Piedra, H. F.; Plaza, M. *Chem. Sci.* **2023**, *14*, 650 – 657.

[19] Murov, S. L.; Handbook of Photochemistry, Marcel Dekker, New York, 1973.

butyldiphenylsilacarboxylic acid (**3c**, 85.3 mg, 0.300 mmol, 3 equiv.) and 4-CzIPN (1.58 mg, 2.00 µmol, 2 mol%) in THF (1.00 ml) were used.

Procedure:

1. A 4 mL glass vial was charged with 1 mL of the potassium ferrioxalate solution and placed 5 cm away from the 440 nm Kessil lamp. To monitor the decomposition reaction, this step was performed five different times varying only the irradiation time for each sample: 0, 2, 4, 6 and 10 seconds. All were irradiated at 25% intensity of the 440 nm LED Kessil lamp.

2. After irradiation, all the actinometer solution was transferred to a 10 mL volumetric flask. To this flask, 0.5 mL of the phenanthroline solution and 2 mL of buffer solution were added and filled with water (MilliQ grade).

3. The reaction was stirred in the dark for 1 hour to allow the ferrous ions to coordinate completely to the phenanthroline.

4. The UV-Vis spectra of actinometry samples were recorded for each time interval (**Figure S5**). The absorbance of the actinometry solution was monitored at 510 nm.

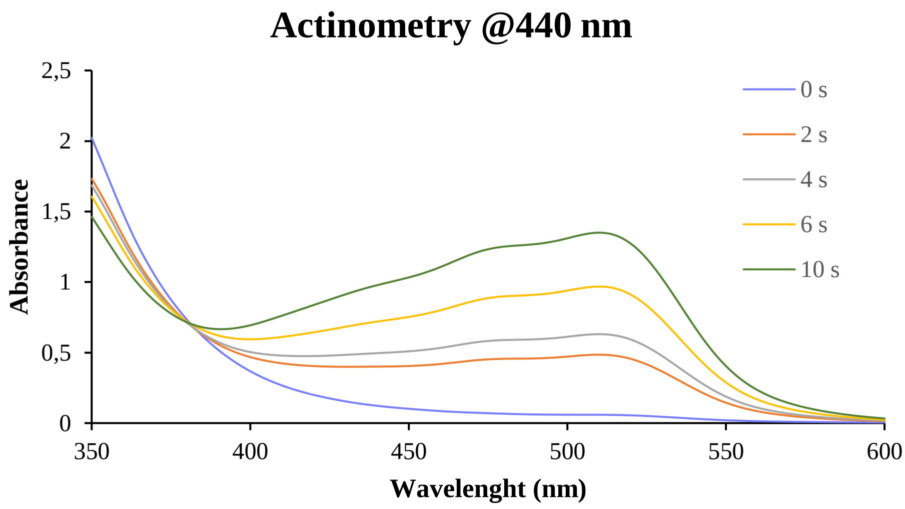

Figure S5: UV-Vis spectra of actinometry samples irradiated with 440 nm Kessil during different time periods.

5. The moles of Fe^{2+} formed (N) is determined using Lambert-Beer's Law (eq. 2).

$$mmol\,Fe^{2+} = \frac{V_1 \cdot V_3 \cdot \Delta A_{(510\,nm)}}{10^3 \cdot V_2 \cdot l \cdot \varepsilon_{(510\,nm)}} \qquad (eq. 2)$$

where V_1 is the irradiated volume (1 mL), V_2 is the aliquot of the irradiated solution taken for the determination of the ferrous ions (1 mL), V_3 is the final volume after complexation with phenanthroline (10 mL), l is the optical path-length of the irradiation cell (1 cm), $\Delta A_{(510\ nm)}$ the optical difference in absorbance between the irradiated solution and that taken in the dark, $\varepsilon_{(510\ nm)}$ is the molar extinction coefficient of the complex $Fe(phen)_3{}^{2+}$ (11100 L mol^{-1} cm^{-1}).

6. The moles of Fe^{2+} formed (N) are plotted as a function of time (t). The slope of this line (dN/dt) was correlated to the moles of incident photons by unit of time (F), according to the following equation (eq. 3).

$$\Phi Fe^{2+} = \frac{{dN}/{dt}}{F \cdot [1 - 10^{-A(\lambda)}]} \qquad (eq. 3)$$

where dN/dt is the rate of change of this property, the quantum yield ϕ Fe^{2+} at 456 nm is 1.1,[20] $[1 - 10^{-A(\lambda)}]$ is the ratio of absorbed photons by the solution, and A(λ) is the absorbance of the ferrioxalate solution at the wavelength used to carry out the experiments (440 nm). The absorbance of this solution at 440 nm is 0.059. Therefore, F, which is the photon flux, was determined to be $9.88 \cdot 10^{-7}$.

[20] C. A. Holubov and C. H. Langford, Wavelength and temperature dependence in the photolysis of the chemical actinometer, potassium trisoxalatoferrate(III), at longer wavelengths. Inorg. Chim. Acta., 1981, 53, 59–60.

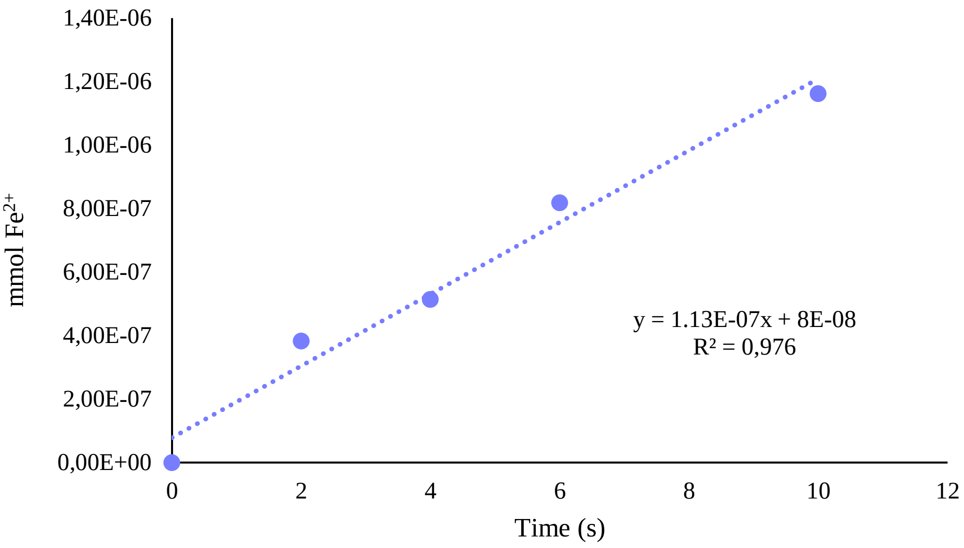

Figure S6: Moles of Fe^{2+} formed after irradiation with 440 nm Kessil as a function of time.

7. The reaction solution was irradiated using the same system described above for the actinometry experiment. The moles of product formed were determined by ^{1}H-NMR analysis using 1,3,5-trimethoxybenzene as internal standard at different intervals of time. The moles of product per unit of time are related to the number of photons absorbed by the use of equation (eq. 1).

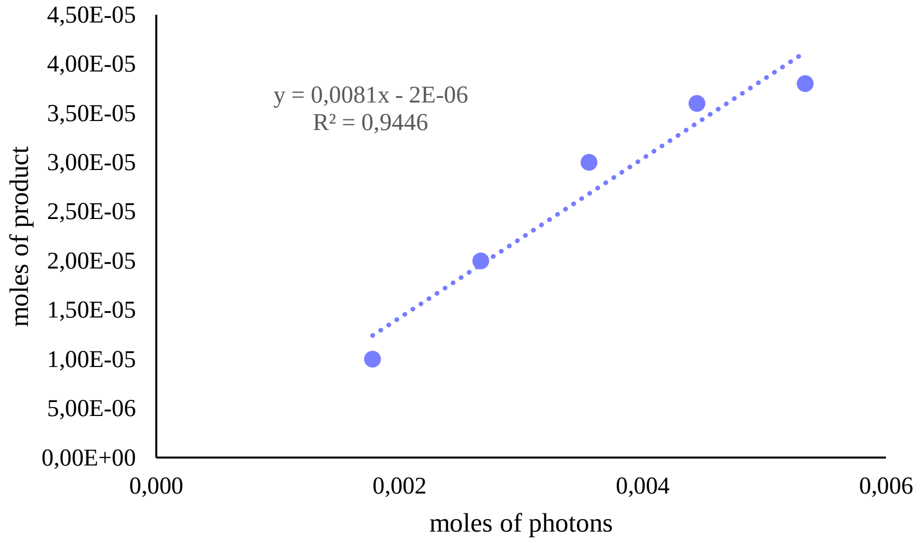

Figure S7: Moles of product **4f** formed related to the moles of incident photons.

In agreement with this, if we plot the moles of product formed (**4f**) vs. the moles of incident photons (photon flux · reaction time), the slope is equal to: $\phi \cdot [1-10^{-A(440\ nm)}]$,

where ϕ is the quantum yield to determined and A(440 nm) is the absorption of the reaction under study. For the model reaction mixture, the absorbance was determined over 2.00 so no correcting factor was needed. **The quantum yield (ϕ) of the photochemical transformation was measured to be 0.01.**

4.4. Stern-Volmer Experiment

Stern-Volmer quenching studies were conducted on an JASCO FP-8600 Spectrofluorometer. Samples of 4CzIPN, isonitrile **1a**, olefin **2a** and silacarboxylic acid **3c**, were prepared as degassed solutions in THF. The concentration of 4CzIPN was $1.0 \cdot 10^{-4}$ M. Samples were sealed in a 1 cm quartz cuvette. The sample solution was excited at 400 nm, with emission intensity detected at 524 nm. The emission light was acquired from 420 nm to 750 nm.

For each reagent 6 solutions with different concentrations were prepared to measure the fluorescence spectra. For the silacarboxylic acid **3c** we additionally added to the solution TMG (tetramethylguanidine, 20 mM) to shift the deprotonation equilibrium with a redox "non-active" base (it cannot be oxidized by 4CzIPN*).

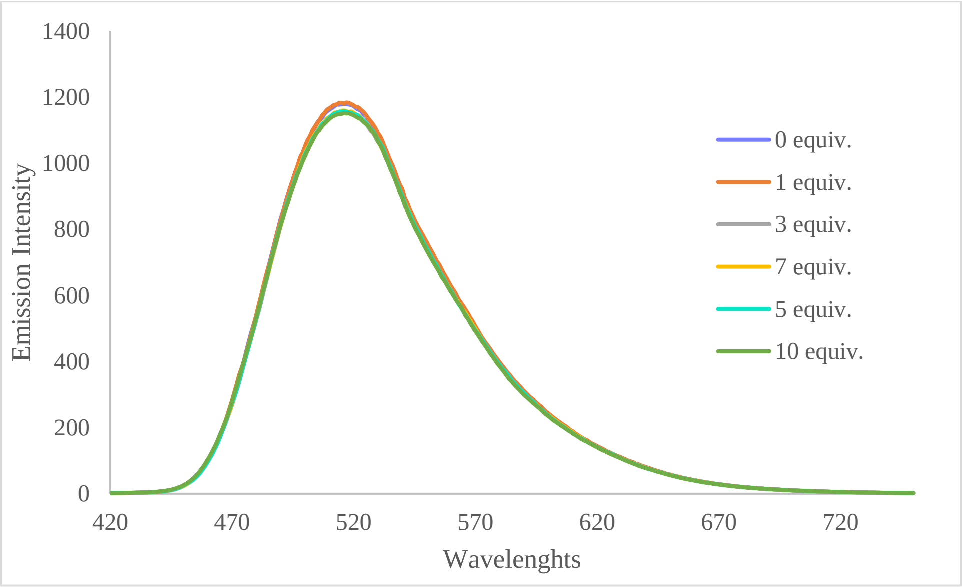

Figure S8. Quenching of the photocatalyst emission ($1.0 \cdot 10^{-4}$ M in THF) with increasing amounts of isonitrile **1a**.

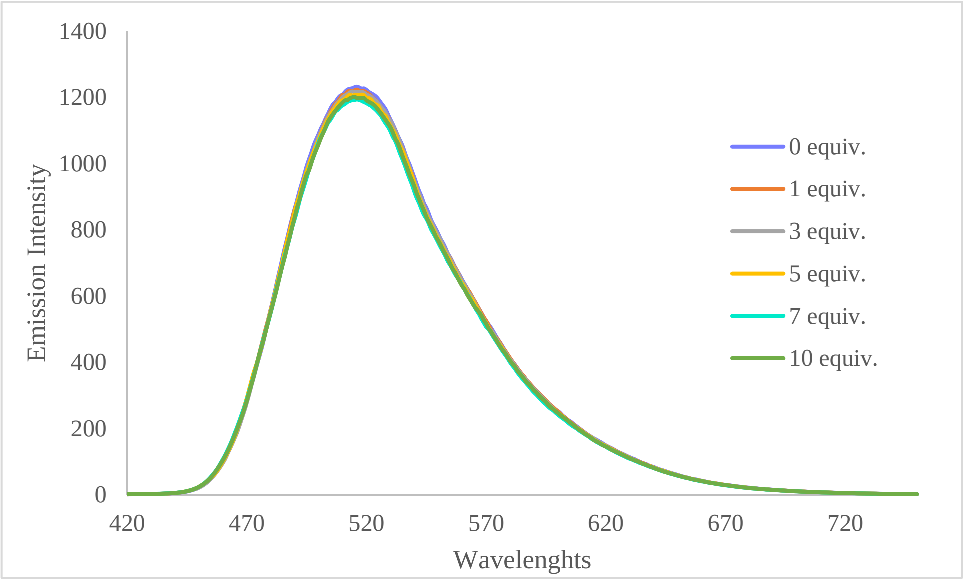

Figure S9. Quenching of the photocatalyst emission ($1.0 \cdot 10^{-4}$ M in THF) with increasing amounts of olefin **2a**.

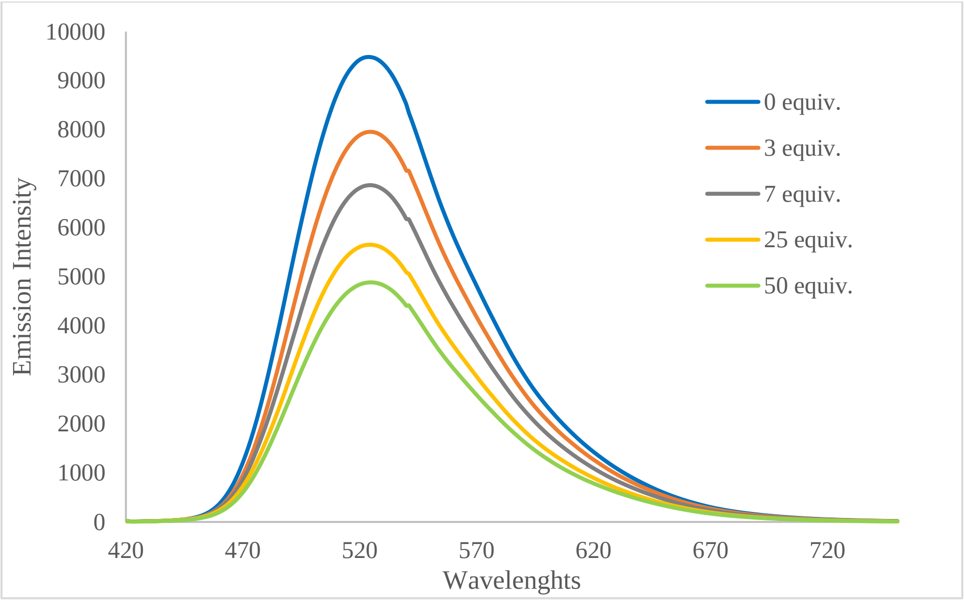

Figure S10. Quenching of the photocatalyst emission ($1.0 \cdot 10^{-4}$ M in THF) with increasing amounts of silacarboxylic acid **3c** and in presence of 20 mM of TMG as the base.

The comparative plot, reported in Figure S12 shows a linear correlation between the relative intensity I_0/I and the concentration of each quencher.

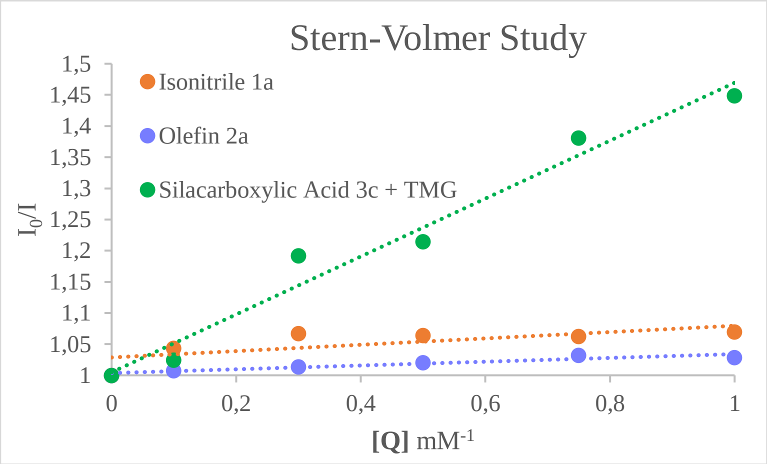

Figure S11. Comparative Stern-Volmer quenching plot

Based on equation (eq. 4), it is possible to calculate the Stern-Volmer constant K_{SV} (Table X)

$$\frac{I_0}{I} = K_{SV}[Q] + 1 \qquad (eq. 4)$$

	Isonitrile **1a**	Olefin **2a**	Silacarboxylic acid **3c**
**K_{SV} (M^{-1})**	26.0	30.4	464.7

5. NMR Spectra

For known compounds, only ^{1}H-NMR spectra is provided. For new compounds, full characterization is provided.

^{1}H-NMR (300 MHz, $CDCl_3$) of compound **SI-4**

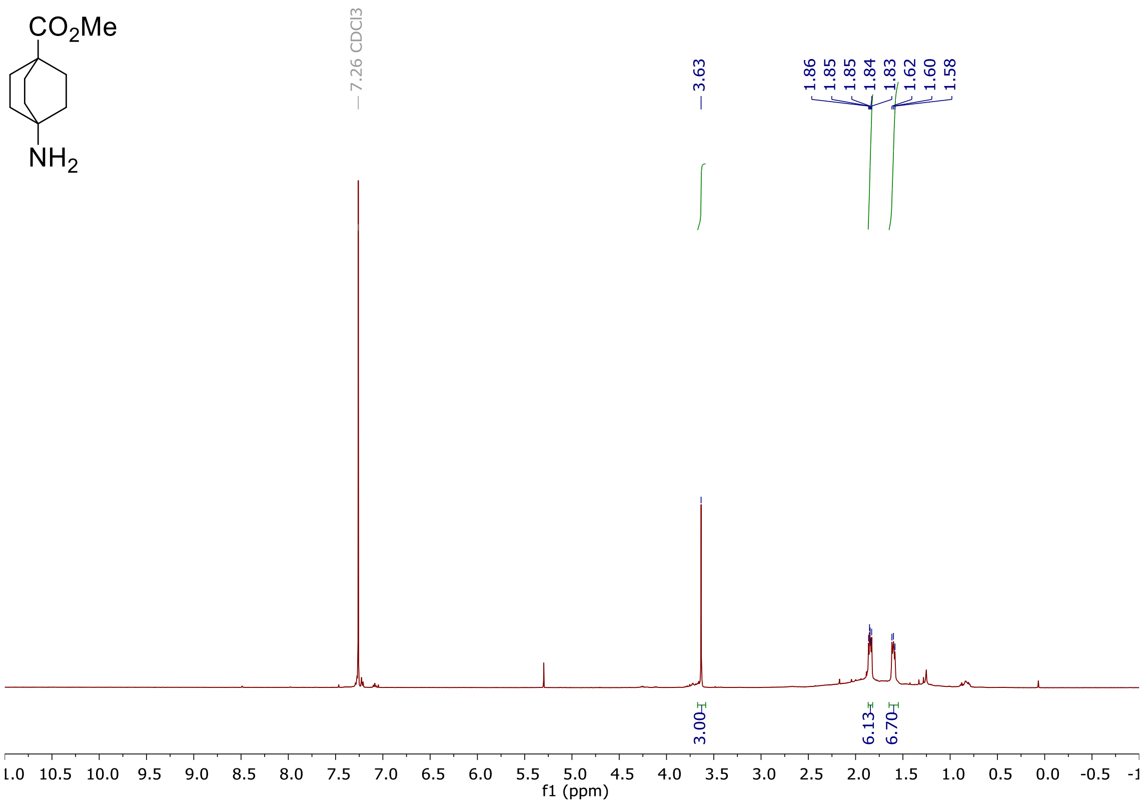

^{1}**H-NMR** (300 MHz, $CDCl_3$) of compound **SI-5**

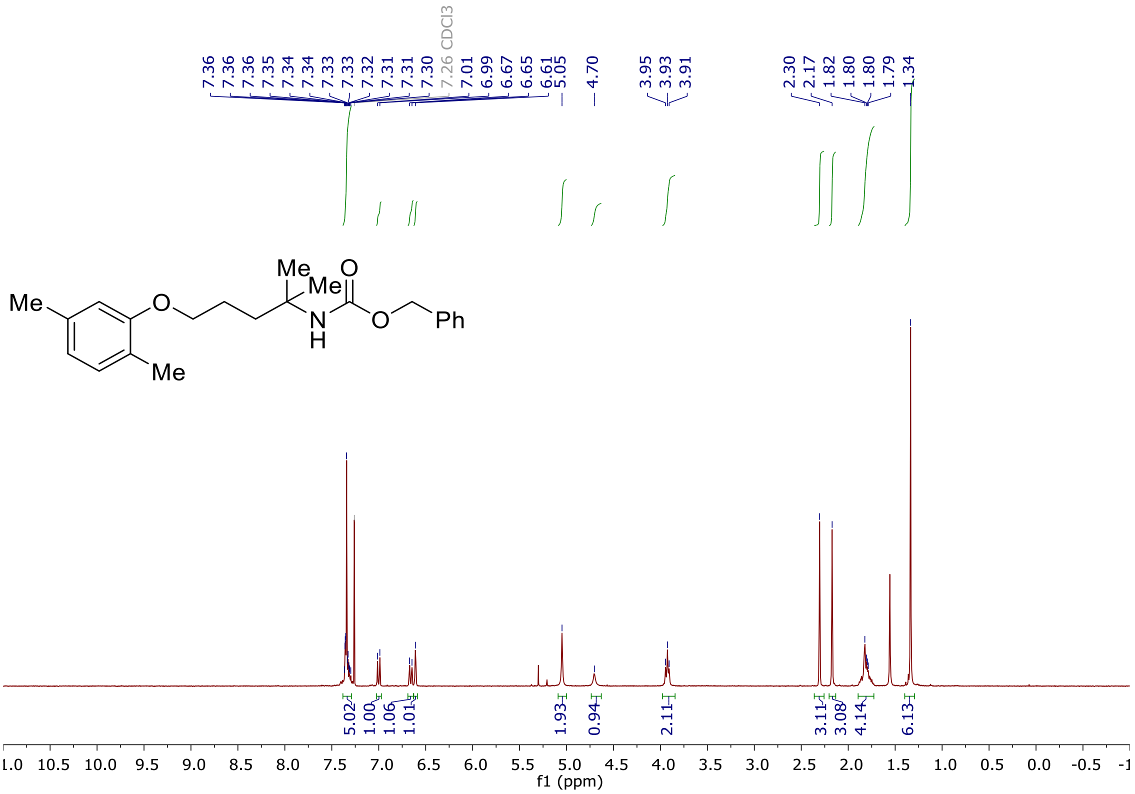

^{13}**C-NMR** (75 MHz, $CDCl_3$) of compound **SI-5**

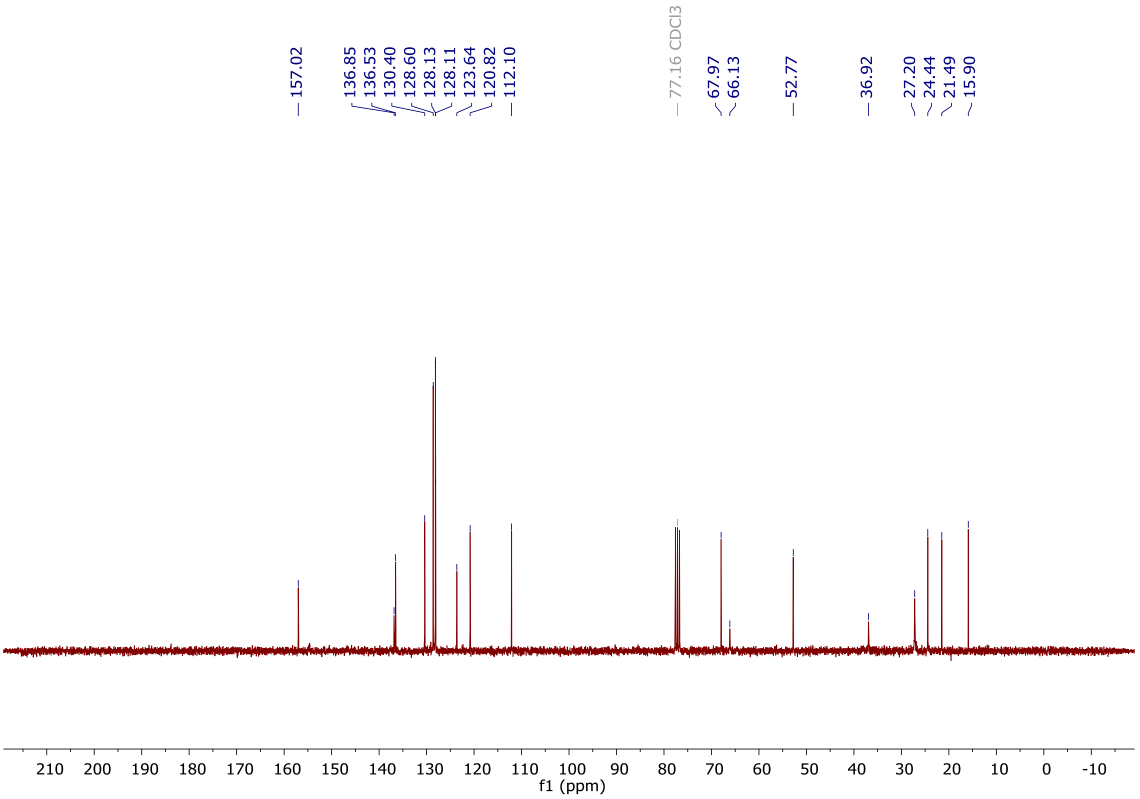

^{1}H-NMR (300 MHz, $CDCl_3$) of compound **SI-6**

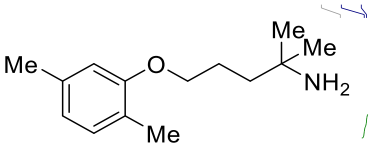

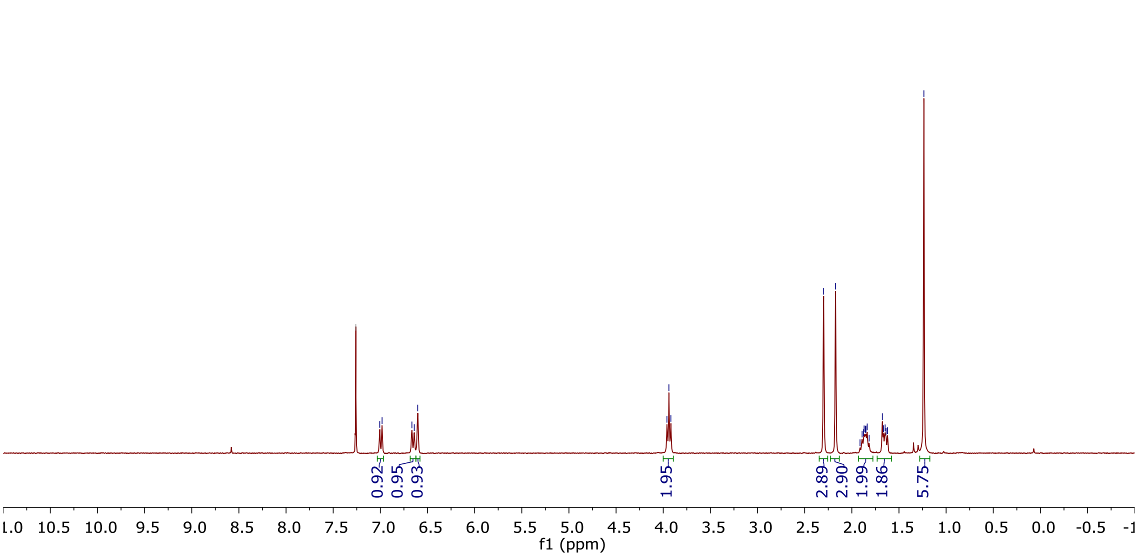

^{13}C-NMR (75 MHz, $CDCl_3$) of compound **SI-6**

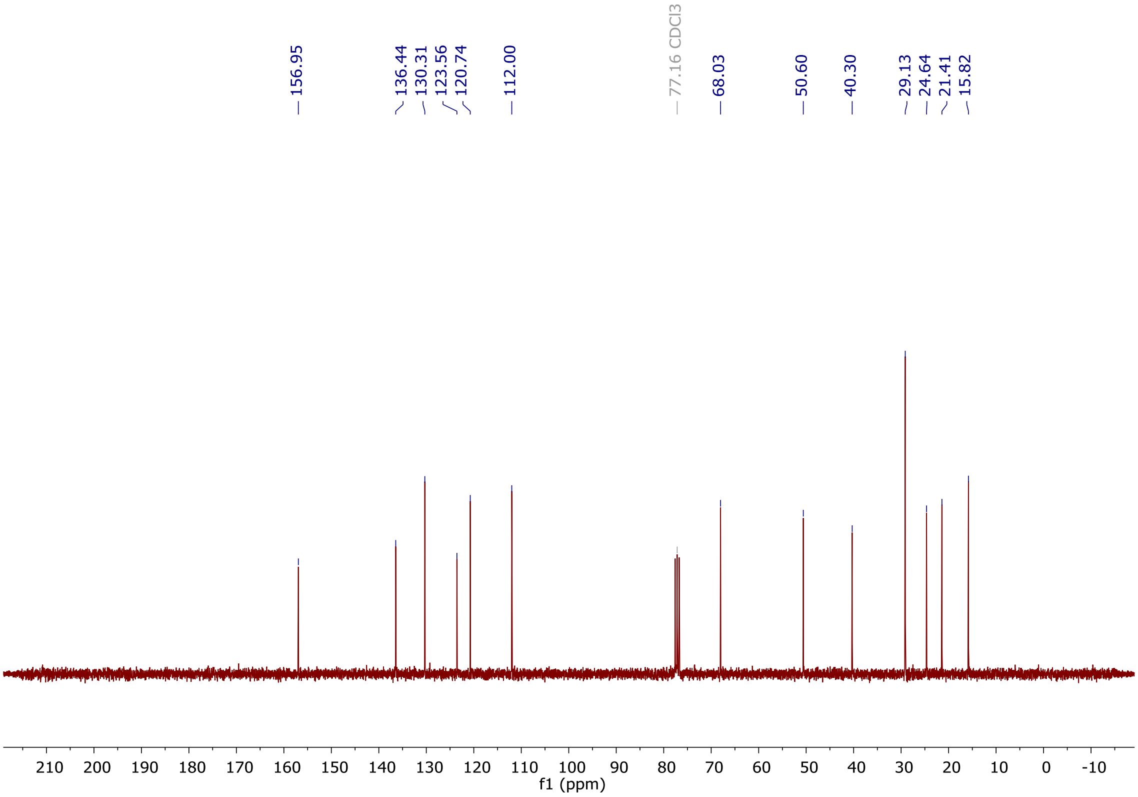

^{1}H-NMR (500 MHz, $CDCl_3$) of compound **1e**

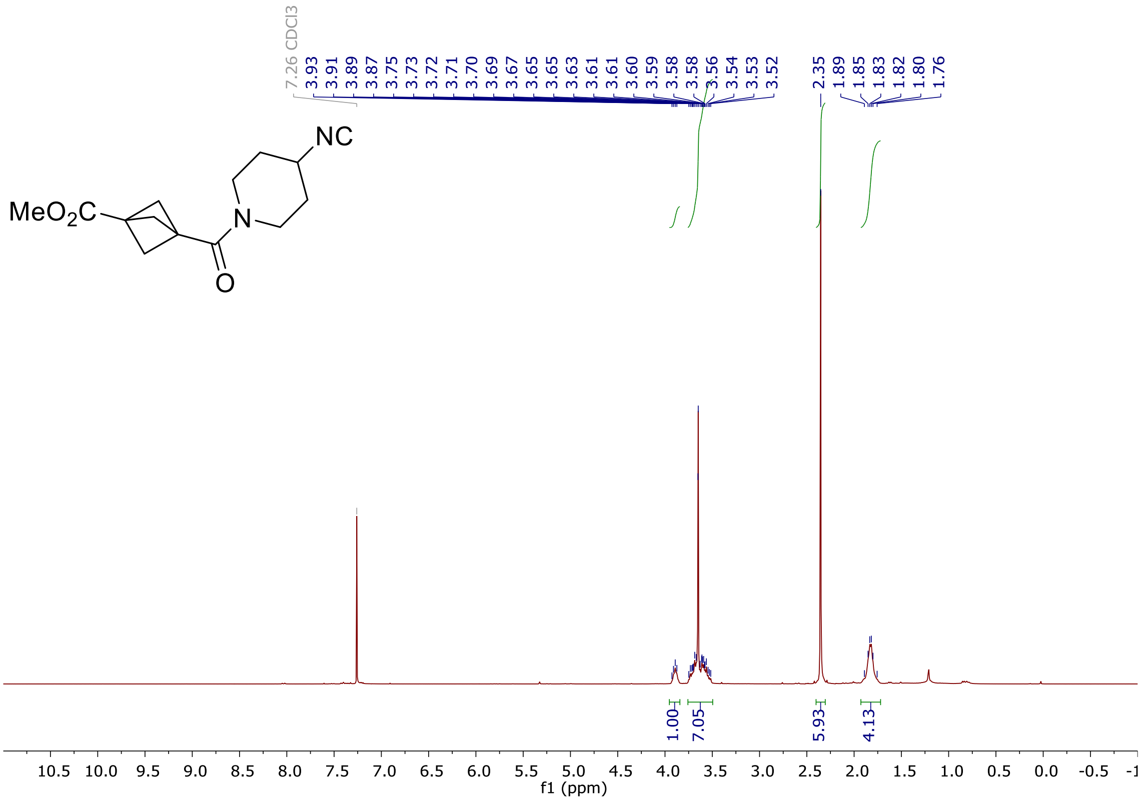

^{13}C-NMR (126 MHz, $CDCl_3$) of compound **1e**

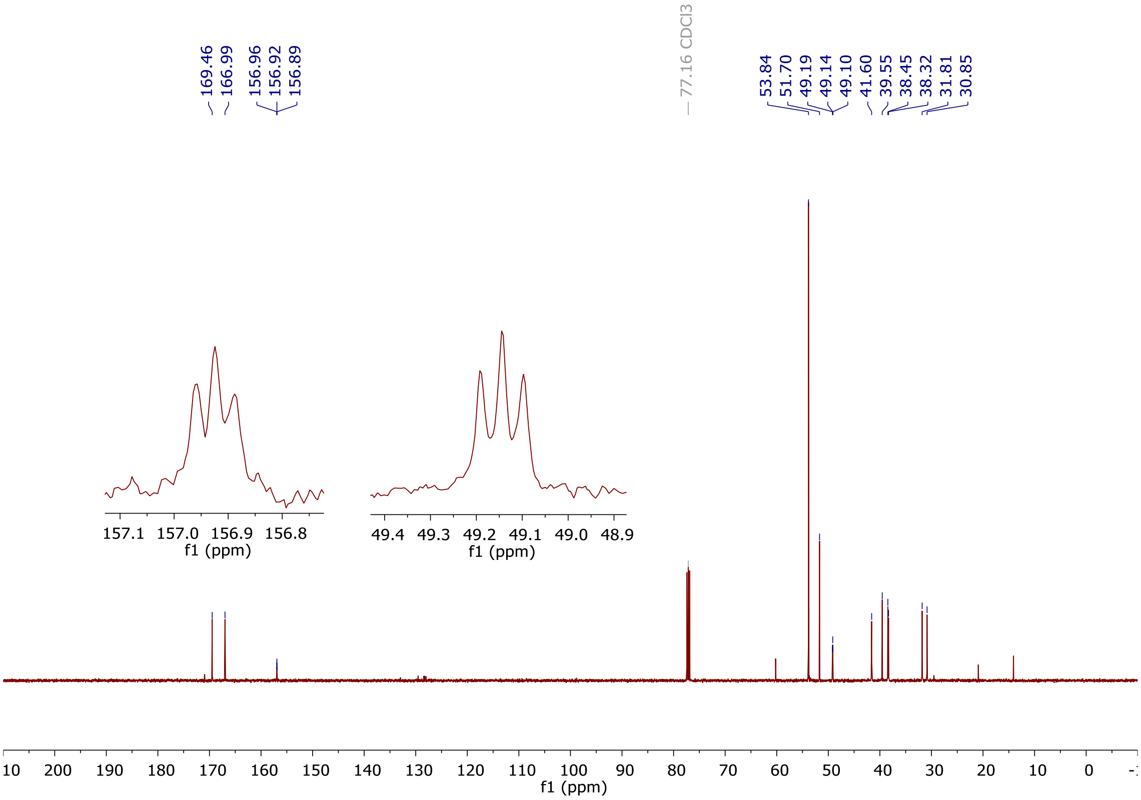

^{1}H-NMR (300 MHz, $CDCl_3$) of compound **1i**

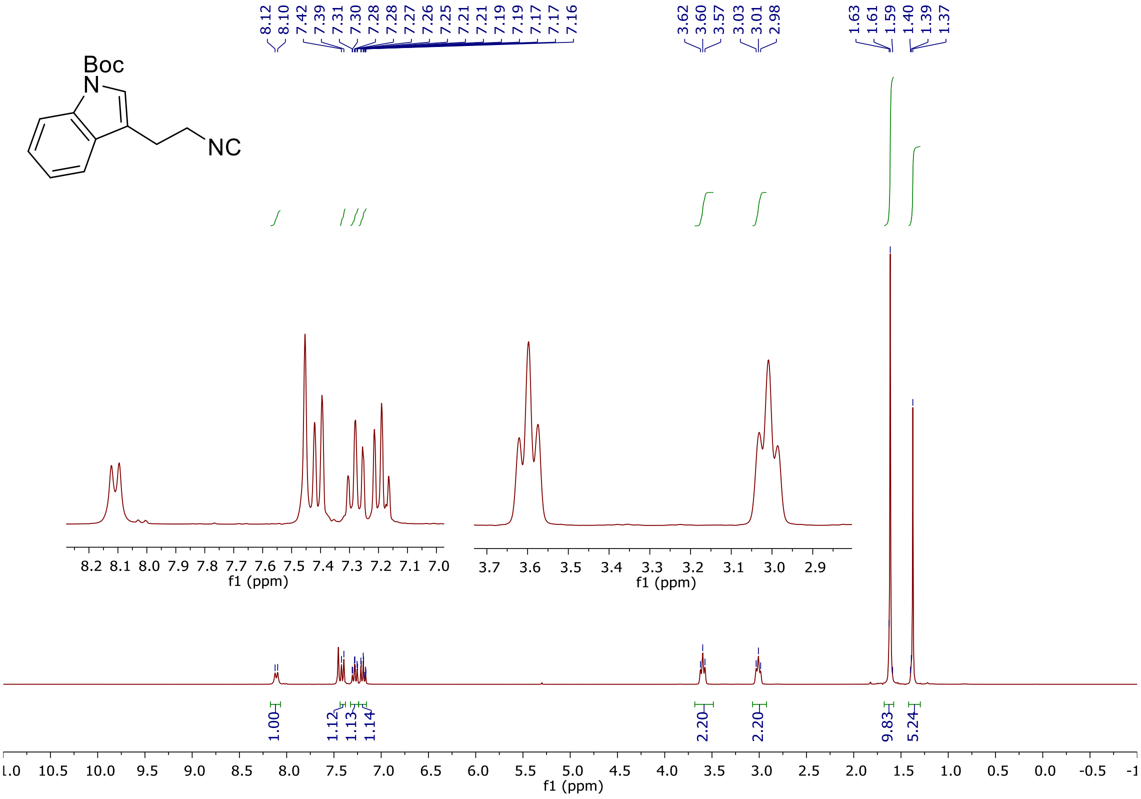

^{1}H-NMR (300 MHz, $CDCl_3$) of compound **1l**

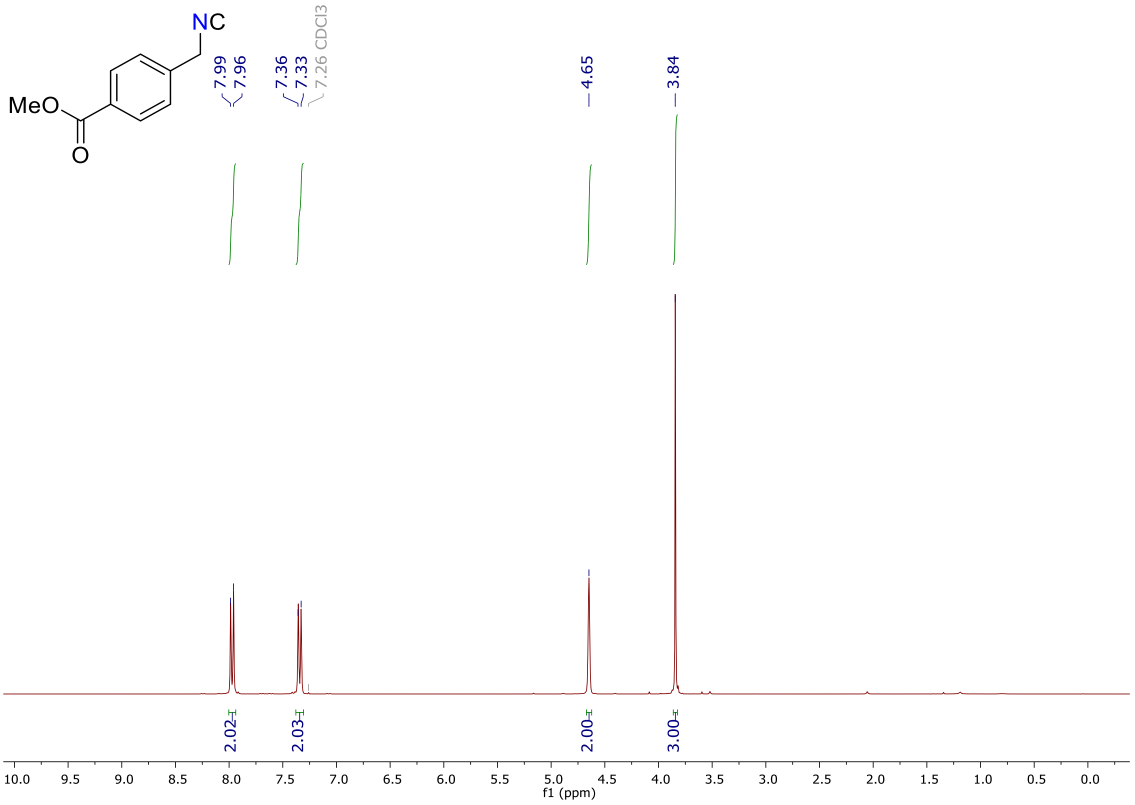

^{13}C-NMR (126 MHz, $CDCl_3$) of compound **1l**

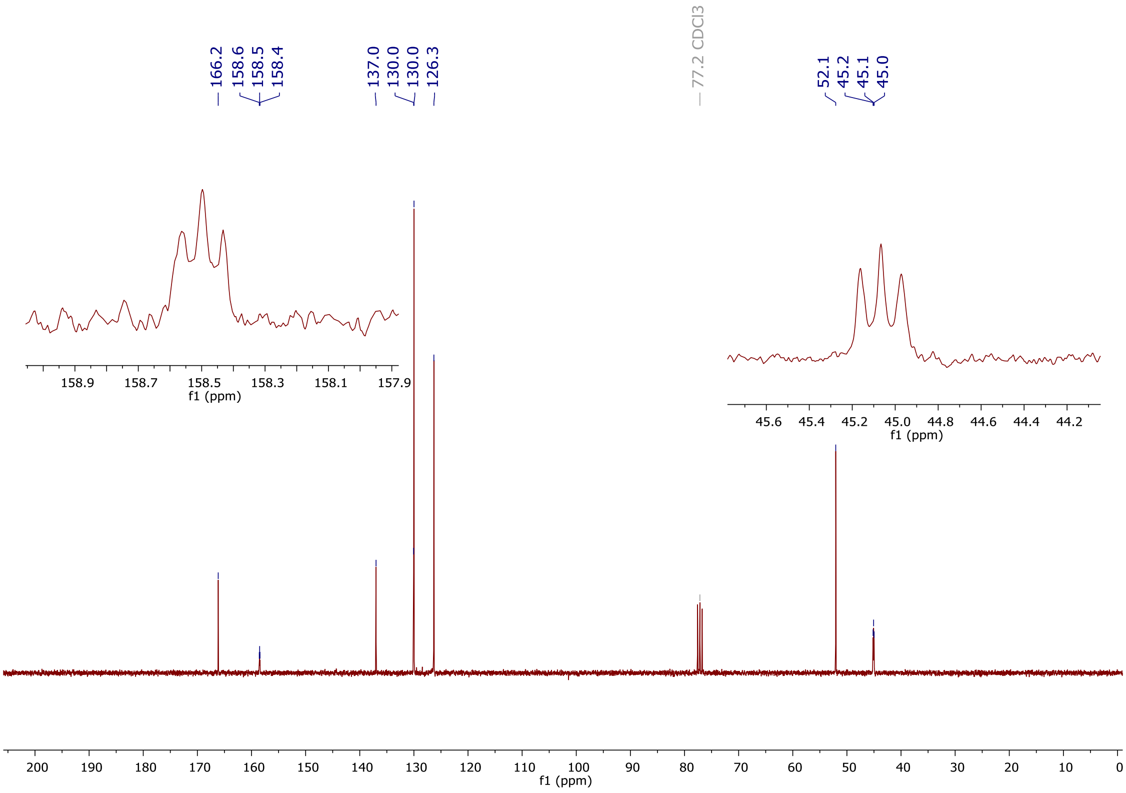

^{1}H-NMR (500 MHz, $CDCl_3$) of compound **1n**

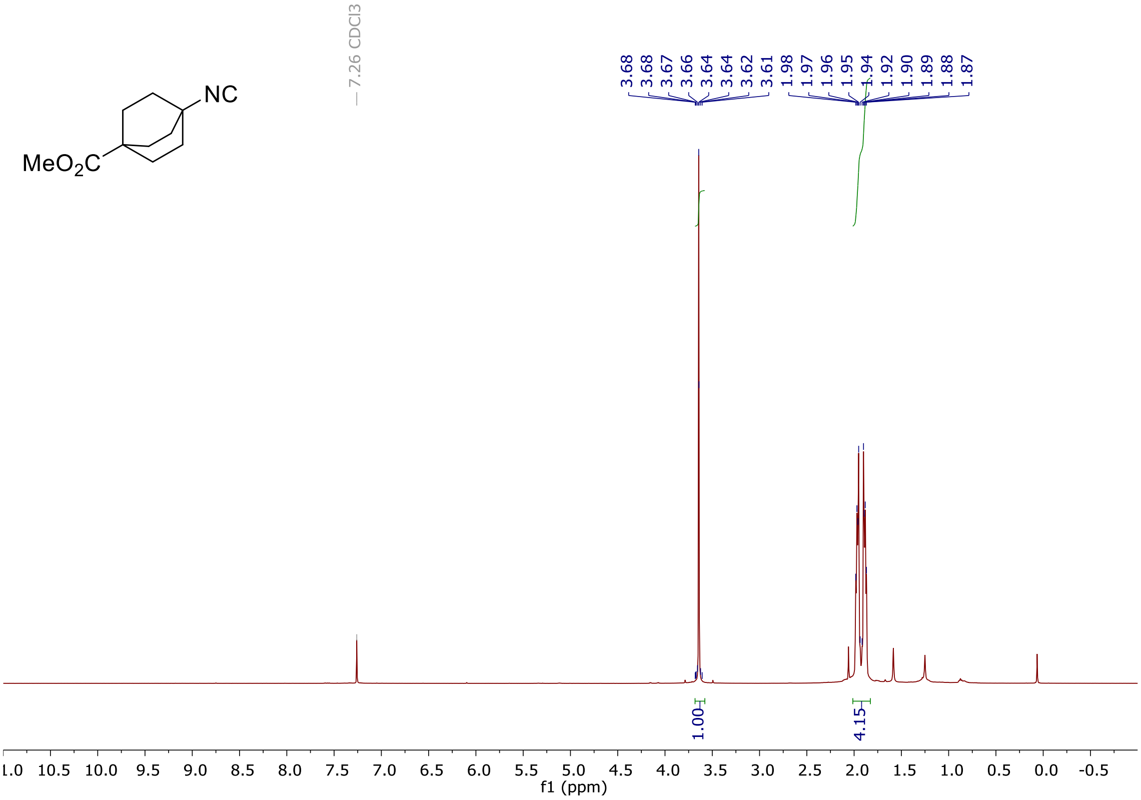

^{13}C-NMR (126 MHz, $CDCl_3$) of compound **1n**

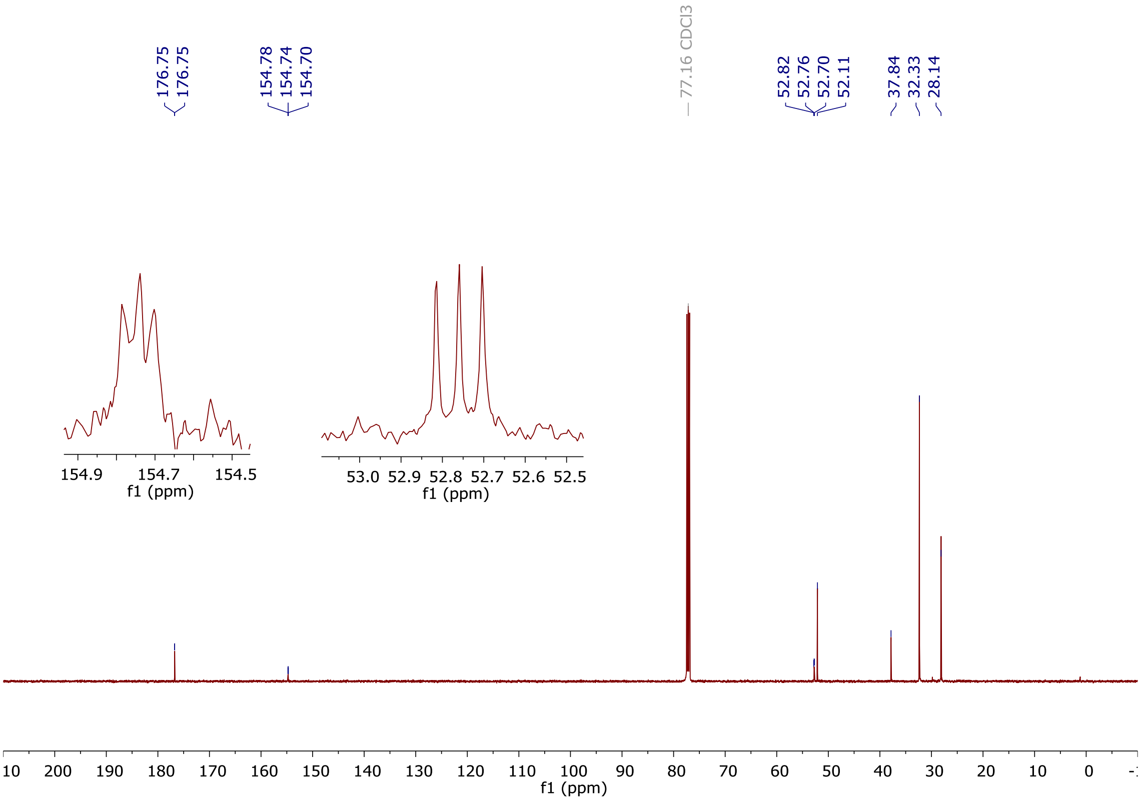

^{1}H-NMR (300 MHz, $CDCl_3$) of compound **1o**

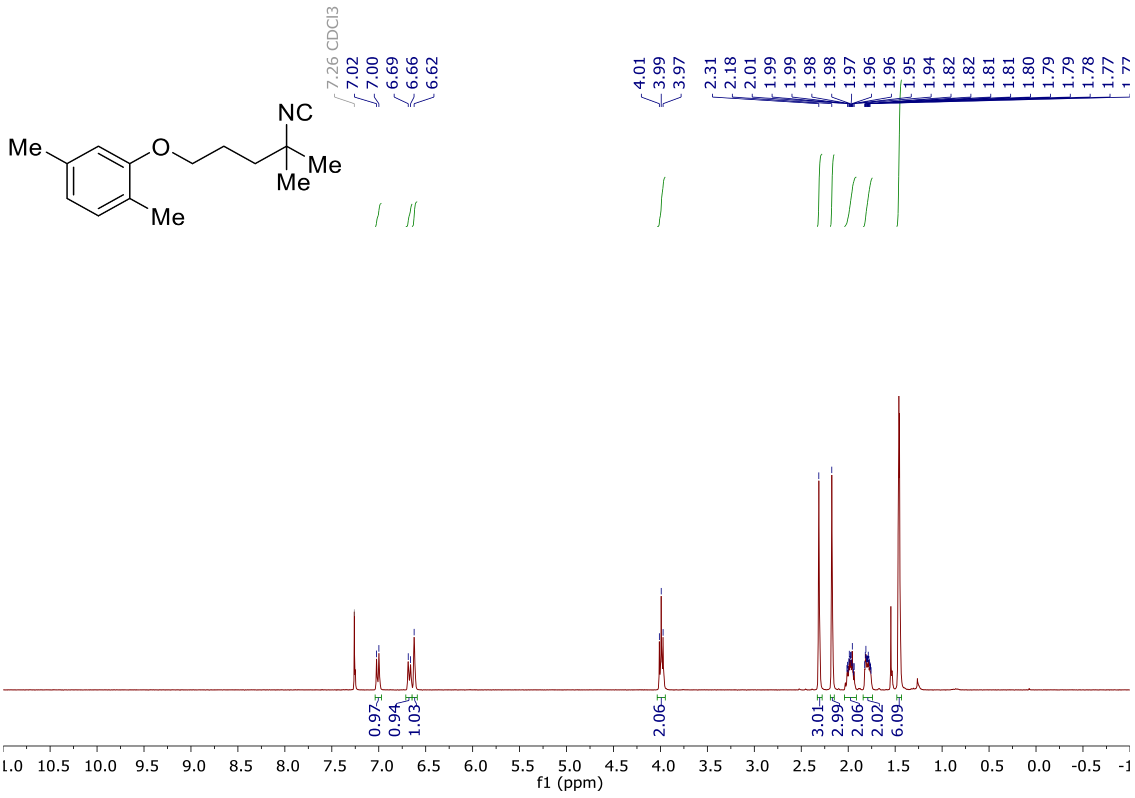

^{13}C-NMR (75 MHz, $CDCl_3$) of compound **1o**

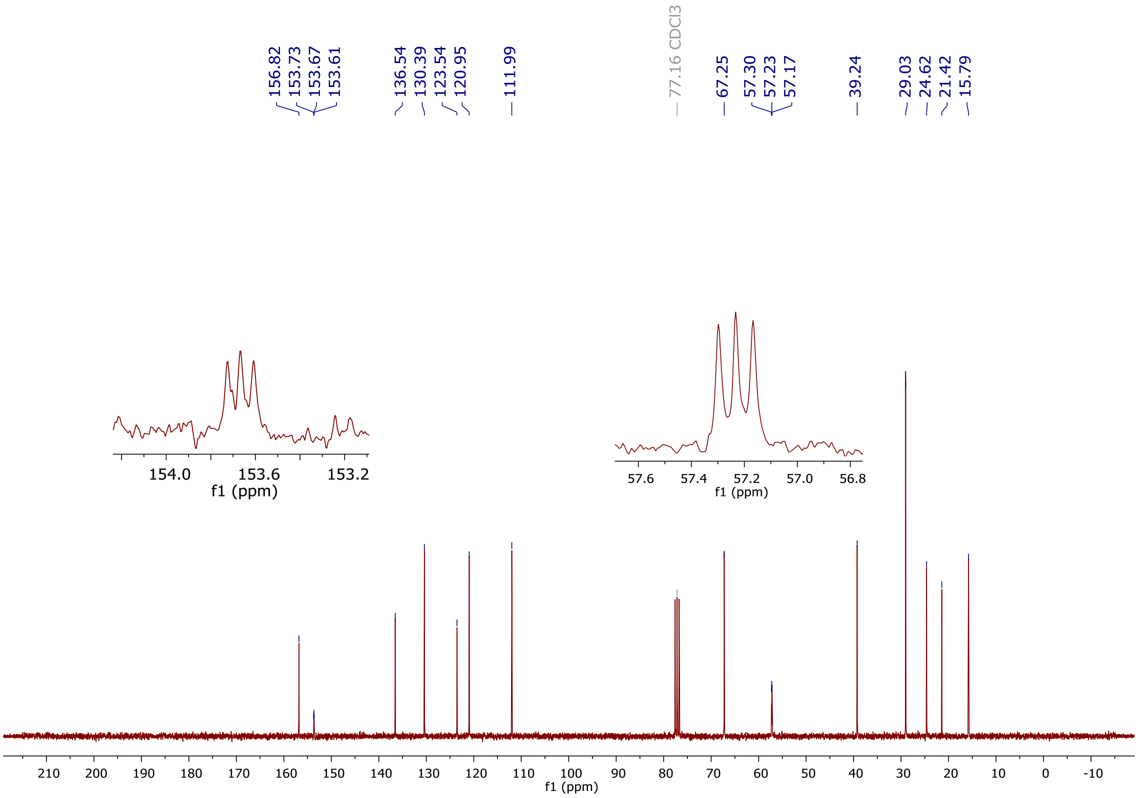

^{1}H-NMR (300 MHz, $CDCl_3$) of compound **1p**

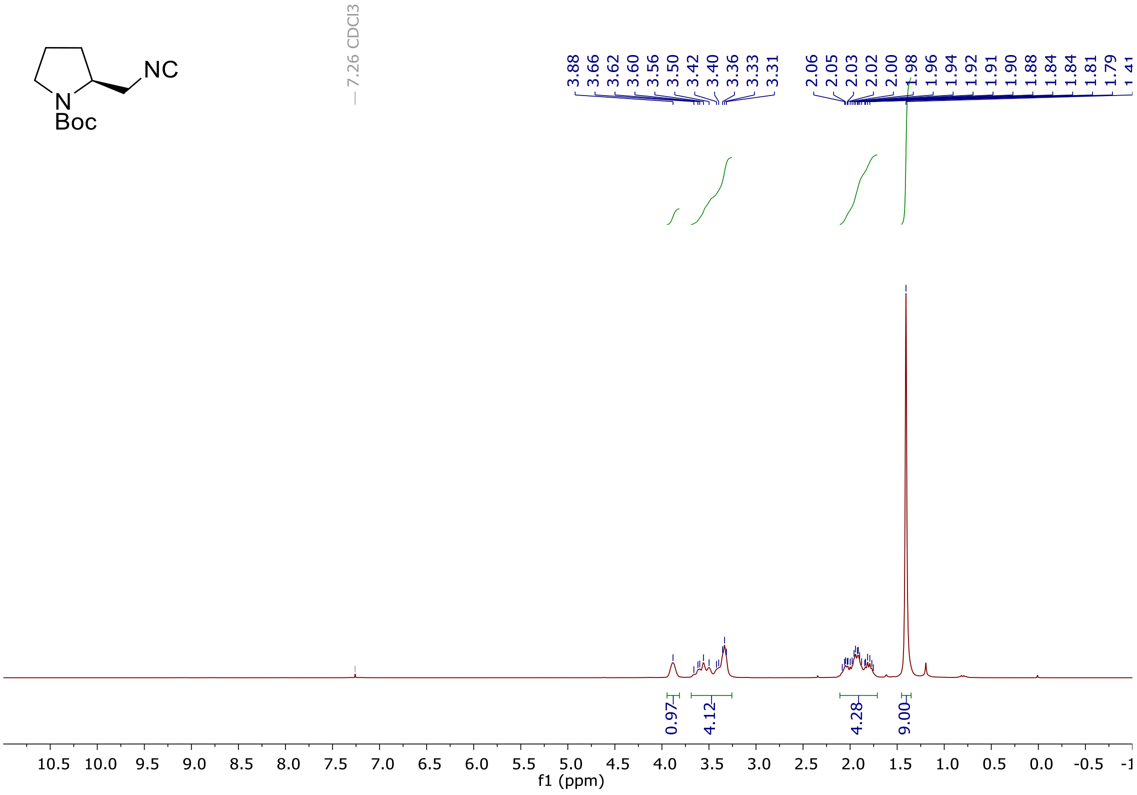

^{1}H-NMR (300 MHz, CDCl3) of compound **4aa**

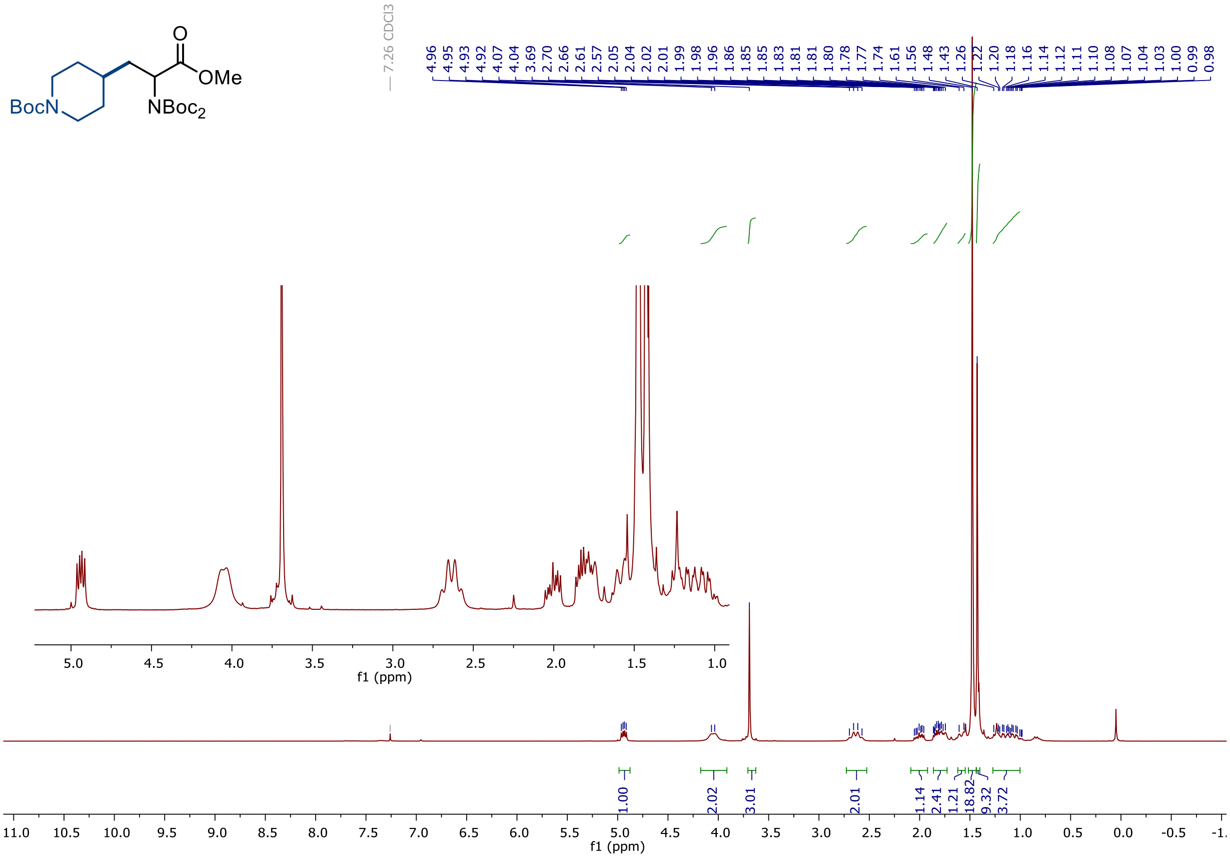

^{1}H-NMR (300 MHz, CDCl3) of compound **4ba**

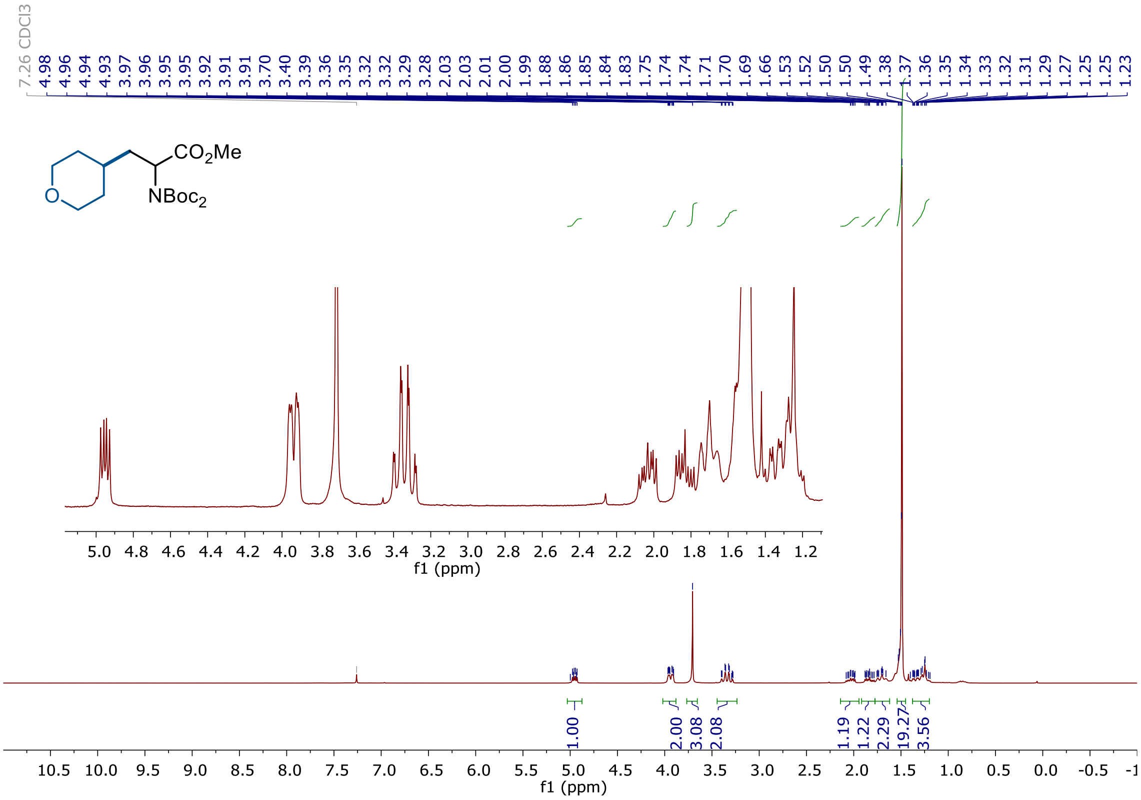

^{1}H-NMR (500 MHz, CDCl3) of compound **4ca**

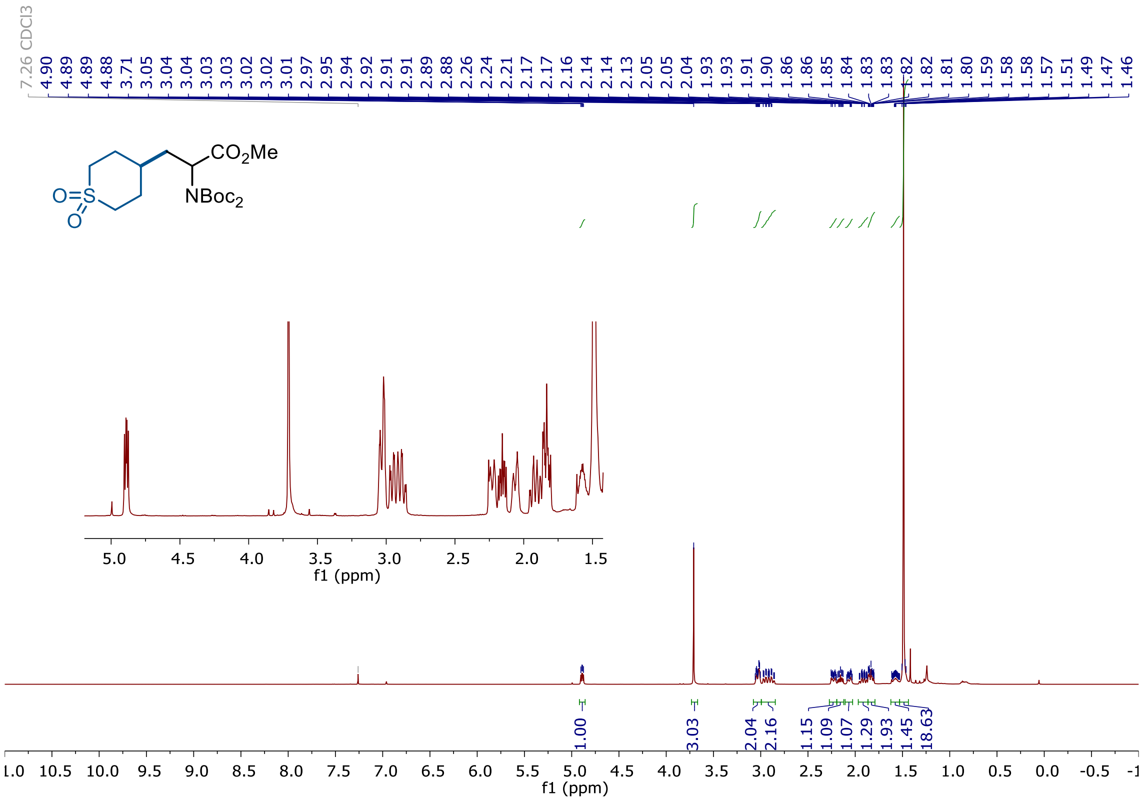

^{13}C-NMR (126 MHz, CDCl3) of compound **4ca**

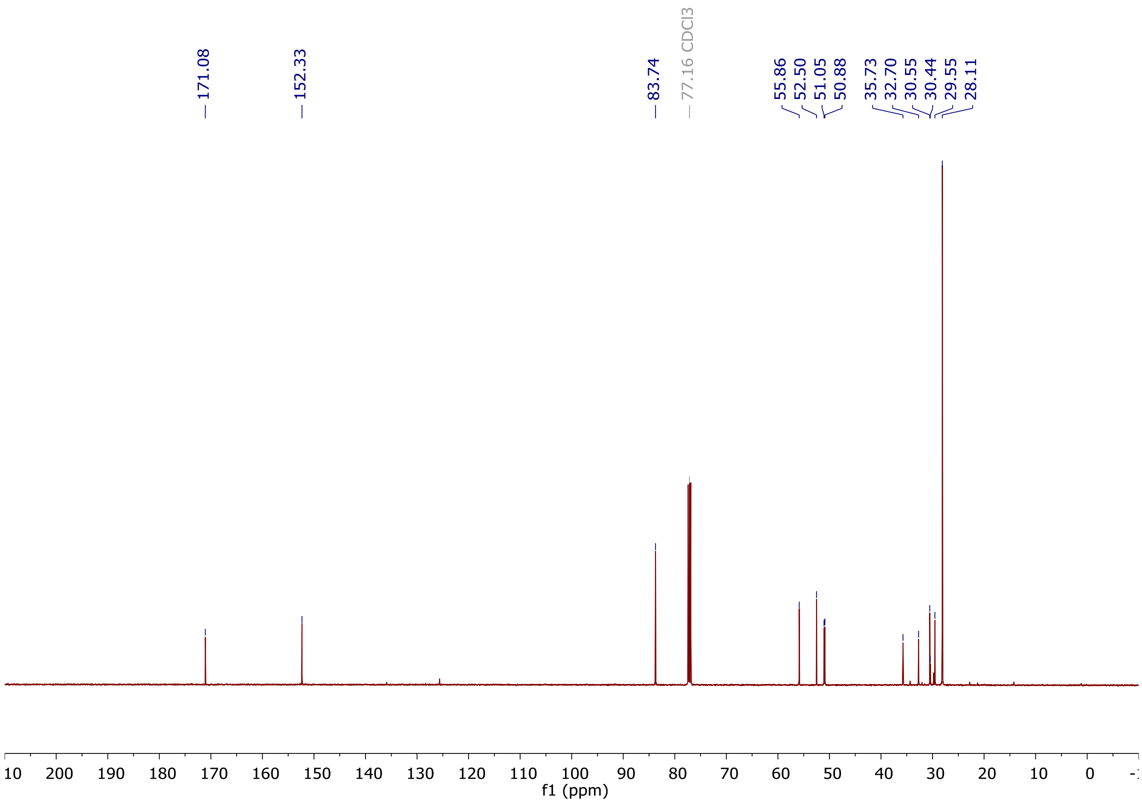

^{1}H-NMR (300 MHz, CDCl3) of compound **4da**

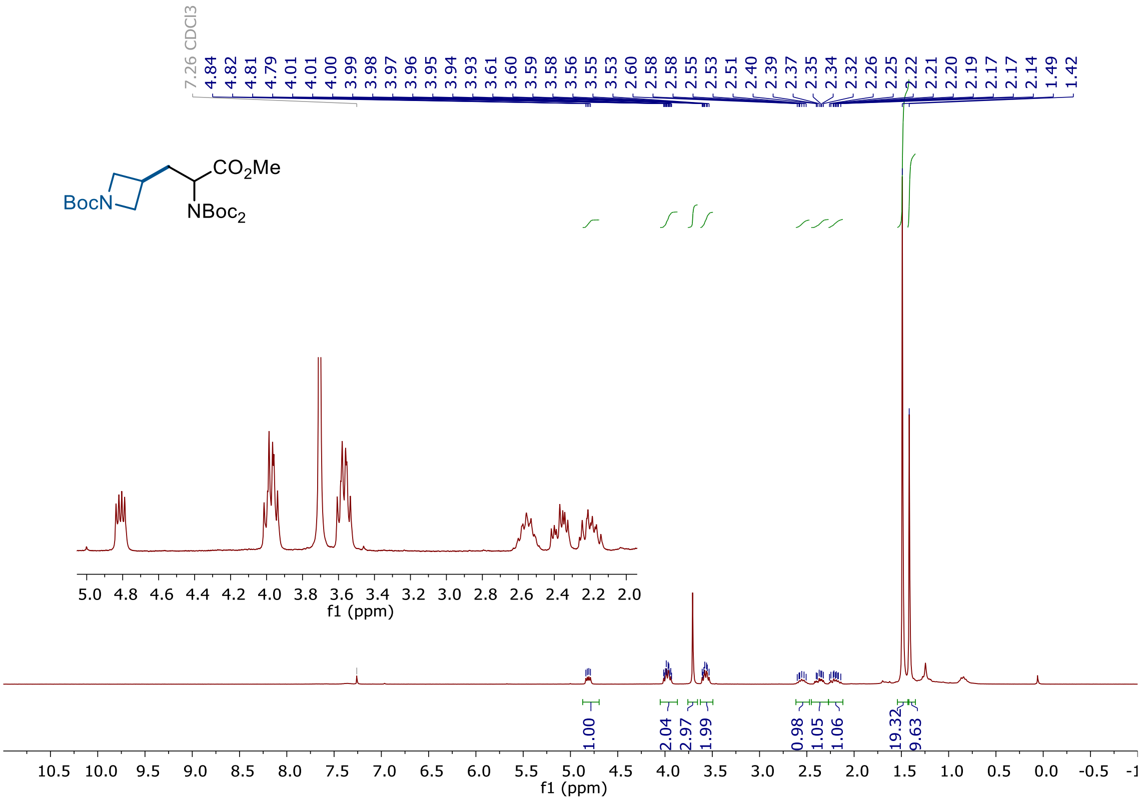

^{1}H-NMR (500 MHz, $CDCl_3$) of compound **4ea**

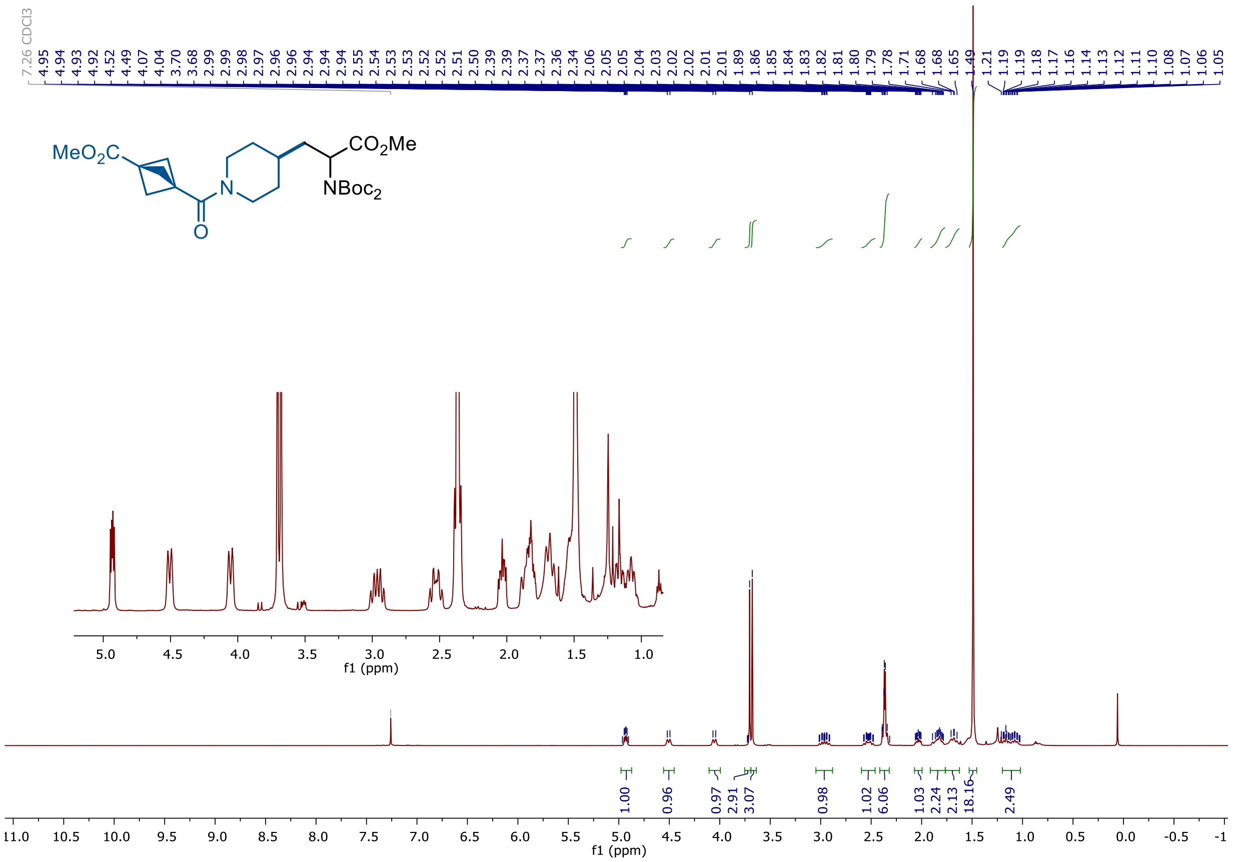

^{13}C-NMR (126 MHz, $CDCl_3$) of compound **4ea**

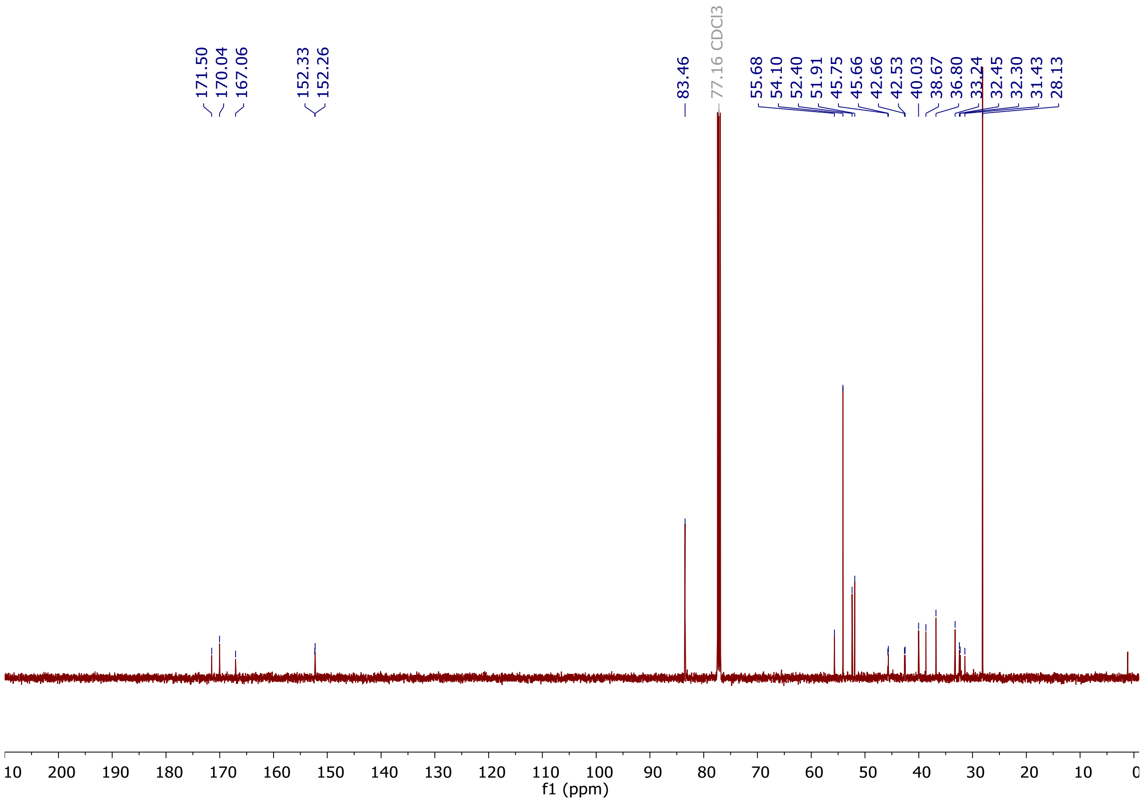

^{1}H-NMR (500 MHz, $CDCl_3$) of compound **4fa**

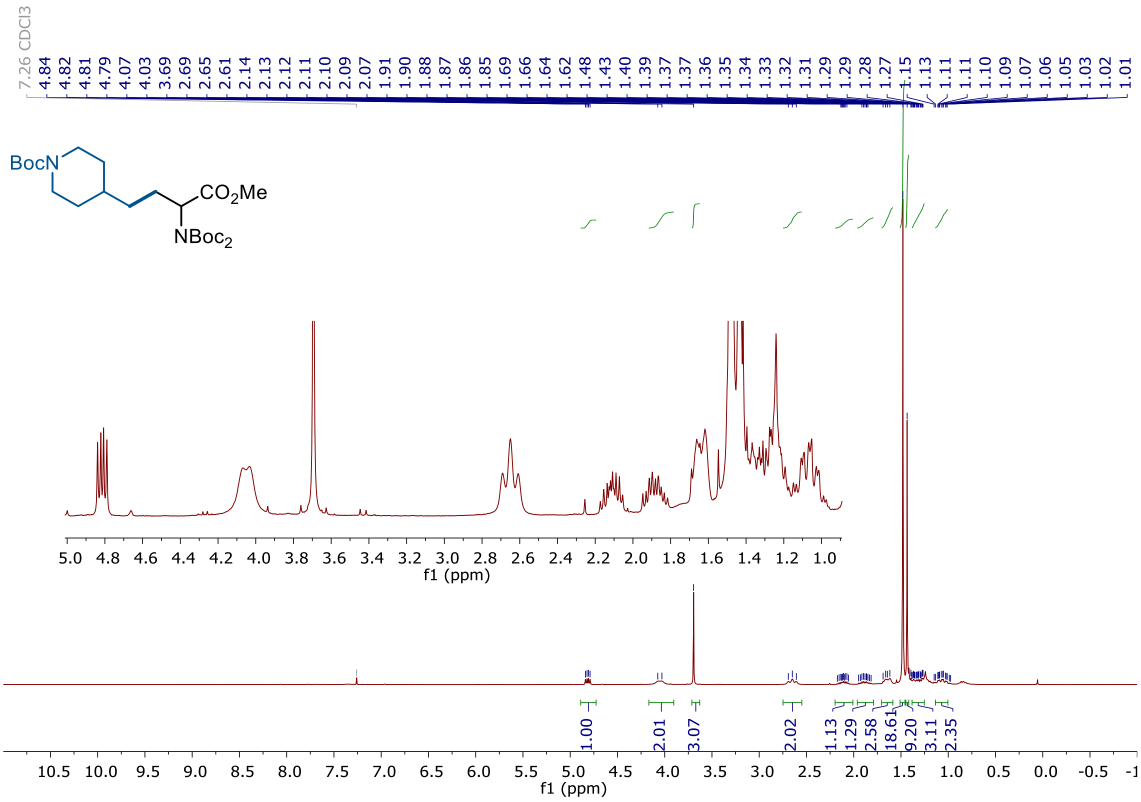

^{13}C-NMR (126 MHz, $CDCl_3$) of compound **4fa**

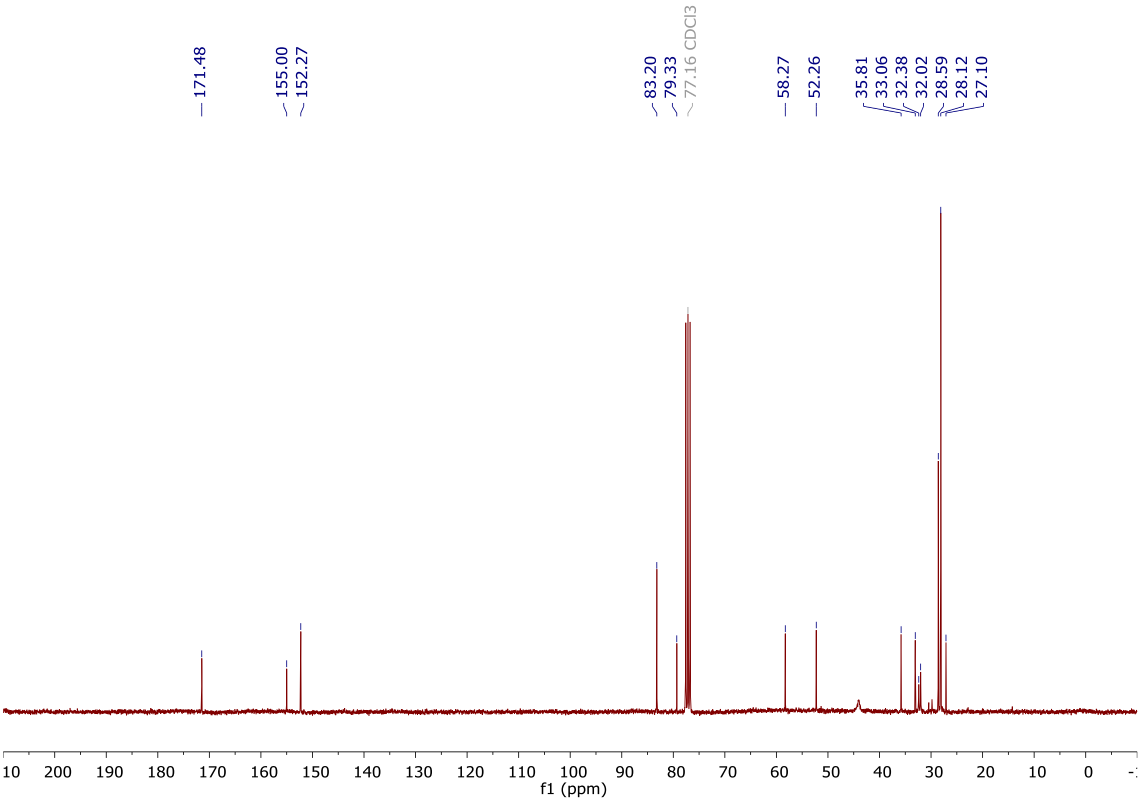

^{1}H-NMR (300 MHz, $CDCl_3$) of compound **4ga**

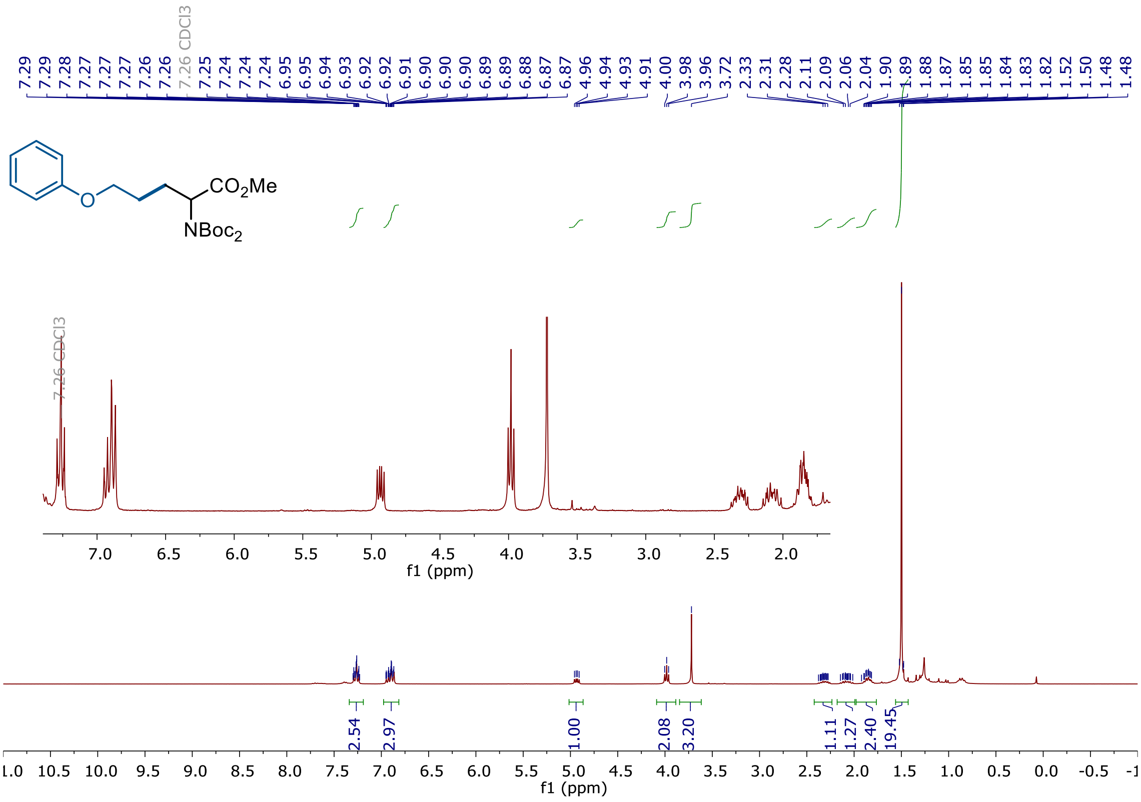

^{13}C-NMR (75 MHz, $CDCl_3$) of compound **4ga**

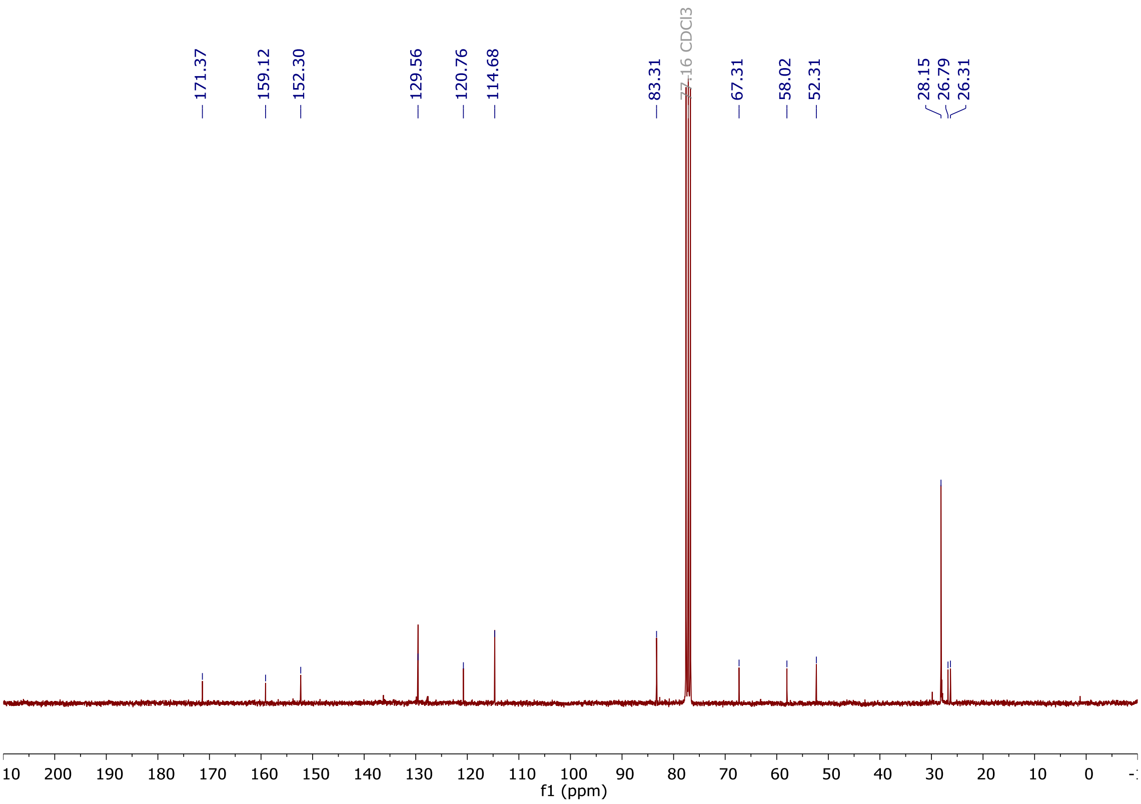

^{1}H-NMR (300 MHz, $CDCl_3$) of compound **4ha**

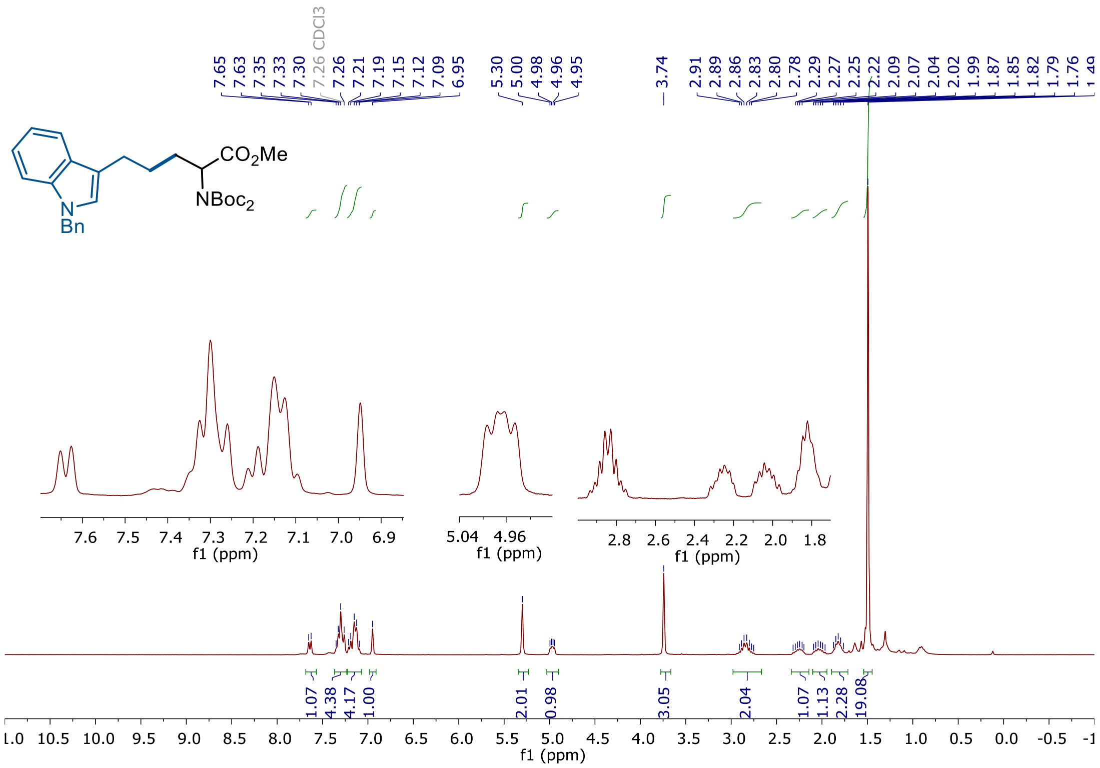

^{13}C-NMR (75 MHz, $CDCl_3$) of compound **4ha**

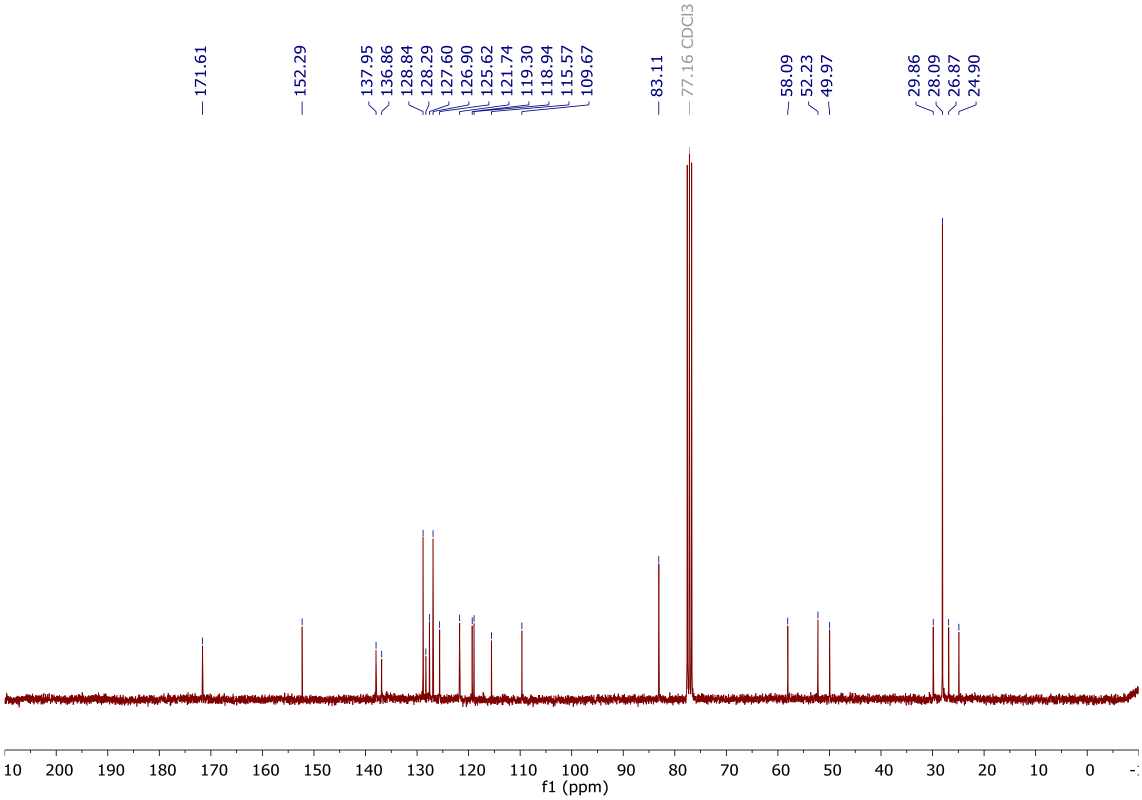

^{1}H-NMR (300 MHz, CDCl3) of compound **4ia**

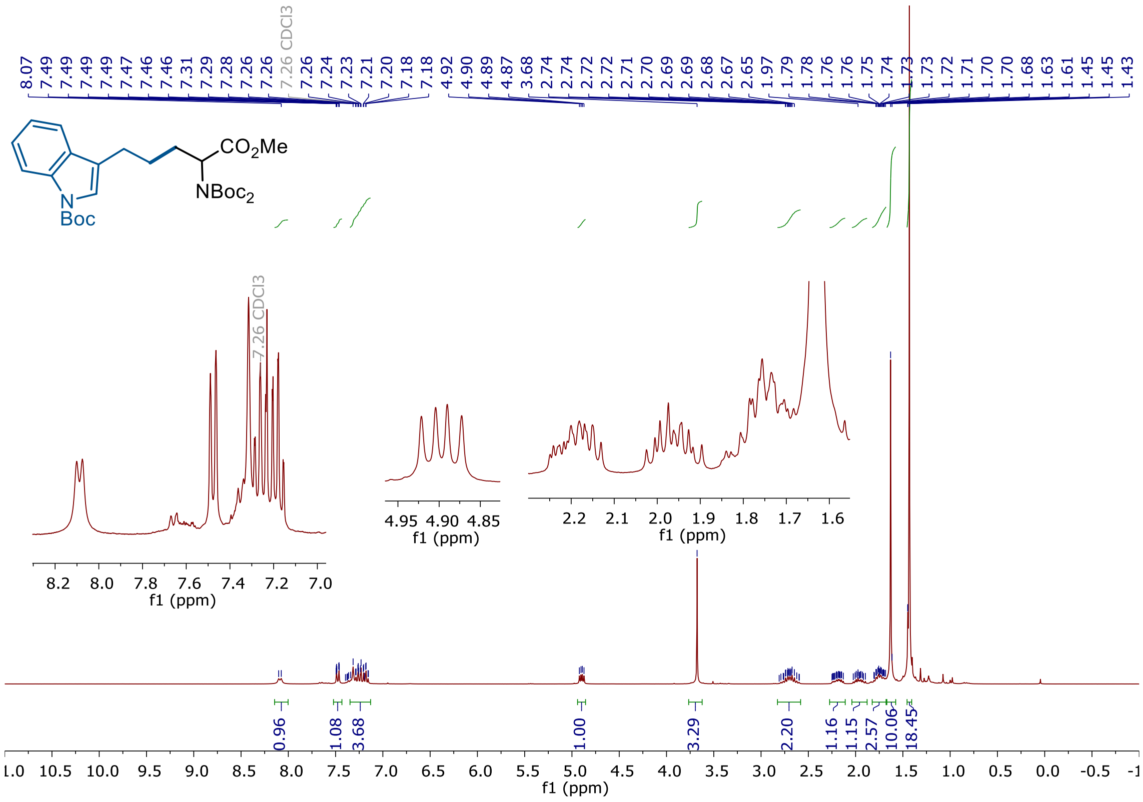

^{13}C-NMR (75 MHz, CDCl3) of compound **4ia**

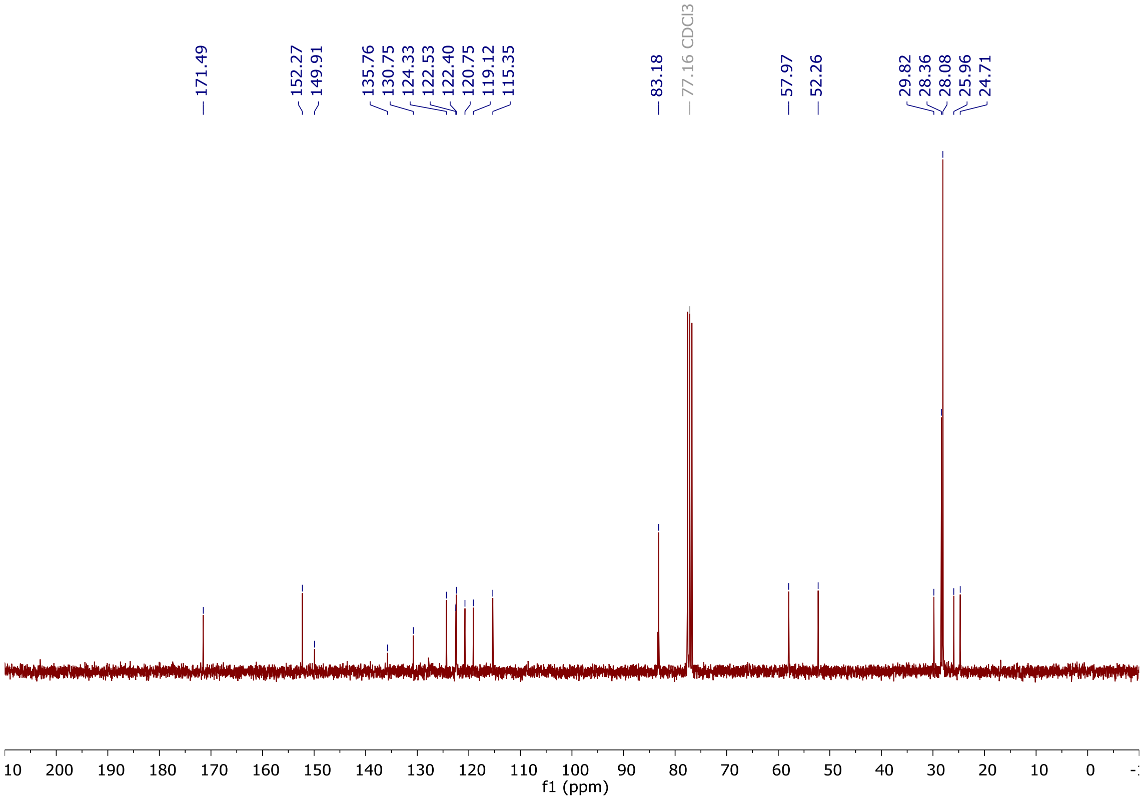

^{1}H-NMR (500 MHz, CDCl$_3$) of compound **4ja**

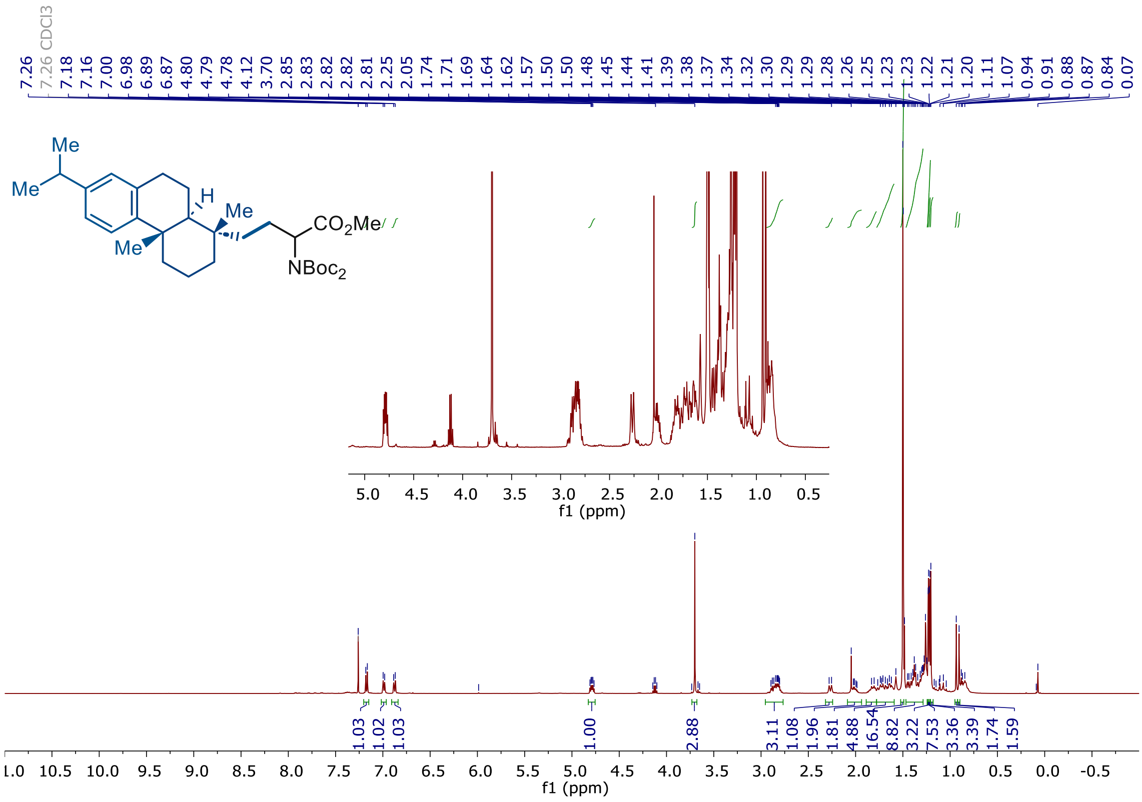

^{13}C-NMR (75 MHz, CDCl$_3$) of compound **4ja**

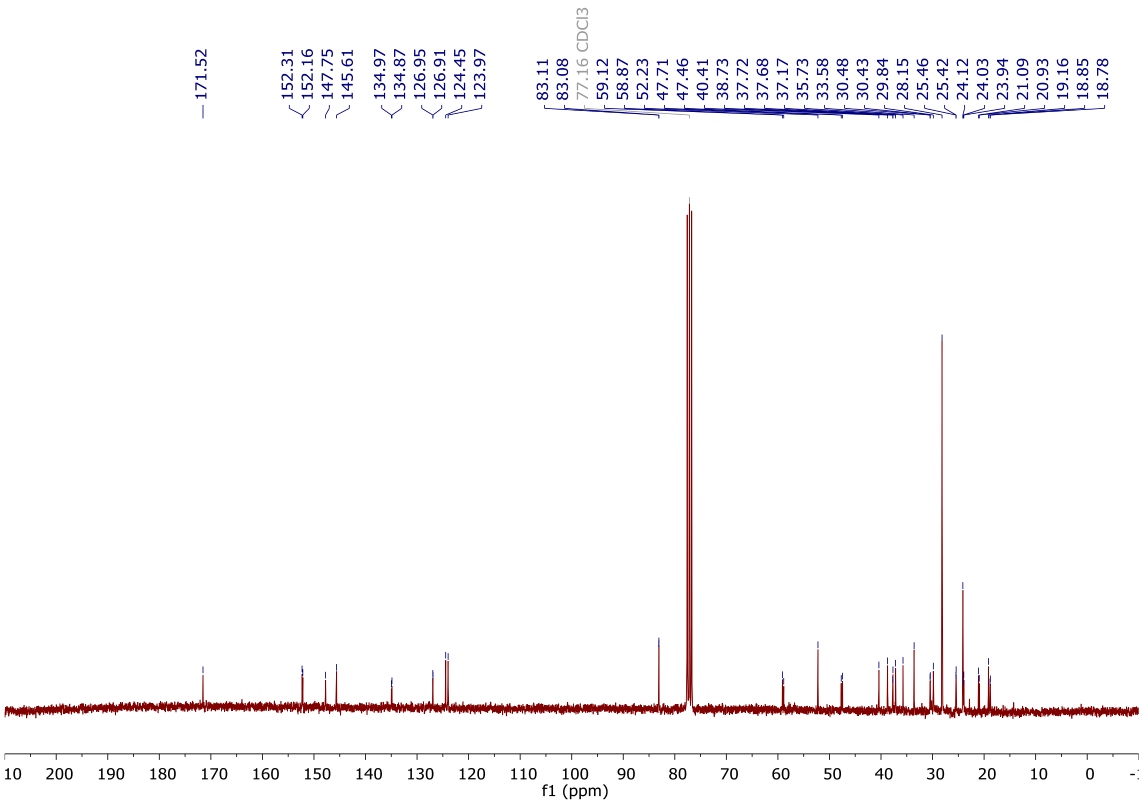

^{1}H-NMR (300 MHz, CDCl3) of compound **4ka**

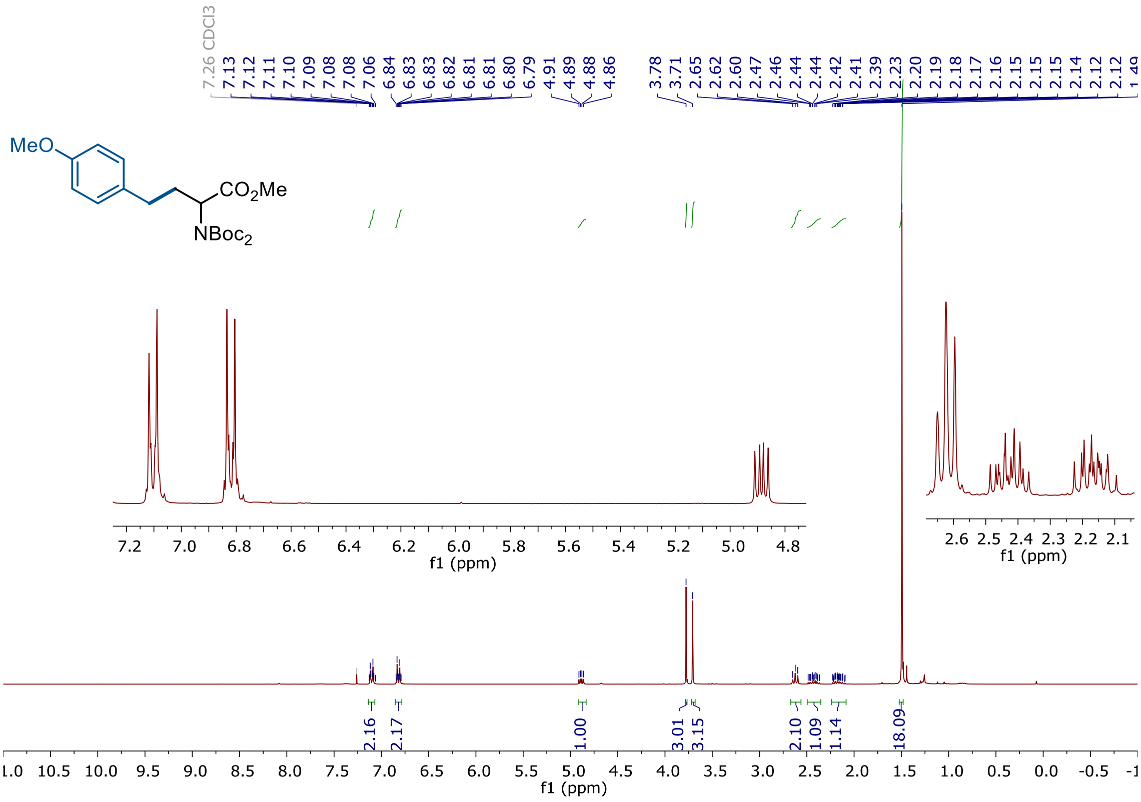

^{13}C-NMR (75 MHz, CDCl3) of compound **4ka**

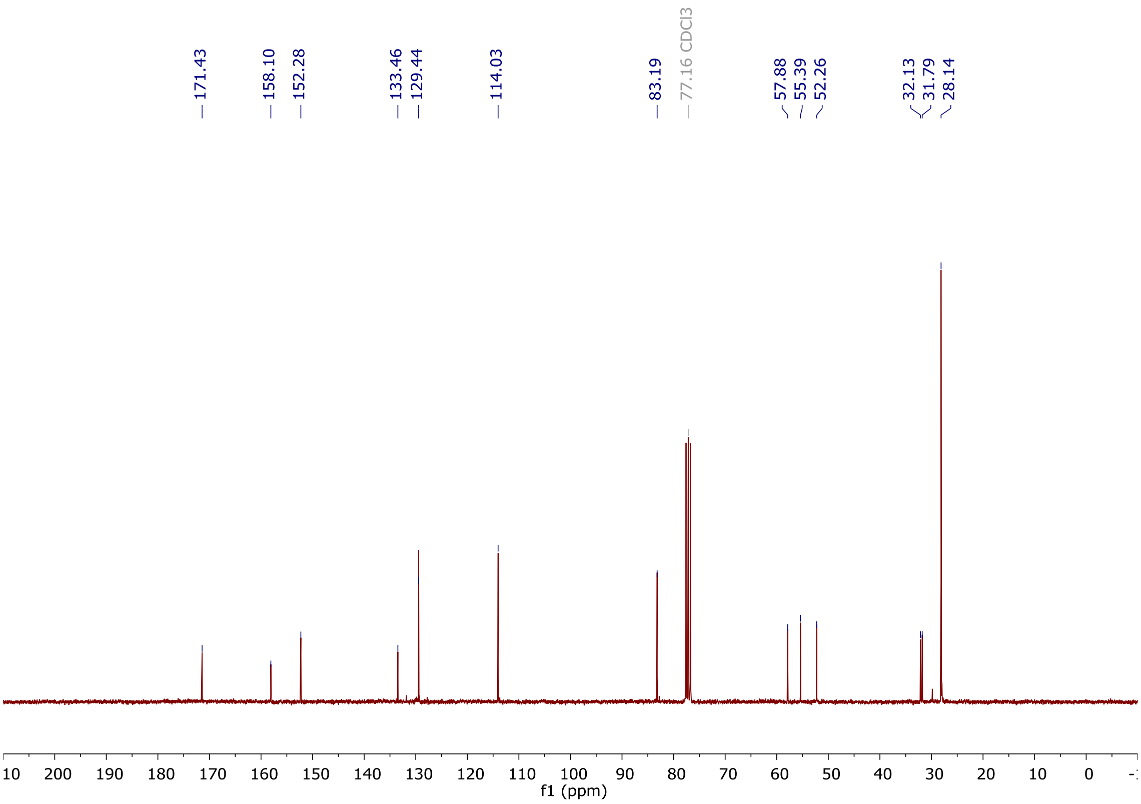

[1]**H-NMR** (300 MHz, CDCl3) of compound **4ma**

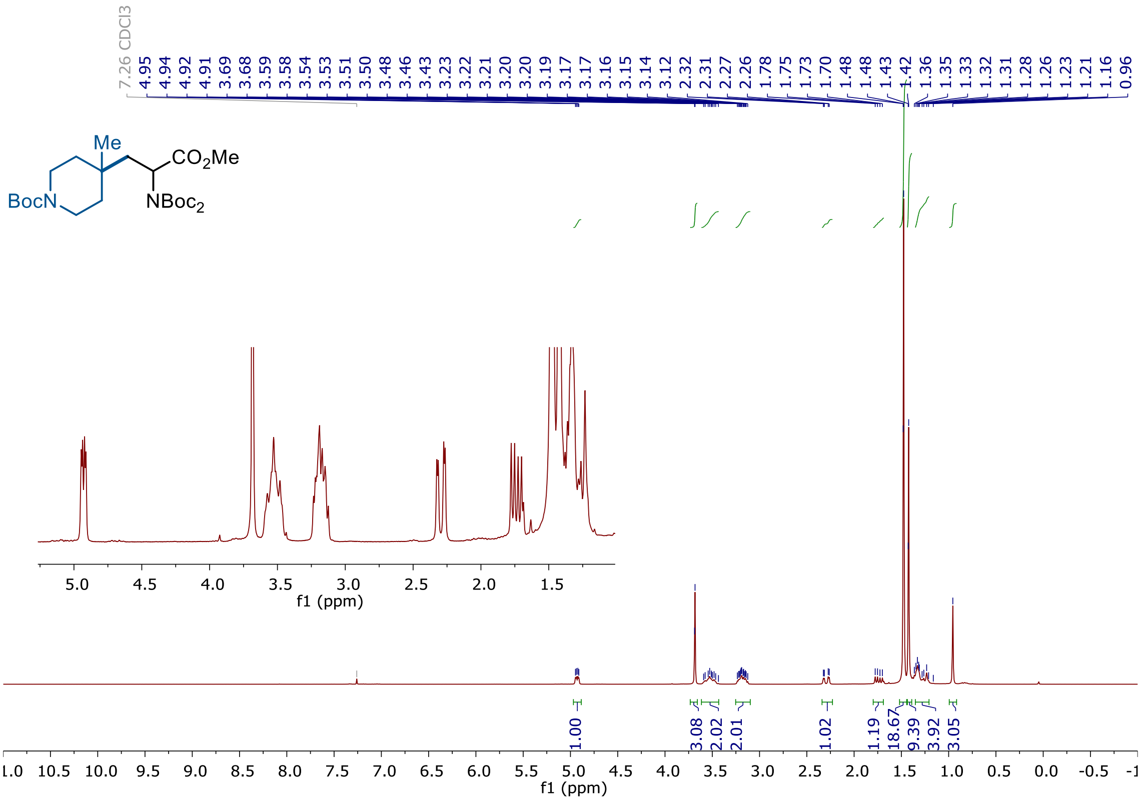

[1]**H-NMR** (300 MHz, CDCl3) of compound **4na**

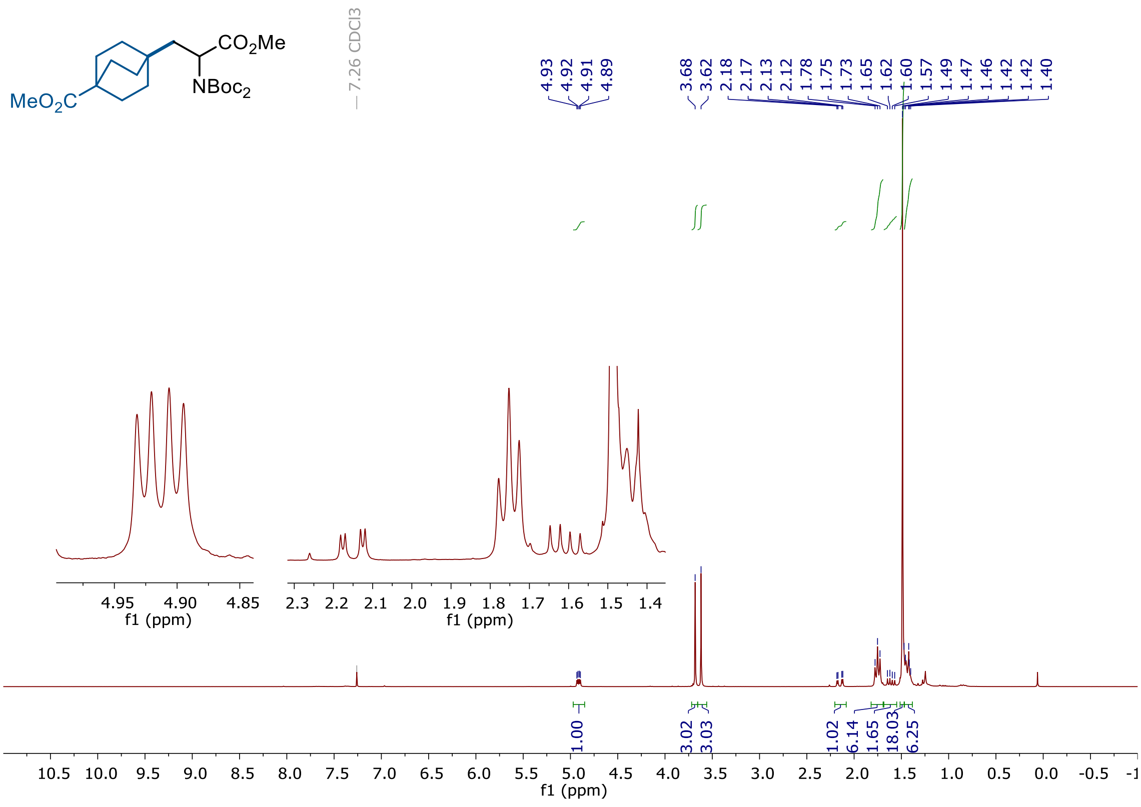

^{1}H-NMR (300 MHz, CDCl3) of compound **4oa**

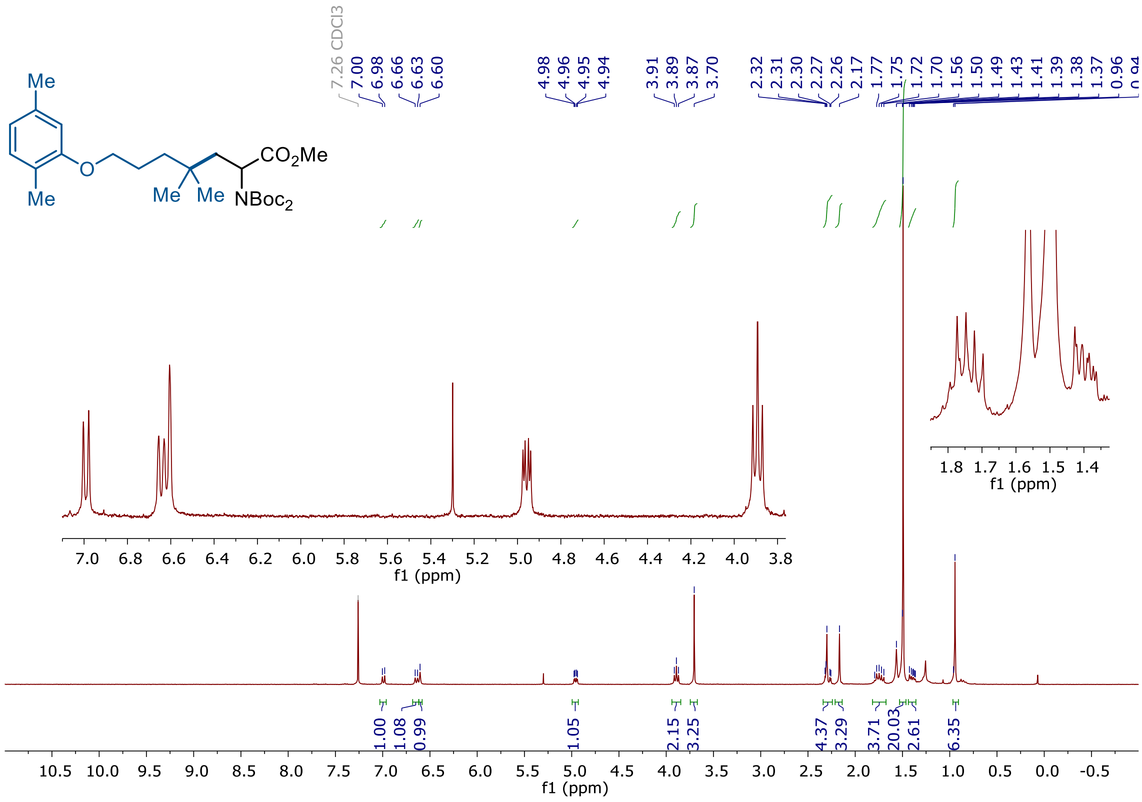

^{1}H-NMR (300 MHz, CDCl3) of compound **4ab**

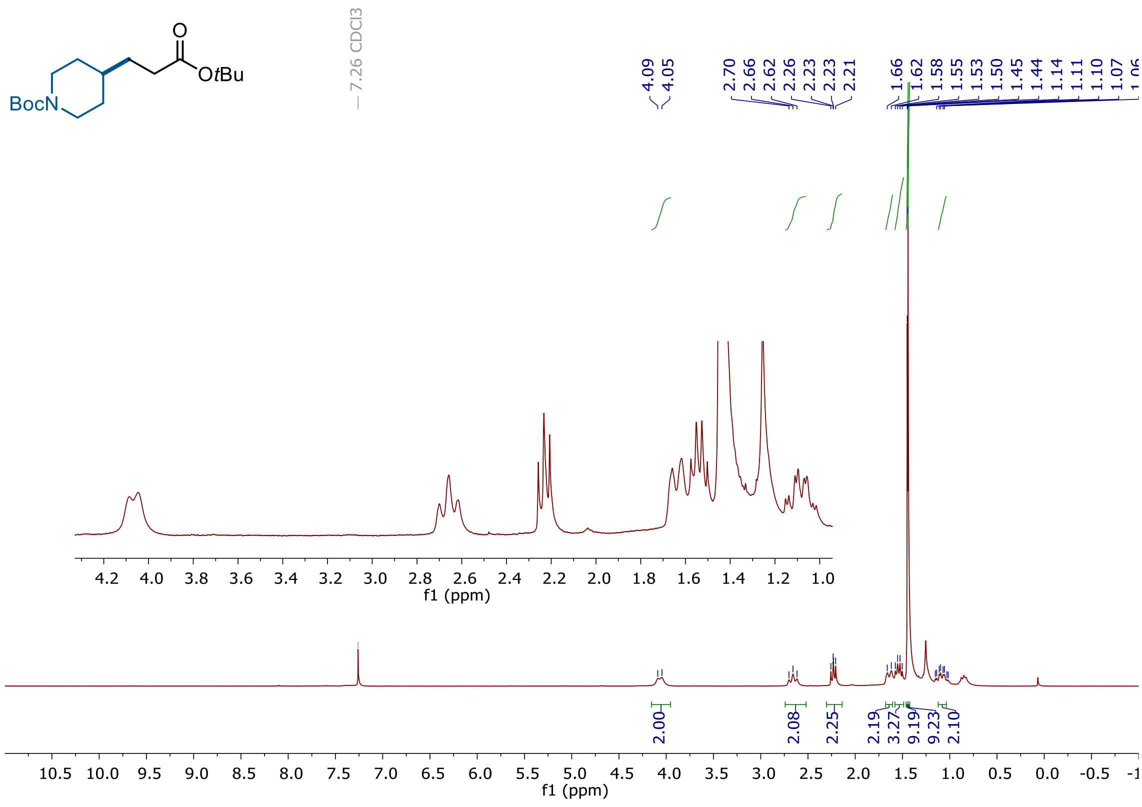

^{1}H-NMR (300 MHz, CDCl$_3$) of compound **4ac**

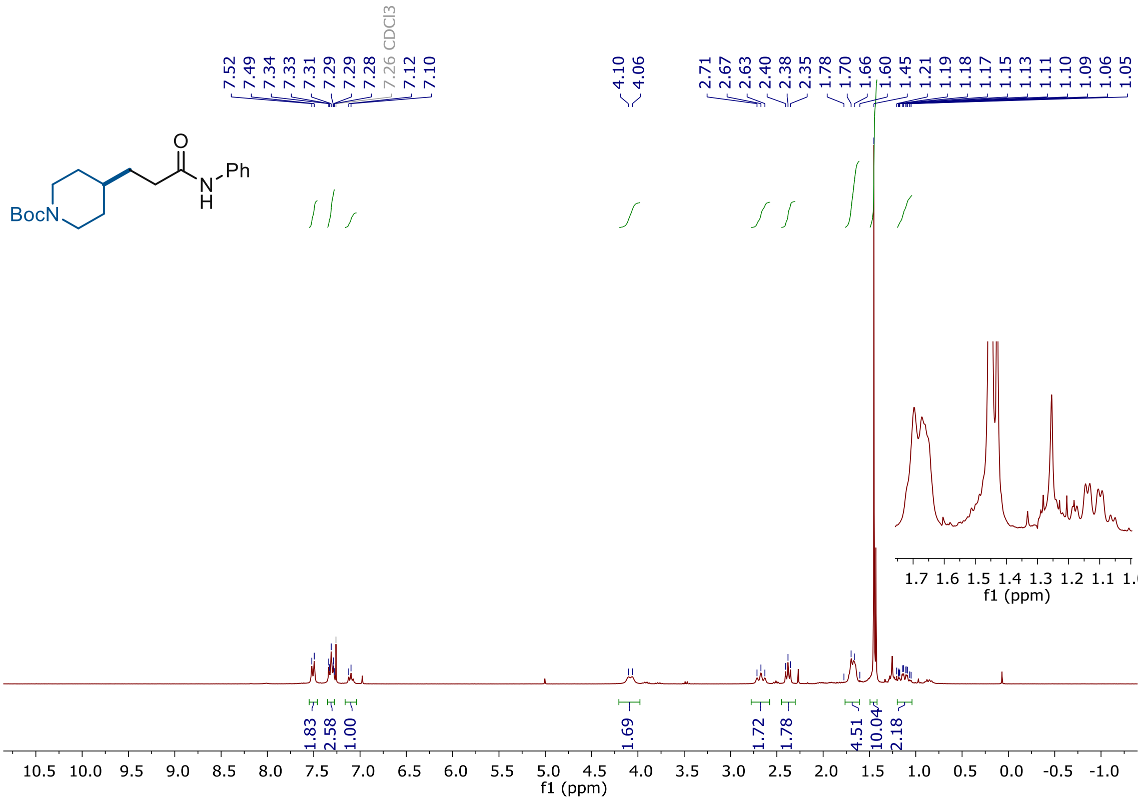

[1]**H-NMR** (500 MHz, Tetrachloroethane-d2, 408 K) of compound **4ad**

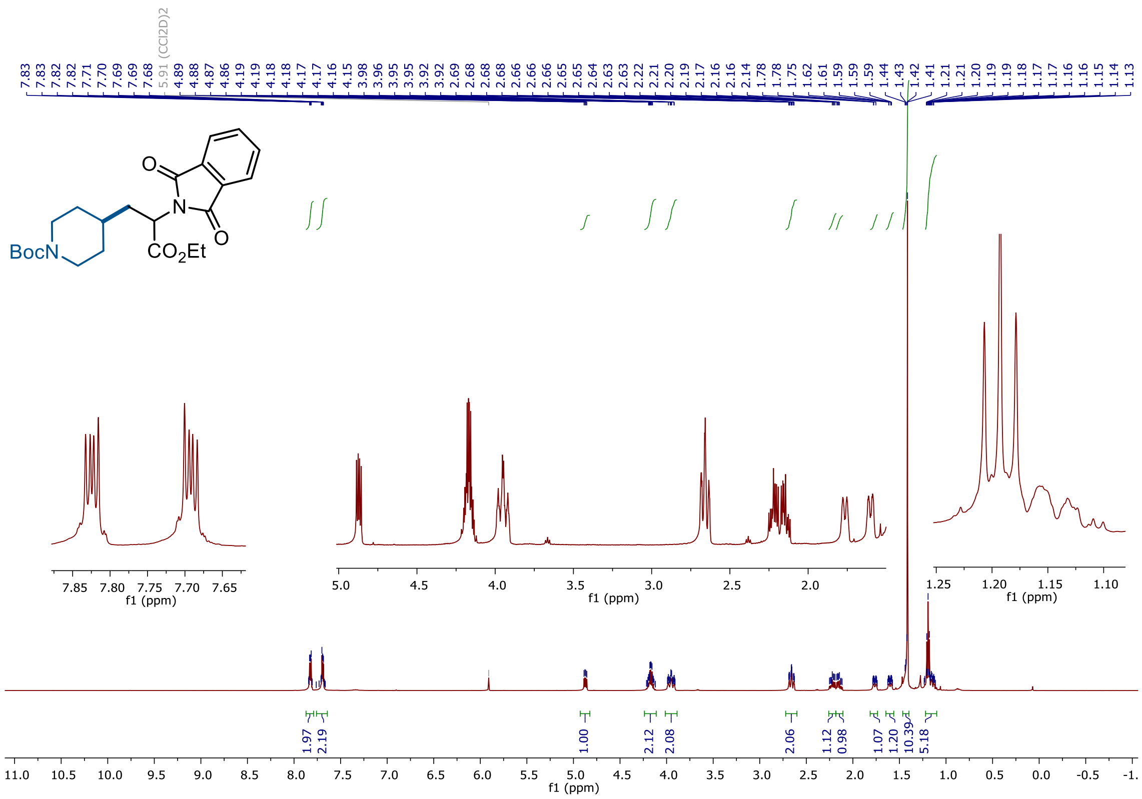

[13]**C-NMR** (126 MHz, Tetrachloroethane-d2, 408 K) of compound **4ad**

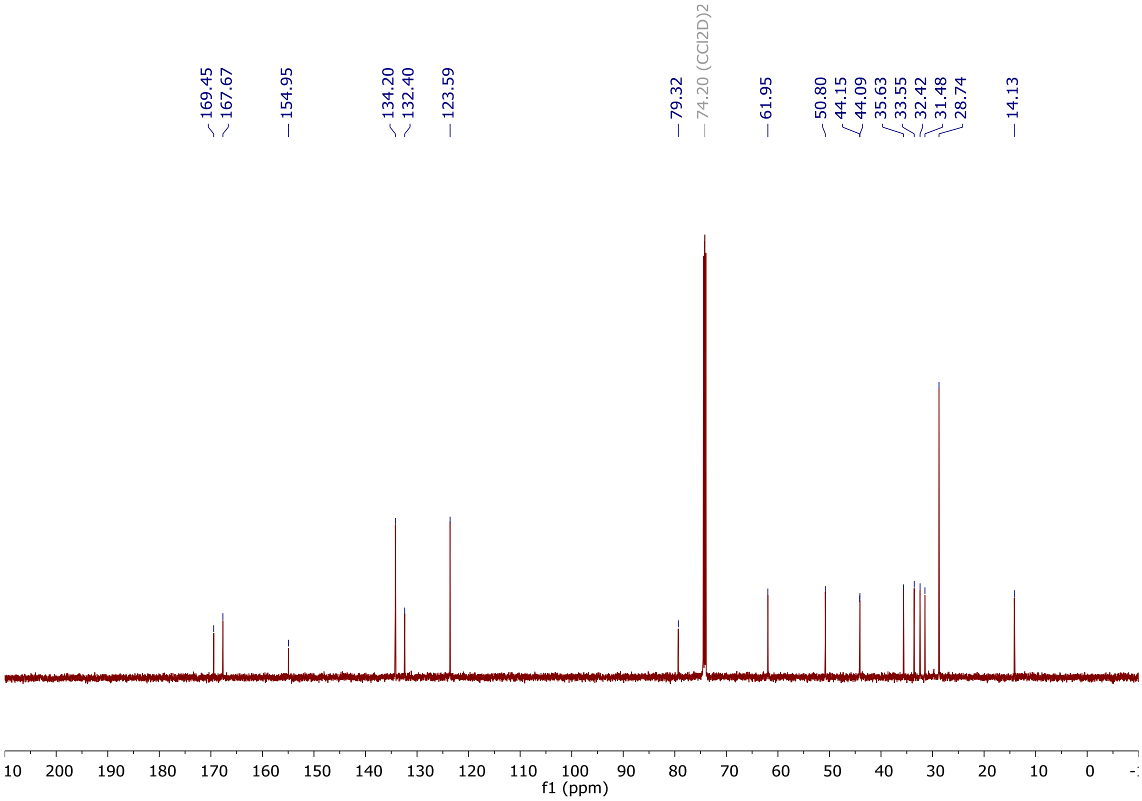

^{1}H-NMR (300 MHz, CDCl3) of compound **4ae**

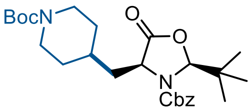

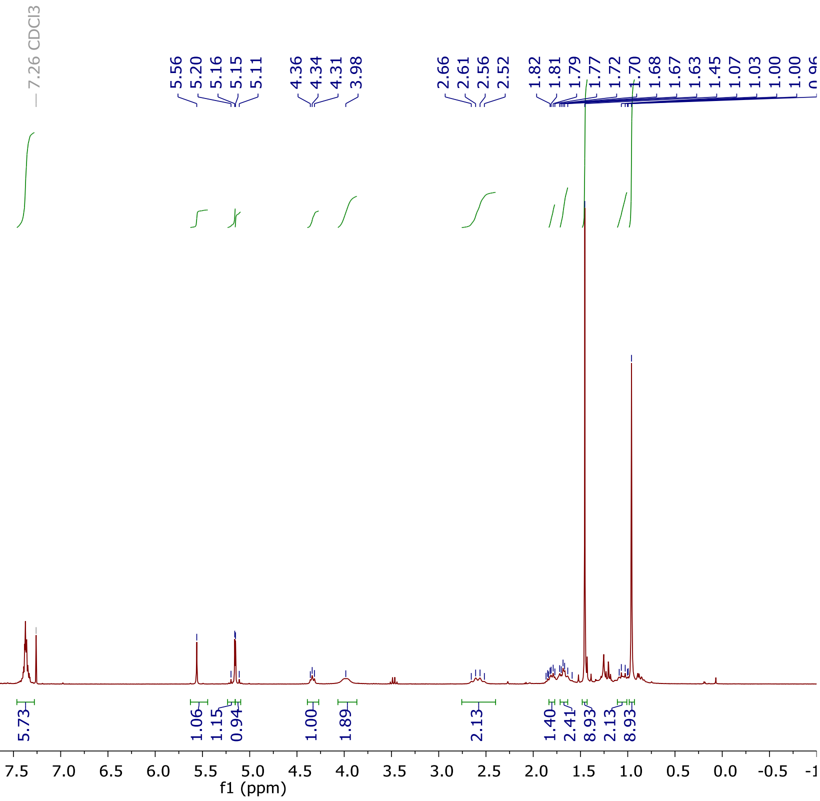

[1]**H-NMR** (500 MHz, Tetrachloroethane-d2, 363 K) of compound **4pe**

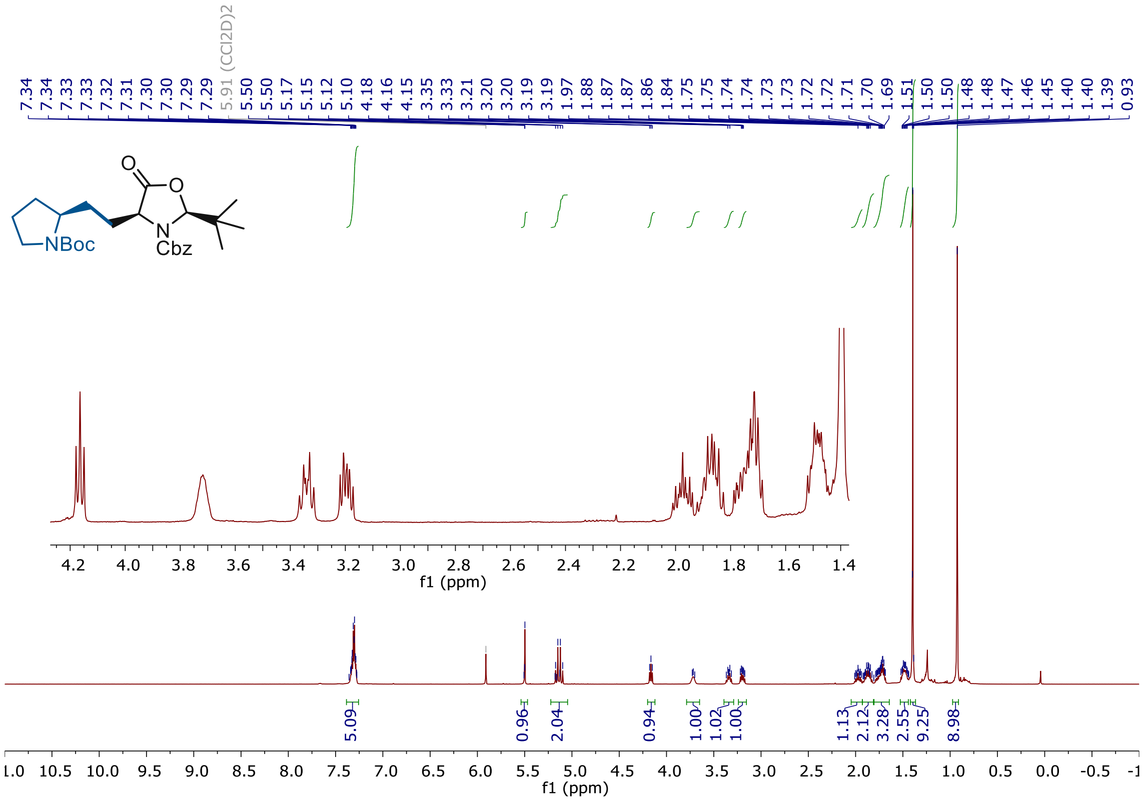

[13]**C-NMR** (126 MHz, 500 MHz, Tetrachloroethane-d2, 363 K) of compound **4pe**

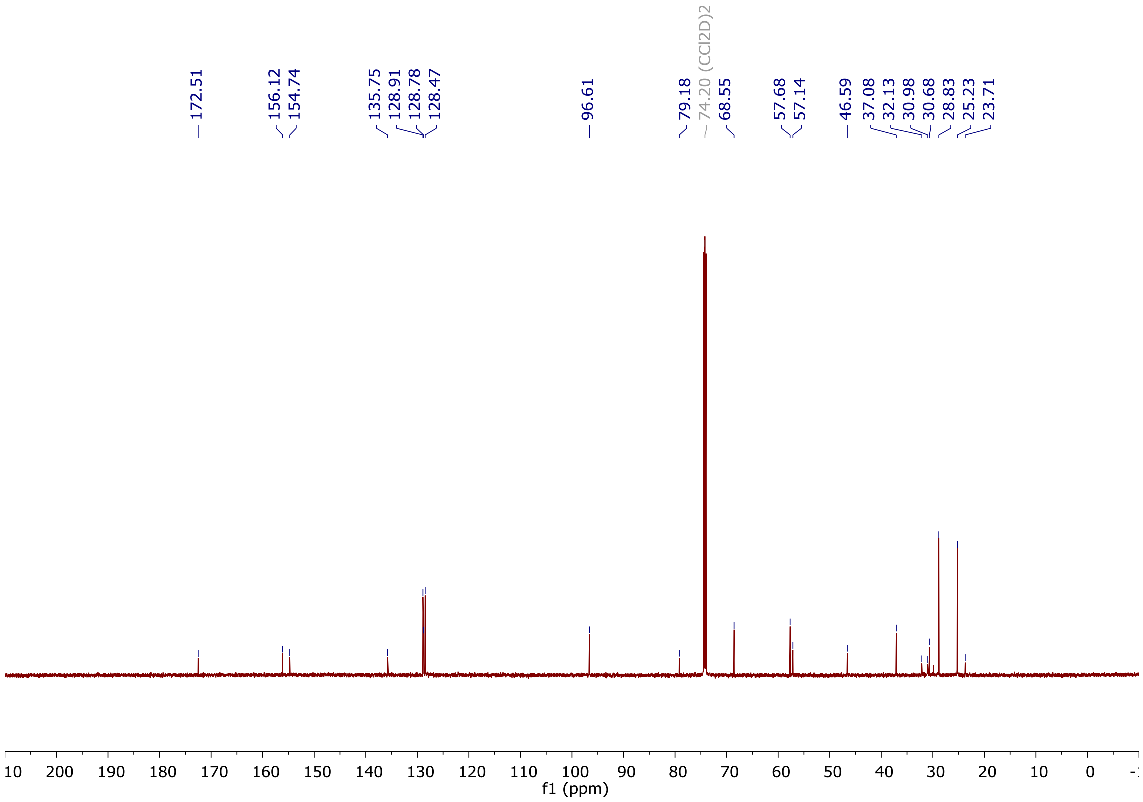

^{1}H-NMR (500 MHz, CDCl$_3$) of compound **4qe**

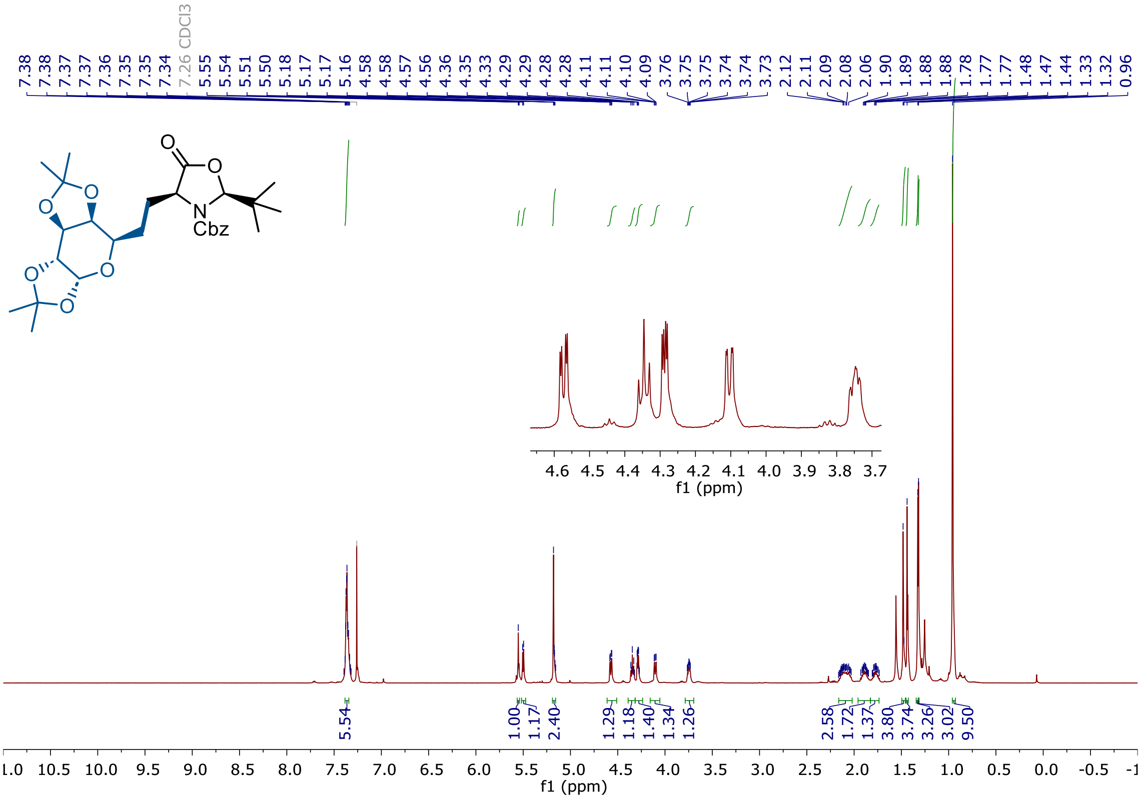

^{13}C-NMR (126 MHz, CDCl$_3$) of compound **4qe**

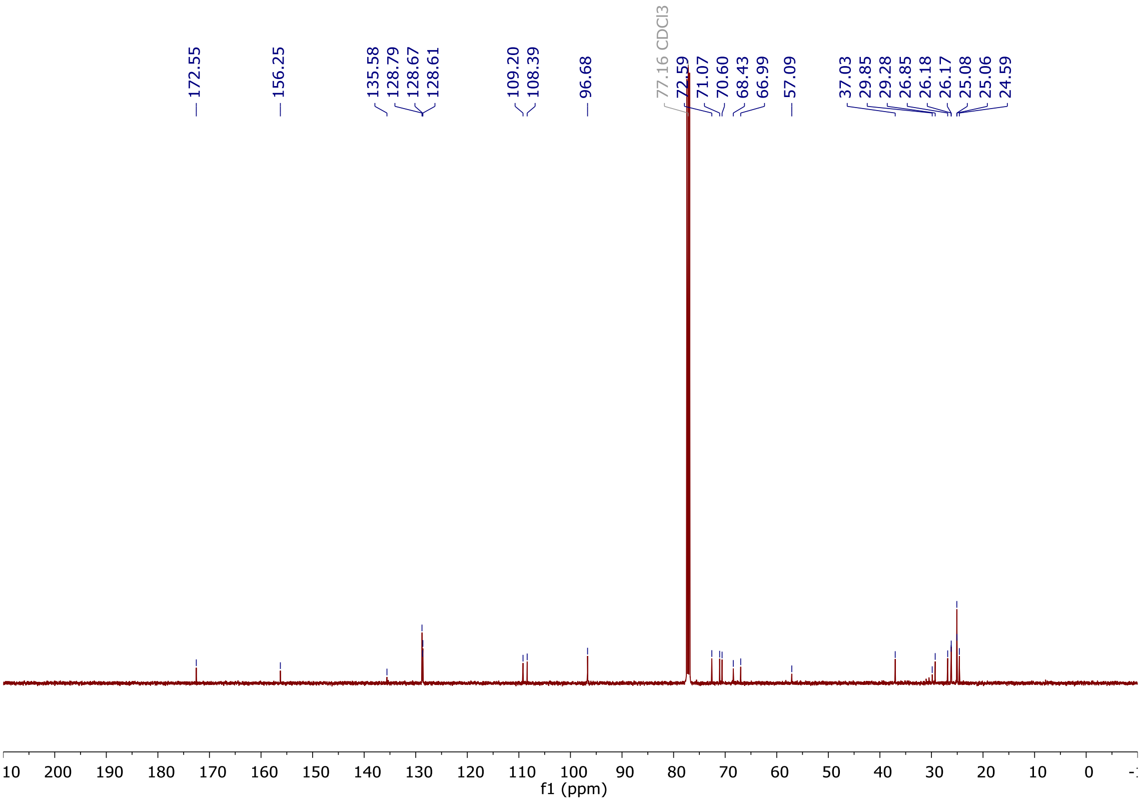

^{1}H-NMR (300 MHz, $CDCl_3$) of compound **5c**

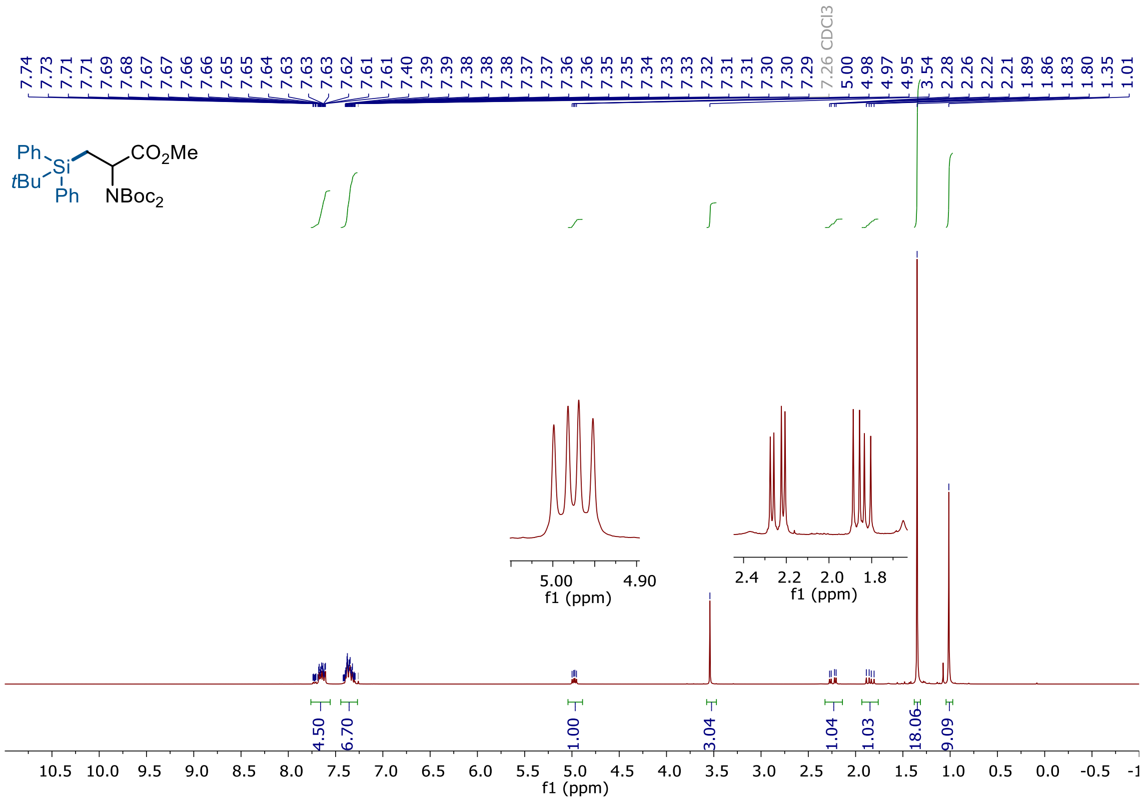

Supplement: Supplementary file 1 [file ol4c04214_si_001.pdf]
